# Occurrence, distribution, and levels of Polychlorinated Biphenyls (PCB), Polychlorinated Dibenzo-p–Dioxins (PCDD), and Polychlorinated Dibenzofurans (PCDF) in fish from the Antioquia Region, Colombia

**DOI:** 10.1007/s10661-025-13956-0

**Published:** 2025-04-16

**Authors:** Boris Santiago Avila, Diana Pemberthy-Mendoza, Henry Zúñiga-Benítez, Gustavo A. Peñuela

**Affiliations:** 1 Facultad de Ingeniería, Grupo Diagnostico y Control de La Contaminación - GDCON, Sede de Investigación Universitaria (SIU), Universidad de Antioquia - UdeA, Calle 70 # 52 -21, 050010 Medellín, Colombia; 2 Departamento de Ingeniería Química, Facultad de Ingeniería, Universidad de Antioquia - UdeA, Calle 70 # 52-21, 050010 Medellín, Colombia

**Keywords:** Biomonitoring, Environmental monitoring, Food safety, Persistent organic pollutants, South America

## Abstract

Persistent organic pollutants (POPs), including polychlorinated biphenyls (PCB), polychlorinated dibenzo-p-dioxins (PCDD), and polychlorinated dibenzofurans (PCDF), are a class of chemical compounds with high persistence that can accumulate in living organisms, mainly in fatty tissues. In addition, exposure to them can cause a wide variety of health problems and adverse effects on humans and wildlife. Therefore, this work aimed to assess the occurrence, distribution, and levels of PCB, PCDD and PCDF in fish samples from the Antioquia region, Colombia. In total, 90 samples of different fish species were evaluated. The levels of the six indicator PCBs were in a range between 26.6 pg$\cdot$g^−1^ w.w (1.33 ng$\cdot$g^−1^ fat) and 4550 pg$\cdot$g^−1^ w.w (236.0 ng⋅g^−1^ fat), the mean value was 395 pg$\cdot$g^−1^ w.w (16.7 ng⋅g^−1^ fat) and the median was 186 pg$\cdot$g^−1^ w.w (8.24 ng⋅g^−1^ fat). The levels of dioxin-like PCBs were between 3.09 pg$\cdot$g^−1^ w.w (0.135 ng⋅g^−1^ fat) and 567 pg$\cdot$g^−1^ w.w (33.9 ng⋅g^−1^ fat), the mean value was 47.2 pg$\cdot$g^−1^ w.w (2.30 ng⋅g^−1^ fat) and the median was 15.5 pg$\cdot$g^−1^ w.w (0.811 ng⋅g^−1^ fat). The mean and median of the PCB toxic equivalent values were 46.6 fg$\cdot$TEQ$\cdot$g^−1^ w.w and 12.0 fg$\cdot$TEQ$\cdot$g^−1^ w.w, respectively. In all cases, PCDD/PCDF were below the analytical method quantification limits. In summary, PCB and PCDD/PCDF levels in selected fish corresponded to background levels and no hot spots were observed. Finally, this study provided an overview of the current situation regarding the presence of these types of pollutants in the region, which could support future research.

**Graphical abstract:**

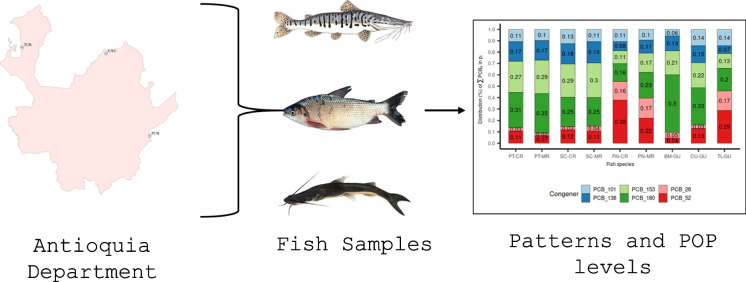

**Supplementary Information:**

The online version contains supplementary material available at 10.1007/s10661-025-13956-0.

## Introduction

Persistent organic pollutants (POPs) have long-range transport through air, water, and migratory species. They can accumulate in living organisms, mainly in fatty tissues, and cause a wide variety of health problems and adverse effects including carcinogenicity effects, birth defects, reproductive and developmental disturbances, neurotoxicity, and suppression of the immune system, among others (Alharbi et al., 2018; Assey & Mogusu, 2023; Camacho-Jiménez et al., 2023; Dev et al., 2023; Reddy et al., 2019; Schecter, 2012). Additionally, decreases in several populations of wild species have been observed as a result of the toxic effects of POPs presence in the environment (Berg et al., 2016; Vasseur & Cossu-Leguille, 2006). Furthermore, given their physicochemical properties, they are globally distributed and have been found even in pristine environments such as the Arctic, the Antarctic, and glacial regions, very far from their origin and places where they were used (Bhardwaj et al., 2018; Goerke et al., 2004; Muir & De Wit, 2010; Rigét et al., 2019).

Considering the above issues, the Stockholm Convention on Persistent Organic Pollutants was adopted in 2001 and entered into force in 2004. As of October 2023, the Convention has 186 parties and includes 34 POPs. Each POP can be a single compound, several congeners, or a family of compounds (Stockholm Convention, 2023). There are restrictions on their production, use and trade on a global scale to protect human health and the environment (Stockholm Convention, 2001). Colombia adopted the Stockholm convention through Law 1196 of 2008.

The POPs listed in the Stockholm Convention are agrochemicals, industrial or unintentionally produced chemicals (Li et al., 2023; Shen et al., 2021; Zhang et al., 2017). Exposure to them can occur through multiple pathways, such as ingestion of contaminated food and water, inhalation, contact with polluted soil, water, or materials, and via breastfeeding (Avila et al., 2022a, 2022b, 2022c, 2022d; Fiedler et al., 2023; Harrad, 2009; Reddy et al., 2019; Torres-Moreno et al., 2023). Fish intake is the one of the main routes of human exposure to POPs (EFSA, 2010; Perelló et al., 2012). Therefore, in countries with high fish consumption, knowing the levels of POPs in fish is of great importance due to its potential impact on human health.

Furthermore, different reports have indicated that sources of emission from several POPs in the last 30 years have been transferred from industrialized to developing countries (Harrad, 2009; Lohmann et al., 2007; Minh et al., 2006; Tanabe, 2007). Moreover, in Colombia there are some studies of air of Andean cities (Aristizábal et al., 2011; Avila et al., 2022a, 2022b, 2022c, 2022d; Cortés et al., 2014, 2016) which found that contamination by rainfall from air to forest and rivers could occur because rainfall can transport POPs from the atmosphere to rivers, especially in high rainfall seasons, carrying pollutants into waterways (Lin et al., 2024). The main POPs transferred from industrialized to developing countries (Reisman, 2023) are organochlorine pesticides (OCP), used in agriculture and pest control; polychlorinated biphenyl (PCB), used in capacitors and transformers; polychlorinated dibenzo-p-dioxins (PCDD) and polychlorinated dibenzo furans (PCDF), which are produced unintentionally; and polybrominated diphenyl ethers (PBDEs), used as flame retardants (Liu et al., 2016; Lohmann et al., 2007; Minh et al., 2006). Also, it is important to note that these countries face several challenges in the management of POPs, including the lack of adequate infrastructure for stockpiles and waste, and limited technical capacities for analysis and monitoring (Sheriff et al., 2022). Moreover, there is not enough information regarding the concentration of these types of pollutants in food, water, and other matrices.

Colombia regulations stipulate maximum levels of dioxin and dioxin-like PCBs (dl-PCB) compounds in fish for human consumption as 4.0 pg$\cdot$TEQ$\cdot$g^−1^ w.w (wet weight) to PCDD/PCDF (sum of 17 congeners) and 8.0 pg$\cdot$TEQ$\cdot$g^−1^ w.w for the sum of dl-PCB (PCB 77, PCB 81, PCB 105, PCB 114, PCB 118, PCB 123, PCB 126, PCB 156, PCB 157, PCB 167, PCB 169, PCB 189) and PCDD/PCDF (MINSALUD, 2012). Furthermore, through regulation 2023/95 the European Commission established the maximum level of the sum of dioxins (PCDD/PCDF) as 3.5 pg$\cdot$TEQ$\cdot$g^−1^ w.w; the sum of dioxins and dl-PCB as 6.5 pg$\cdot$TEQ$\cdot$g^−1^ w.w; and the sum of non-dioxin-like PCB at 75 ng$\cdot$g^−1^ w.w. The sum of non-dioxin-like PCB corresponds to the sum of the six indicator PCBs (PCB 28, PCB 52, PCB 101, PCB 138, PCB 153, PCB 180) (EU, 2023).

In Colombia there are 36,464 productive units that carry out aquaculture activities, and around 150,000 fishermen. Total aquaculture and fishing production in Colombia in 2021 was 300,162 tons, along with nearly 25 million units of non-edible ornamental fish destined for export and the national market. The contribution of fisheries and aquaculture to the gross domestic product (GDP) is 0.2% (3.3% of agricultural GDP). Catfish, bass, tilapia, cachama, trout, shrimp, and other native species are the most consumed products.

Considering the above, the aim of this study was to evaluate the levels of selected PCB and PCDD/PCDF in some fish species from the Antioquia region in Colombia. The research was undertaken to establish a baseline regarding the concentrations of these pollutants in the region; and to try to fill the gaps in knowledge regarding the source, occurrence, distribution, and levels of POPs in a developing country.

## Materials and methods

### Standards, reagents, and materials

Native PCB-mix 3 of 10 $\upmu$g$\cdot$mL^−1^ in cyclohexane was purchased from Dr. Ehrenstorfer GmbH (Augsburg, Germany). Additionally, individual PCB native standards (purity$\ge$98%) were purchased from Dr. Ehrenstorfer GmbH (Augsburg, Germany). Stock solutions of 500 $\upmu$g$\cdot$mL^−1^ in hexane were prepared from the individual PCB native standards, and a mixture of 10$\upmu$g$\cdot$mL^−1^ in hexane was made from the individual stock solutions. The labelled standard WP-LCS was purchased from Wellington Laboratories (Ontario, Canada). A labelled PCB work solution of 50 pg$\cdot \mu$L^−1^ in isooctane was prepared from WP-LCS. Solutions of individual PCDD/PCDF native standards at 10 $\mu$g$\cdot$mL^−1^, 50 $\mu$g$\cdot$mL^−1^, and 5.0$\mu$g$\cdot$mL^−1^ in toluene were purchased from Accustandard, Inc (New Haven, USA). Furthermore, standard PCDD/PCDF solutions at 50 $\mu$g$\cdot$mL^−1^ in nonane were purchased from Cambridge Isotope Laboratories, inc (Tewksbury, USA). A work mixture with the 17 PCDD/PCDF congeners was prepared at 0.50 $\upmu$g$\cdot$mL^−1^ in isooctane. The labelled standard EPA-1613LCS was purchased from Wellington Laboratories (Ontario, Canada). A labelled PCDD/PCDF work solution of 5.0 pg$\cdot \mu$L^−1^ in isooctane was prepared from EPA-1613LCS. All solutions were stored at −20 ${}^{\circ }$C$\pm$3 ${}^{\circ }$C. More details of standards are provided in **Supplementary Material**.

Chromatographic grade solvents (n-hexane, acetone, toluene, and isooctane) were purchased from Merck (Darmstadt, Germany). Sulfuric acid (H_2_SO_4_) (98.0% purity), sodium sulfate anhydrous (purity$\ge$99.0%), silver nitrate (AgNO_3_) (purity$\ge$99.0%), and sodium hydroxide (NaOH) (purity$\ge$99%) were purchased from Merck (Darmstadt, Germany). Florisil 100–200 mesh and silica gel 400 mesh (40–63 µm) were obtained from Sigma Aldrich (Burlington, USA). Envi-Carb cartridges (250 mg, 3 mL) were purchased from Sigma Aldrich (Burlington, USA). Deionized water was obtained from a Millipore system.

### Sampling sites

**Fig. 1:**
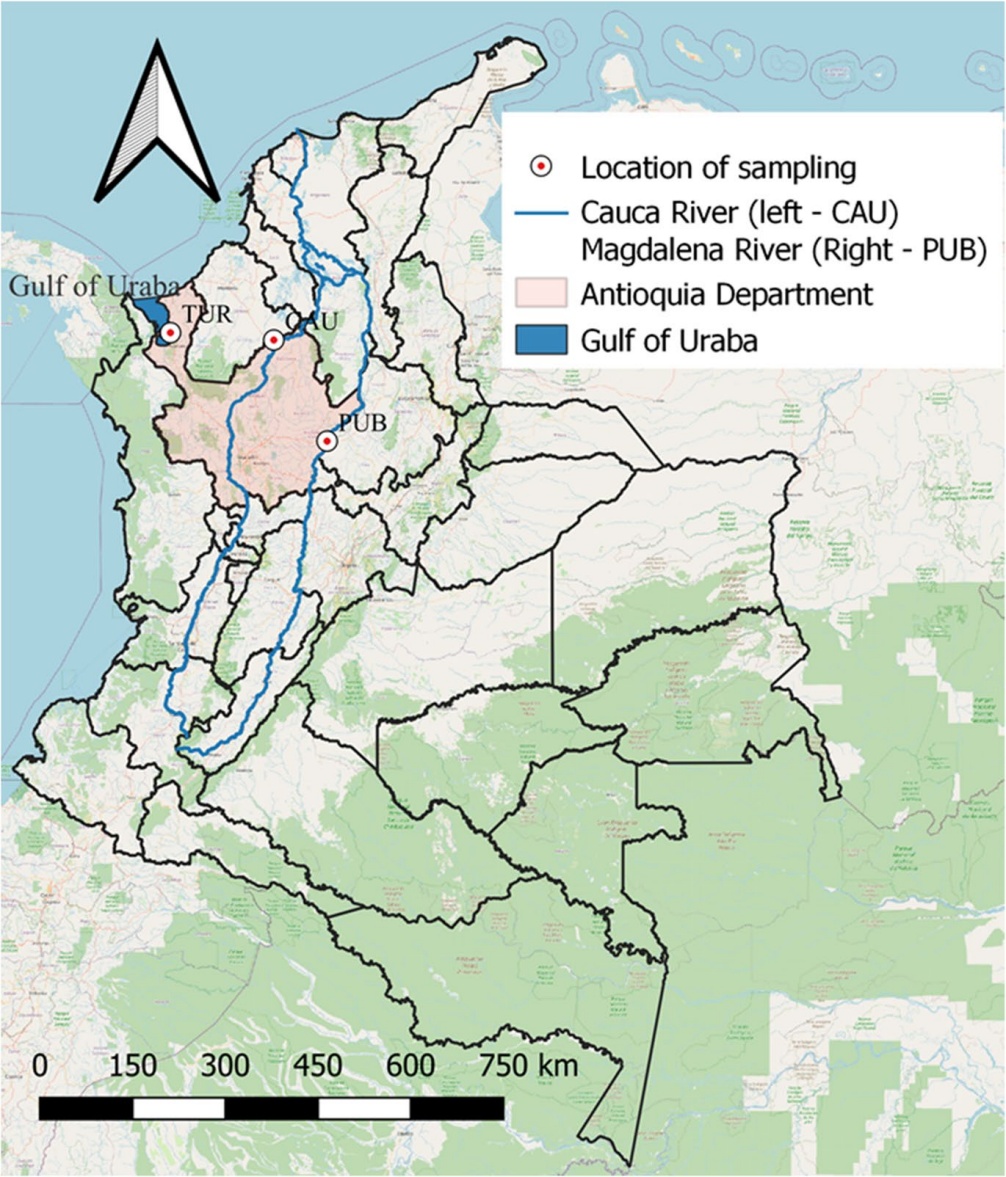
Map of the sampling location. TUR: Turbo, CAU: Caucasia, PUB: Puerto Berrio. The blue polygon shown the Gulf of Uraba, in the pink polygon the Antioquia Region is shown. In the blue lines the Cauca River is shown, which runs through CAU (left blue line), along with the Magdalena River which runs through PUB (right blue line)

The fish samples were collected at three locations in the region of Antioquia, Colombia: Puerto Berrio (PUB), Caucasia (CAU), and Turbo (TUR). PUB (population 51,079) is located in the Middle Magdalena region near the Magdalena River (the largest river in the country). Its economy is mainly based on agriculture, gold mining and fishing. The covering area is 1,184 km^2^. CAU (population 97,803) is located in the Bajo Cauca subregion near the Cauca River (Colombia’s second largest river). Its economy is based on livestock farming, fishing, agriculture, and mining; and it is considered as the country largest gold producer. The covering area is 1,411 km^2^. TUR (population 59,547) is a district in the Urabá subregion near the Gulf of Urabá (Caribbean region), and it is the largest municipality in the region, covering an area of 3,055 km^2^. The main source of income is agricultural, including banana and plantain plantations. Other key activities include natural resources exploitation, fishing, and extensive livestock farming. Figure 1 shows the location of sampling.

The fish species collected were the Magdalena tiger catfish (*Pseudoplatystoma magdaleniatum*) which is a predatory species native from the Magdalena River basin in Colombia. It feeds primarily on smaller fish and crustaceans and is highly valued for its meat. The long-whiskered catfish (*Sorubim cuspicaudus*), found in the Magdalena and Orinoco River basins, is a nocturnal predator that mainly consumes small fish and invertebrates. Meanwhile, the bocachico (*Prochilodus magdalenae*) is an herbivorous-detritivorous species endemic to the Magdalena River basin, feeding on organic detritus, algae, and plant matter, making it essential for nutrient cycling and highly important for local fisheries. The gafftopsail catfish (*Bagre marinus*), which inhabits the coastal and estuarine waters of the western Atlantic, is an opportunistic bottom feeder with a diet consisting of crustaceans, mollusks, and small fish. Common snook (*Centropomus undecimalis*) is a carnivorous predator that preys on smaller fish and crustaceans, thriving in coastal waters, estuaries, and mangrove ecosystems. Finally, the Atlantic cutlassfish (*Trichiurus lepturus*) is a predatory species found in tropical and subtropical waters around the world. It feeds primarily on small fish, squid, and crustaceans, and its long, slender body gives it a distinctive blade-like appearance.

### Sampling

Samples were collected from fish markets located in the selected places. The sampling was carried out in two rounds; the first was in June 2022 and the second in August 2022. In both cases the number of samples was 45 (15 samples from each location and source). In total, 30 fish samples were taken from each place. The species collected from the Magdalena River and the Cauca River were *Pseudoplatystoma magdaleniatum* (PT-MR/PT-CR, respectively), *Sorubim cuspicaudus* (SC-MR/SC-CR, respectively) and *Prochilodus magdalenae* (PM-MR/PM-CR, respectively); and the Gulf of Urabá species were *Bagre marinus* (BM-GU), *Centropomus undecimalis* (CU-GU), and *Trichiurus lepturus* (TL-GU). Five samples of each species by location were taken in each sampling round. Fish length and weight were determined during the sampling. Finally, samples were wrapped with aluminum foil and put in a resealable plastic bag, then transported in a rigid portable cooler with abundant ice to the Pollution Diagnosis and Control Group (GDCON) research group in the city of Medellín. Storage was at −20.0 ± 3.0 °C.

### Samples pre-treatment

The skin and bones of the samples were removed, then the muscle was filleted and homogenized (dorsal spineless-boneless fillets) using a Hobart GmbH CC34 model cutter (Offenburg, Germany). Then, the homogenized samples were placed in a foam food container and stored at −20.0 ± 3.0 °C for at least one week. Samples were freeze-dried using a seven-day cycle in a FreeZone plus 6 instrument (Labconco, USA). Finally, the samples were placed in a resealable plastic bag and stored at −20.0 ± 3.0 °C before extraction and analysis.

### Extraction and clean-up

Fish samples were extracted in a Soxhlet system. Subsequently, the extract was cleaned up in an acid/basic column, and PCB, PCDD, and PCDF were separated from PBDEs in a nitrate column. Then, PCB (**F2**) was separated from PCDD and PCDF (**F3**). Subsequently, **F3** was cleaned in a carbon column (**F4**). Finally, the fractions **F2** (PCB) and **F4** (PCDD/PCDF) were passed into chromatography vials for instrumental analysis (EPA, 1994, 2010; Kiridena et al., 2024; H. Liu et al., 2006; Roszko et al., 2013). More details of columns used are provided in **Supplementary Material**.

20.0 – 25.0 g of freeze-dried sample were weighed in an extraction thimble (the amount of sample varied depending on the species). The weighed sample was spiked with 20.0 µL of 50 pg·$\mu$L^−1^ WP-LCS and 20.0 µL of 5.0 pg·$\mu$L^−1^ EPA-1613LCS work solutions. Then, the sample was extracted in a Soxhlet system with 300.0 mL of hexane:acetone (1:1, v/v) for 16—24 h. The sample extract was concentrated on a rotary evaporator RVO 400 SD instrument (Boeco, Germany) at 30.0 °C and 510.0 mbar until reaching $\sim$5.0 mL. The extract was then dried at ambient temperature (22.0 ± 5.0 °C) for at least seven days. Fat content was determined gravimetrically. The extract was reconstituted with $\sim$5.0 mL of hexane, gently heated in a water bath (40.0 °C) for one min and loaded in the top of the previously conditioned acid/basic column. The soxhlet round bottom flask was washed three more times with $\sim$5.0 mL of hexane and loaded in the column. When the extract was passed through the column, 180.0 mL of hexane was added to elute the analytes. The extract was collected and concentrated at 40.0 °C and 250.0 mbar until reaching $\sim$2.0 mL.

The extract obtained from the acid/basic column was loaded at the top of the nitrate column previously conditioned with 25.0 mL of hexane. The flask was washed and loaded three more times with 10.0 mL of hexane. Then, 140 mL of hexane was added (**F1**). Finally, **F1** containing PCB/PCDD/PCDF congeners was concentrated at 40.0 °C and 250.0 mbar until a reaching a volume of $\sim$1.0 mL. The florisil column was used to separate PCB from PCDD/PCDF congeners. **F1** extract was loaded into the florisil column previously conditioned with 25.0 mL of hexane. The flask was washed three more times with 10.0 mL of hexane. Then, 40.0 mL of hexane was added (**F2**). PCDD/PCDF congeners were eluted from the florisil column with 50.0 mL of hexane:acetone (1:1, v/v) (**F3**). **F2** was concentrated until reaching $\sim$1.0 mL at 40.0 °C and 250.0 mbar; and **F3** was concentrated until reaching $\sim$1.0 mL at 40.0 °C and 510 mbar at the carbon clean-up step. **F3** containing PCDD/PCDF congeners was cleaned-up with an Envi-Carb of 250.0 mg. The cartridge was conditioned with 5.0 mL of hexane. Then the extract was loaded. The flask was washed three times with 10.0 mL of hexane. Afterwards, 20.0 mL of hexane:acetone (1:1, v/v) was added. The cartridge was then turned over, and 30.0 mL of toluene were added and collected (**F4**). **F4** was concentrated until reaching $\sim$1.0 mL at 40.0 °C and 70 mbar.

Finally, **F2** (PCB) and **F4** (PCDD/PCDF) extracts were examined for fat presence and passed into chromatographic vials. The vials were concentrated to dryness with a gentle stream of nitrogen. Afterwards, **F2** and **F4** were reconstituted with 20.0 µL and 10.0 µL of isooctane respectively.

### Instrumental analysis

Analysis was done using gas chromatography coupled to mass spectrometry (GC–MS) 7890A/5975C (Agilent Technologies, USA).

The analysis conditions of PCB and PCDD/PCDF were similar, with differences in the oven gradient. A ZB-5MS plus column (60 m × 250 µm × 0.25 µm) (Phenomenex, USA) was used. Inlet temperature was 290.0 °C in pulsed splitless mode under an injection volume of 2.0 µL. Pulsed splitless mode conditions were 50.0 psi until 0.5 min, and flow rate of 60.0 mL$\cdot$min^−1^ for 2.0 min. The flow was 1.5 mL$\cdot$min^−1^. The oven conditions for PCB were initial temperature of 140.0 °C for 1.0 min, then a rate of 20.0 °C min^−1^ until reaching 200.0 °C, held for 1.0 min, after which the oven was raised to 3.0 °C min^−1^ until 280.0 °C. The oven conditions for PCDD/PCDF were initial temperature of 120.0 °C, held for 1.0 min, then a rate of 20.0 °C min^−1^ until 200 ${}^{\circ }$C, maintaining this temperature for 1.0 min. Then, the oven temperature was raised to 2.0 °C$\cdot$min^−1^ until 300.0 °C. The temperature of the transfer line was 320.0 °C. The source temperature was 280.0 °C. The mass spectrometer was operated on selected ion monitoring (SIM). Selected ions are shown in Table S1 and Table S2.

### Quality Assurance and Quality Control (QA/QC)

The method was previously validated; all details of validation and results are presented in Supplemental Material Table S1. In each analysis batch, a procedural blank using NIST${}^{{\circledR }}$ SRM${}^{{\circledR }}$ 1946 (Lake Superior fish tissue) PCB and spiked neutral silica gel were performed for QA/QC of PCDD, PCDF and PCB analysis. A batch corresponded to 20 analyzed samples. The mean percentages of PCB recovery in quality control ranged from 70.7% (PCB 169) to 92.8% (PCB 180); and for PCDD/PCDF from 60.6% (1,2,3,4,7,8-HxCDD) to 87.2% (1,2,3,4,6,7,8-HpCDD). The mean relative standard deviation in the quality control for PCB was in the range between 10.1% (PCB 114) and 22.3% (PCB 28); and for PCDD/PCDF between 7.99% (2,3,7,8-TCDF) and 25.4% (1,2,3,7,8-PeCDD). The determinant coefficient of the calibration curves was greater than 0.995 in all cases. The method quantification limits (MQL) were calculated using the t_*99*_S_*LLMV*_ method (Corley, 2002). The MQLs of PCB were in the range from 10.0 pg$\cdot$g sample^−1^ (PCB 101) to 25.5 pg$\cdot$g sample^−1^ (PCB 81); and for PCDD/PCDF from 7.70 pg$\cdot$g sample^−1^ (1,2,3,4,7,8,9-HpCDF) to 87.3 pg$\cdot$g sample^−1^ (OCDD). The acceptance criteria for the peaks were the retention time and the comparison between the ion ratios and those of the labelled compounds.

### Data handling, treatment, and statistical analysis

The toxic equivalent values (TEQ) for dl-PCB and PCDD/PCDF were calculated in pg$$\cdot$$TEQ$$\cdot$$g^−1^ w.w. The TEQs were calculated in WHO_2005_$$\cdot$$TEQ (Van Den Berg et al., 2006). TEQ_PCB_ includes 12 dl-PCB, TEQ_PCDD/PCDF_ includes 7 PCDD congeners and 10 PCDF congeners, and TEQ_PCB/PCDD/PCDF_ corresponded to the sum of TEQ_PCB_ and TEQ_PCDD/PCDF_. For statistical analysis, values in which the result was $$<$$MQL were replaced by $$\frac{1}{2}$$MQL. All statistical analysis and graphs were performed using R 4.3.1 and RStudio 2023.06.0 + 421 software. In most cases, the Kruskal–Wallis nonparametric test (KW) using correction of the Benjamini-Hochbergar method (BH) was used in the data analysis. When a KW-BH test was significant (p-value < 0.05) a post hoc Dunn test with adjusted p values by BH (Dunn-BH) was performed as a pairwise comparison method to find the differences between the pairs. Finally, the effect size was measured using epsilon squared ($${\varepsilon }^{2}$$). A large effect size corresponded to values of $${\varepsilon }^{2}$$$$\ge$$0.14. Finally, the Spearman method was considered to evaluate the correlation and relationships between the data.

## Results and discussion

PCB results are presented as indicator PCBs (PCB_6_) and dl-PCB in pg$\cdot$g^−1^ w.w and ng$\cdot$g fat^−1^. In most cases, results correspond to the sum of congeners by groups such as $\sum$PCB_6_ and $\sum$dl-PCB. In all fish samples, PCDD/PCDF congeners were below MQLs. Therefore, there is no statistical analysis of these POPs families. Furthermore, the results and tables are presented by source (Magdalena River, Cauca River, and Gulf of Urabá) and fish species (PT-MR, PT-CR, SC-MR, SC-CR, PM-MR, PM-CR, BM-GU, CU-GU, and TL-GU). In no case was the data analyzed considering the sampling period, because the differences in time between them were short (around two months) and no significant differences were found (p-value > 0.05). The results of all samples are shown in Table S3.

### Fish samples characteristics

Table 1 presents the average length (cm), weight (g), and fat content (%) by species and sources (detailed data can be consulted in Table S4). In general, fish species from rivers had a higher fat content than marine fish. The p-values by species for length, weight, and fat content were 2.62e^−13^, 3.30e^−13^ and 2.21e^−7^, respectively. The effect sizes were 0.86, 0.86, and 0.52, which indicated significant differences between these characteristics and large effect sizes.

**Table 1:** Average characteristics of analyzed fish samples

Fish specie	Length (cm) Mean (SD, Min – Max)	Weight (g) Mean (SD, Min – Max)	Fat (%) Mean (SD, Min – Max)
Magdalena River			
* P. magdaleniatum*	68.5 (4.83, 62.7 – 77.0)	2120 (601, 1430 – 3180)	3.60 (2.37, 0.524 – 7.94)
* S. cuspicaudus*	57.8 (3.88, 52.3 – 63.2)	1200 (331, 681 – 1700)	7.71 (5.41, 0.682 – 18.5)
* P. magdalenae*	32.1 (3.95, 26.6—39.1)	402 (146, 197 – 567)	1.77 (1.33, 0.232 – 4.64)
Cauca River			
* P. magdaleniatum*	93.4 (16.6, 74.0—122)	5900 (3790, 2490 – 15,200)	3.46 (2.29, 0.650 – 8.89)
* S. cuspicaudus*	60.1 (4.63, 54.0 – 70.1)	1280 (480, 680 – 2270)	5.67 (2.82, 2.73 – 11.0)
* P. magdalenae*	31.7 (1.42, 29.6 – 34.6)	408 (55.7, 340 – 499)	3.98 (1.93, 2.10 – 7.35)
Gulf of Urabá			
* B. marinus*	63.8 (6.52, 53.8 – 73.0)	2130 (675, 1200 – 3400)	1.08 (0.730, 0.391 – 2.47)
* C. undecimalis*	57.6 (7.91, 48.0 – 69.3)	1870 (926, 1000 – 3500)	1.69 (0.895, 0.317 – 3.09)
* T. lepturus*	74.9 (3.98, 71.0 – 81.7)	318 (55.9, 250 – 400)	1.29 (0.757, 0.229 – 2.68)

*Note:* Mean = Mean value, SD = standard deviation, Min = Minimum value, Max = Maximum value, n of each species by source = 10, total n = 90

The analysis of the relationship between $\sum$PCB_6_ and $\sum$dl-PCB considering length, weight, fat content, fish species and source was carried out using the Spearman correlation method. Results of parameter correlations are shown in Figure S1. Significant relationships were found between $\sum$PCB_6_ and $\sum$dl-PCB (r = 0.870***). Likewise, positive correlations between fat content and $\sum$PCB_6_ (r = 0.576***) and $\sum$dl-PCB (r = 0.536***) were found. Furthermore, significant relationships were observed between PCB levels and fish weight. It is worth noting that a significant relationship of PCB levels with fat was only found in fish species from rivers (Figure S1**)**. Therefore, there was not a significant relationship between fat content and marine fish species. The weight of the fish was associated with aging as, when the weight increased, the levels of POPs also increased, suggesting a relationship with the time of accumulation of these pollutants. However, this is not totally true, and for that reason it is strongly recommended that future research determines the age of the fish using biological methods.

### PCB levels in fish (indicator PCB and dl-PCB)

The results summary for PCB is shown in Table 2 (Magdalena River)**, **Table 3 (Cauca River) and Table 4 (Gulf of Urabá). The concentrations of $\sum$PCB_6_ were approximately ten times higher than $\sum$dl-PCB because the original PCB mixture existed of much higher amounts of PCB 138, PCB 153, and PCB 180 than other PCB congeners. Furthermore, these have a much higher persistence and longer half-life; however dl-PCB are more toxic than the indicator PCBs (Van Den Berg et al., 2006; Zhang et al., 2024). In the Magdalena River, the species with the highest levels of $\sum$PCB_6_ and $\sum$dl-PCB was *S. cuspicaudus*, followed by *P. magdaleniatum*. The species with the lowest PCB levels was *P. magdalenae* (Table 2).

**Table 2:** Levels of PCB in fish from Magdalena River

PCB	Concentration in pg/g (wet weight)		Concentration in ng/g fat	
	Mean (Median)	SD (Min—Max)	Mean (Median)	SD (Min—Max)
Magdalena River				
*P. magdaleniatum*				
PCB 28	9.77 (6.65)	8.04 (1.84—29.5)	0.555 (0.267)	0.884 (0.0232—3.02)
PCB 52	28 (18.8)	28.1 (5.58—97)	0.934 (0.786)	0.659 (0.0703—2.44)
PCB 101	41.4 (29.2)	48.7 (6.07—173)	1.25 (1.03)	0.768 (0.0765—2.57)
PCB 138	72.4 (39)	116 (6.5—396)	1.72 (1.23)	1.58 (0.266—5.89)
PCB 153	123 (63.8)	199 (12.9—678)	2.97 (2.19)	2.68 (0.359—10.1)
PCB 180	145 (94)	189 (34.3—670)	5.7 (3.16)	7.04 (0.533—24.3)
**ΣPCB**_**6**_	**419 (250)**	**582 (82.5—2040)**	**13.1 (9.27)**	**11.2 (1.33—35.8)**
PCB 77	0.677 (0.346)	0.98 (< 0.154—3.28)	0.0198 (0.0178)	0.0169 (< 0.0023—0.0488)
PCB 81	0.144 (0.148)	0.0225 (< 0.222—< 0.358)	0.00741 (0.00441)	0.00825 (< 0.0037—< 0.0566)
PCB 105	5.39 (2.46)	10.7 (< 0.108—35.5)	0.12 (0.0869)	0.16 (< 0.001634—0.527)
PCB 114	0.0515 (0.0525)	0.00788 (< 0.08—< 0.128)	0.00265 (0.00157)	0.00295 (< 0.00132—< 0.0202)
PCB 118	19.9 (9.85)	26.3 (3.13—91.6)	0.538 (0.481)	0.369 (0.0991—1.36)
PCB 123	0.0843 (0.086)	0.0129 (< 0.13—< 0.208)	0.00433 (0.00257)	0.00482 (< 0.00216—< 0.0332)
PCB 126	0.756 (0.0815)	1.43 (< 0.12—4.58)	0.0196 (0.00554)	0.0255 (< 0.001934—0.0681)
PCB 156	1.27 (0.767)	1.74 (< 0.118—5.76)	0.0301 (0.0232)	0.0336 (< 0.0029—0.115)
PCB 157	1.35 (0.435)	2.31 (< 0.112—7.49)	0.0368 (0.0215)	0.0456 (< 0.001696—0.125)
PCB 167	2.01 (0.35)	4.66 (< 0.11—15.1)	0.0413 (0.0213)	0.0682 (< 0.001656—0.224)
PCB 169	0.0599 (0.061)	0.00939 (< 0.092—< 0.148)	0.00308 (0.00183)	0.00343 (< 0.001536—< 0.0236)
PCB 189	2 (0.072)	4.6 (< 0.104—14.9)	0.043 (0.00996)	0.0697 (< 0.001698—0.222)
**ΣDL-PCB**	**33.8 (19.6)**	**50.4 (4.08—174)**	**0.866 (0.668)**	**0.683 (0.135—2.59)**
*S. cuspicaudus*				
PCB 28	60.4 (12.6)	122 (< 0.242—400)	1.76 (0.447)	3.93 (< 0.00224—12.8)
PCB 52	152 (78.2)	223 (20.3—772)	3.69 (1.19)	7.45 (0.652—24.8)
PCB 101	151 (71.1)	168 (22.6—558)	3.26 (1.79)	5.24 (0.251—17.9)
PCB 138	265 (157)	280 (20.9—803)	4.72 (2.7)	6.53 (0.489—22.6)
PCB 153	428 (255)	438 (43.1—1330)	7.39 (4.37)	8.93 (0.82—31.3)
PCB 180	351 (210)	360 (57.9—1140)	7.59 (4.52)	10.6 (0.669—36.5)
**ΣPCB**_**6**_	**1410 (788)**	**1420 (189—4550)**	**28.4 (15)**	**42.1 (3.17—146)**
PCB 77	2.1 (0.103)	5.22 (< 0.162—16.8)	0.0609 (0.00458)	0.169 (< 0.00104—0.541)
PCB 81	0.186 (0.151)	0.116 (< 0.258—< 1.028)	0.00531 (0.0027)	0.00695 (< 0.001666—< 0.0472)
PCB 105	38.1 (12.8)	55.6 (3.2—185)	0.868 (0.345)	1.79 (0.0216—5.94)
PCB 114	3.25 (0.059)	7.37 (< 0.096—22.5)	0.0825 (0.00144)	0.226 (< 0.000596—0.721)
PCB 118	78.7 (42.1)	87 (10—290)	1.66 (0.867)	2.73 (0.102—9.32)
PCB 123	0.109 (0.088)	0.0676 (< 0.152—< 0.6)	0.0031 (0.00158)	0.00406 (< 0.000974—< 0.0276)
PCB 126	0.799 (0.086)	1.5 (< 0.136—4.22)	0.0176 (0.00212)	0.0418 (< 0.001248—0.136)
PCB 156	6.99 (7.54)	7.54 (< 0.11—23.7)	0.0956 (0.0552)	0.117 (< 0.00128—0.349)
PCB 157	2.26 (0.57)	2.89 (< 0.124—7.62)	0.0404 (0.015)	0.0741 (< 0.000764—0.245)
PCB 167	7.07 (3.51)	8.16 (< 0.122—21.8)	0.121 (0.0588)	0.21 (< 0.0014—0.699)
PCB 169	0.0775 (0.0625)	0.0483 (< 0.108—< 0.428)	0.00221 (0.00112)	0.00289 (< 0.000692—< 0.01962)
PCB 189	2.69 (0.863)	3.73 (< 0.124—10.4)	0.0457 (0.014)	0.0779 (< 0.000766—0.249)
**ΣDL-PCB**	**142 (70.8)**	**167 (14.1—567)**	**3 (1.43)**	**5.38 (0.191—18.2)**
*P. magdalenae*				
PCB 28	19 (15.7)	15.6 (4.83—56.2)	1.78 (1.19)	1.74 (0.307—5.3)
PCB 52	24.1 (21.4)	15 (8.01—45.2)	2.19 (1.74)	1.8 (0.488—5.78)
PCB 101	10.4 (6.41)	8.72 (2.14—27.8)	0.894 (0.672)	0.814 (0.133—2.46)
PCB 138	12.5 (8.35)	11.9 (< 0.63—31.3)	0.985 (0.651)	1.03 (< 0.0594—3.4)
PCB 153	18.3 (8.81)	17.2 (4.17—47)	1.49 (0.834)	1.48 (0.265—4.97)
PCB 180	25 (11.4)	29 (< 0.538—87.3)	1.61 (1.71)	1.27 (< 0.0368—4.08)
**ΣPCB**_**6**_	**109 (67.3)**	**82 (26.6—238)**	**8.95 (6.68)**	**6.97 (1.69—21.1)**
PCB 77	0.359 (0.187)	0.462 (< 0.194—1.62)	0.0323 (0.0179)	0.0344 (< 0.00428—0.111)
PCB 81	0.38 (0.299)	0.226 (< 0.312—< 1.448)	0.0383 (0.0287)	0.0328 (< 0.00686—< 0.218)
PCB 105	0.993 (0.303)	1.26 (< 0.138—3.54)	0.0489 (0.0389)	0.039 (< 0.0226—0.14)
PCB 114	0.531 (0.149)	1.23 (< 0.112—4.01)	0.0325 (0.0135)	0.0569 (< 0.00244—0.191)
PCB 118	9.3 (5.71)	11.1 (< 0.522—38.8)	0.579 (0.487)	0.435 (< 0.0492—1.64)
PCB 123	0.222 (0.175)	0.132 (< 0.182—< 0.846)	0.0224 (0.0168)	0.0192 (< 0.004—< 0.1274)
PCB 126	0.278 (0.218)	0.237 (< 0.166—0.874)	0.0542 (0.015)	0.115 (< 0.00358—0.377)
PCB 156	0.303 (0.123)	0.522 (< 0.128—1.77)	0.0209 (0.0155)	0.0198 (< 0.0028—0.0624)
PCB 157	0.174 (0.137)	0.104 (< 0.142—< 0.664)	0.0176 (0.0132)	0.0151 (< 0.00314—< 0.1)
PCB 167	0.17 (0.134)	0.101 (< 0.14—< 0.648)	0.0171 (0.0128)	0.0147 (< 0.00306—< 0.0976)
PCB 169	0.158 (0.125)	0.094 (< 0.13—< 0.602)	0.0159 (0.0119)	0.0137 (< 0.00284—< 0.0906)
PCB 189	0.175 (0.138)	0.104 (< 0.144—< 0.666)	0.0176 (0.0132)	0.0151 (< 0.00316—< 0.1002)
**ΣDL-PCB**	**13 (9.59)**	**11.6 (3.95—42.6)**	**0.897 (0.713)**	**0.581 (0.372—2.36)**

**Note:** Mean = Mean value; Median = Median value; SD = Standard deviation; Min = Minimum value; Max = Maximum value, n by species = 10, total n = 90

**Table 3:** Levels of PCB in fish from Cauca River

PCB	Concentration in pg/g (wet weight)		Concentration in ng/g fat	
	Mean (Median)	SD (Min—Max)	Mean (Median)	SD (Min—Max)
**Cauca River**				
*P. magdaleniatum*				
PCB 28	13.9 (12.9)	10.3 (< 0.3—33.3)	0.61 (0.375)	0.688 (< 0.00728—2.38)
PCB 52	45.4 (26.7)	39 (< 0.212—113)	1.57 (1.19)	1.24 (< 0.00514—4.21)
PCB 101	46.4 (28.4)	40.7 (8.98—122)	1.42 (1.1)	0.884 (0.371—2.6)
PCB 138	74 (35.9)	82.6 (7.1—234)	2.2 (1.61)	1.85 (0.307—4.97)
PCB 153	118 (58.6)	133 (9.9—375)	3.59 (2.39)	3.13 (0.428—8.14)
PCB 180	132 (71.6)	155 (12.6—435)	4.25 (2.25)	3.85 (0.544—10.1)
**ΣPCB**_**6**_	**429 (235)**	**439 (61.3—1310)**	**13.6 (9.22)**	**10.9 (2.65—29.6)**
PCB 77	0.467 (0.164)	0.634 (< 0.132—1.74)	0.0187 (0.00685)	0.0206 (< 0.00202—0.057)
PCB 81	0.314 (0.131)	0.52 (< 0.2—1.78)	0.0154 (0.00399)	0.0241 (< 0.00322—0.0658)
PCB 105	8.33 (3.9)	9.87 (< 0.142—22.9)	0.201 (0.157)	0.19 (< 0.00342—0.556)
PCB 114	0.0547 (0.047)	0.0277 (< 0.072—< 0.26)	0.00338 (0.00143)	0.00588 (< 0.00115—< 0.04)
PCB 118	27.3 (11)	30.4 (3.42—85.5)	0.782 (0.603)	0.661 (0.141—2.12)
PCB 123	2.57 (0.077)	5.81 (< 0.116—17.9)	0.0757 (0.00253)	0.153 (< 0.001882—0.444)
PCB 126	0.139 (0.075)	0.185 (< 0.104—0.653)	0.00941 (0.00209)	0.0165 (< 0.001686—0.0493)
PCB 156	2.79 (1.48)	3.36 (< 0.13—9.28)	0.0716 (0.0497)	0.0714 (< 0.00318—0.192)
PCB 157	1.01 (0.26)	1.45 (< 0.096—3.93)	0.032 (0.0201)	0.0354 (< 0.001478—0.0977)
PCB 167	2.4 (0.87)	3.25 (< 0.098—9.78)	0.0991 (0.0577)	0.12 (< 0.001442—0.37)
PCB 169	0.0637 (0.055)	0.0322 (< 0.082—< 0.302)	0.00393 (0.00166)	0.00684 (< 0.001338—< 0.0464)
PCB 189	0.945 (0.0715)	1.7 (< 0.092—4.2)	0.0287 (0.00247)	0.0415 (< 0.00148—0.102)
**ΣDL-PCB**	**46.4 (21.1)**	**50.4 (6.01—146)**	**1.34 (1.12)**	**1.1 (0.257—3.64)**
*S. cuspicaudus*				
PCB 28	12.6 (9.26)	11.7 (< 0.204—34.2)	0.245 (0.225)	0.229 (< 0.00308—0.718)
PCB 52	48.2 (42.6)	19.7 (11.1—79.4)	1.07 (0.837)	0.728 (0.297—2.31)
PCB 101	52 (52.3)	24.2 (11.3—98)	1.1 (0.97)	0.641 (0.246—2.11)
PCB 138	73.9 (67.1)	41.4 (22.3—172)	1.5 (1.55)	0.742 (0.261—2.47)
PCB 153	121 (102)	83.8 (34.2—336)	2.43 (2.37)	1.33 (0.421—4.47)
PCB 180	105 (102)	77.1 (16.1—287)	2.51 (1.68)	2.48 (0.351—8.07)
**ΣPCB**_**6**_	**414 (399)**	**170 (103—709)**	**8.85 (7.7)**	**5.37 (2.76—18.5)**
PCB 77	0.461 (0.0945)	0.505 (< 0.124—1.24)	0.0109 (0.00275)	0.0149 (< 0.00146—0.0456)
PCB 81	0.364 (0.134)	0.738 (< 0.2—2.46)	0.00713 (0.00293)	0.0135 (< 0.00234—0.0454)
PCB 105	8.69 (9.43)	5.98 (< 0.114—19.9)	0.19 (0.2)	0.132 (< 0.001034—0.441)
PCB 114	0.0475 (0.048)	0.00613 (< 0.072—< 0.108)	0.00103 (0.000975)	0.000483 (< 0.000836—< 0.00356)
PCB 118	28.7 (29)	12.9 (8.86—46.6)	0.593 (0.57)	0.327 (0.121—1.31)
PCB 123	1.75 (0.0835)	3.79 (< 0.116—11.4)	0.0358 (0.00175)	0.0735 (< 0.001366—0.203)
PCB 126	0.474 (0.0695)	0.863 (< 0.104—2.34)	0.0121 (0.00143)	0.0231 (< 0.001224—0.0658)
PCB 156	2.96 (2.34)	3.14 (< 0.09—9.08)	0.084 (0.0383)	0.11 (< 0.001384—0.333)
PCB 157	1.4 (0.773)	1.99 (< 0.1—6.43)	0.0379 (0.0146)	0.0564 (< 0.001074—0.181)
PCB 167	2.44 (1.9)	2.4 (< 0.09—8.54)	0.0483 (0.0429)	0.0381 (< 0.00238—0.11)
PCB 169	0.055 (0.055)	0.00742 (< 0.082—< 0.126)	0.00119 (0.00113)	0.000562 (< 0.000972—< 0.00414)
PCB 189	3.55 (0.721)	8.37 (< 0.092—27.2)	0.0685 (0.0194)	0.134 (< 0.001076—0.438)
**ΣDL-PCB**	**50.9 (50.2)**	**21.5 (17—80.6)**	**1.09 (1.02)**	**0.616 (0.163—2.27)**
*P. magdalenae*				
PCB 28	17.1 (13.1)	13.3 (1.62—37.7)	0.43 (0.43)	0.354 (0.0665—1.31)
PCB 52	39.1 (32.8)	24.2 (10—74.2)	1.04 (0.872)	0.724 (0.41—2.58)
PCB 101	11.2 (8.87)	6.57 (4.52—24.5)	0.284 (0.267)	0.0951 (0.164—0.426)
PCB 138	8.25 (8.2)	4.63 (2.82—18.4)	0.215 (0.189)	0.087 (0.102—0.404)
PCB 153	11.8 (11.1)	5.78 (4.46—23)	0.318 (0.305)	0.129 (0.162—0.574)
PCB 180	16.3 (11.4)	15 (0.076—48.3)	0.393 (0.441)	0.21 (0.00157—0.658)
**ΣPCB**_**6**_	**104 (87.1)**	**58.8 (40.8—196)**	**2.68 (2.56)**	**1.15 (1.49—5.11)**
PCB 77	0.576 (0.163)	0.693 (< 0.168—2.21)	0.0222 (0.00355)	0.0326 (< 0.00352—0.105)
PCB 81	0.179 (0.169)	0.0542 (< 0.258—< 0.632)	0.00501 (0.0047)	0.00149 (< 0.00562—< 0.015)
PCB 105	0.751 (0.075)	1.45 (< 0.114—4.04)	0.0132 (0.00271)	0.0229 (< 0.0033—0.0629)
PCB 114	0.0638 (0.06)	0.0194 (< 0.092—< 0.226)	0.00179 (0.00168)	0.000534 (< 0.00202—< 0.00536)
PCB 118	13 (4.75)	15.7 (2.19—42.1)	0.256 (0.149)	0.204 (0.0793—0.656)
PCB 123	0.105 (0.0985)	0.0317 (< 0.152—< 0.37)	0.00293 (0.00275)	0.000873 (< 0.00328—< 0.00876)
PCB 126	0.0938 (0.0885)	0.0284 (< 0.136—< 0.332)	0.00262 (0.00246)	0.000782 (< 0.00294—< 0.00786)
PCB 156	0.0734 (0.0695)	0.0223 (< 0.106—< 0.26)	0.00205 (0.00193)	0.000612 (< 0.0023—< 0.00614)
PCB 157	0.56 (0.083)	1.5 (< 0.124—4.84)	0.0189 (0.00252)	0.0526 (< 0.00258—0.169)
PCB 167	0.446 (0.081)	1.15 (< 0.116—3.72)	0.0155 (0.00237)	0.042 (< 0.00252—0.135)
PCB 169	0.0744 (0.0705)	0.0226 (< 0.108—< 0.264)	0.00208 (0.00196)	0.000621 (< 0.00234—< 0.00624)
PCB 189	0.0821 (0.0775)	0.0249 (< 0.118—< 0.29)	0.0023 (0.00216)	0.000687 (< 0.00258—< 0.0069)
**ΣDL-PCB**	**16 (7.94)**	**16.1 (4.48—47.1)**	**0.345 (0.296)**	**0.197 (0.141—0.734)**

**Note:** Mean = Mean value; Median = Median value; SD = Standard deviation; Min = Minimum value; Max = Maximum value, n by species = 10, total n = 90

**Table 4:** Levels of PCB in fish from Gulf of Uraba

PCB	Concentration in pg/g (wet weight)		Concentration in ng/g fat	
	Mean (Median)	SD (Min—Max)	Mean (Median)	SD (Min—Max)
**Gulf of Uraba**				
*B. marinus*				
PCB 28	7.93 (5.46)	9.78 (1.45—35.1)	1.24 (0.544)	2.31 (0.0822—7.79)
PCB 52	6.99 (4.84)	5.86 (1.94—17.7)	0.868 (0.568)	1.1 (0.122—3.94)
PCB 101	9.07 (7.27)	8.71 (2.8—32)	0.893 (0.901)	0.455 (0.141—1.72)
PCB 138	20.3 (14.1)	20.4 (4.95—60.2)	1.95 (1.73)	1.26 (0.303—4.14)
PCB 153	33 (27.1)	27.9 (9.29—94.5)	3.49 (2.97)	2.27 (0.505—7.72)
PCB 180	78.5 (50.3)	95 (17.6—340)	7.38 (7.44)	4.03 (0.772—13.7)
**ΣPCB**_**6**_	**156 (115)**	**147 (43.8—529)**	**15.8 (15.2)**	**8.14 (1.92—27.6)**
PCB 77	0.282 (0.0995)	0.423 (< 0.132—1.42)	0.0486 (0.0142)	0.0954 (< 0.0054—0.316)
PCB 81	0.138 (0.148)	0.0284 (< 0.192—< 0.346)	0.0183 (0.0169)	0.0111 (< 0.00864—< 0.0772)
PCB 105	3.57 (1.36)	7.14 (0.339—23.8)	0.263 (0.171)	0.279 (0.0344—0.959)
PCB 114	4.92 (0.054)	15.4 (< 0.068—48.7)	0.203 (0.00688)	0.619 (< 0.00334—1.97)
PCB 118	10.4 (4.64)	17.6 (1.29—59.6)	0.824 (0.617)	0.685 (0.0974—2.4)
PCB 123	0.0803 (0.086)	0.0166 (< 0.112—< 0.202)	0.0107 (0.0099)	0.00651 (< 0.00506—< 0.0452)
PCB 126	0.0878 (0.079)	0.0465 (< 0.1—0.214)	0.0103 (0.00941)	0.00527 (< 0.00452—0.0202)
PCB 156	1.81 (1.88)	1.34 (< 0.078—3.66)	0.245 (0.204)	0.242 (< 0.00916—0.785)
PCB 157	0.0916 (0.069)	0.0857 (< 0.088—0.333)	0.00964 (0.00884)	0.00493 (< 0.00396—0.0177)
PCB 167	0.451 (0.468)	0.32 (< 0.086—0.978)	0.051 (0.0418)	0.0463 (< 0.01142—0.16)
PCB 169	0.0574 (0.0615)	0.0118 (< 0.08—< 0.144)	0.0076 (0.00704)	0.00463 (< 0.0036—< 0.0322)
PCB 189	0.486 (0.0775)	0.8 (< 0.088—2.48)	0.0585 (0.0111)	0.0966 (< 0.00398—0.305)
**ΣDL-PCB**	**22.4 (10.5)**	**40.5 (3.09—137)**	**1.75 (1.39)**	**1.57 (0.232—5.52)**
*C. undecimalis*				
PCB 28	13.5 (11.5)	8.83 (4.21—33.1)	1.45 (0.568)	1.8 (0.171—5.79)
PCB 52	54.9 (21.8)	70.5 (7.71—228)	5.78 (1.2)	6.9 (0.311—16.9)
PCB 101	61.1 (23.6)	69.4 (7.75—183)	7.25 (1.29)	9.96 (0.335—29.9)
PCB 138	63.5 (42.5)	62.7 (7.06—168)	6.8 (2.49)	8.11 (0.412—22.3)
PCB 153	93.2 (59.6)	85.9 (11.5—234)	10.5 (3.97)	13.8 (0.672—42.4)
PCB 180	138 (61.1)	180 (19.4—620)	18.6 (4.83)	36.9 (1.13—121)
**ΣPCB**_**6**_	**424 (232)**	**422 (58.1—1210)**	**50.3 (14.4)**	**73.5 (3.39—236)**
PCB 77	0.64 (0.108)	0.806 (< 0.172—2.09)	0.0449 (0.0242)	0.0631 (< 0.00668—0.206)
PCB 81	2.06 (0.168)	4.75 (< 0.22—15.2)	0.121 (0.022)	0.279 (< 0.00978—0.904)
PCB 105	20.8 (9.04)	25.7 (0.665—78.7)	2.37 (0.505)	2.98 (0.044—7.37)
PCB 114	1.28 (0.0625)	2.24 (< 0.098—6.9)	0.121 (0.00362)	0.155 (< 0.0033—0.344)
PCB 118	47.4 (20.7)	59.2 (3.43—181)	5.14 (1.14)	6.22 (0.167—15.4)
PCB 123	0.0875 (0.084)	0.0123 (< 0.128—< 0.206)	0.0085 (0.00486)	0.00789 (< 0.0054—< 0.0514)
PCB 126	0.747 (0.0915)	1.09 (< 0.116—3.25)	0.0976 (0.0146)	0.199 (< 0.00484—0.636)
PCB 156	10.4 (2.74)	13 (< 0.118—30.4)	1.38 (0.138)	1.98 (< 0.00378—5.76)
PCB 157	2.49 (0.657)	3.31 (< 0.126—8.38)	0.287 (0.0583)	0.435 (< 0.00424—1.35)
PCB 167	2.07 (1.14)	2.2 (< 0.124—5.76)	0.24 (0.0605)	0.304 (< 0.0048—0.806)
PCB 169	0.0623 (0.0595)	0.00867 (< 0.092—< 0.146)	0.00605 (0.00346)	0.00561 (< 0.00384—< 0.0366)
PCB 189	1.84 (0.073)	4.51 (< 0.102—14.5)	0.318 (0.00454)	0.887 (< 0.00424—2.83)
**ΣDL-PCB**	**89.9 (48.6)**	**100 (6.78—287)**	**10.1 (3.16)**	**12.5 (0.345—33.9)**
*T. lepturus*				
PCB 28	16.5 (11.7)	9.83 (8.16—35.9)	1.66 (1.23)	1.19 (0.689—4.52)
PCB 52	27.9 (26)	16.8 (10.7—65.6)	2.97 (1.99)	3.02 (1.03—11.3)
PCB 101	13.9 (10.3)	14.8 (4.25—55)	1.27 (0.797)	1.05 (0.469—3.78)
PCB 138	6.67 (6.77)	4.74 (< 0.238—14.8)	0.552 (0.592)	0.362 (< 0.0416—1.13)
PCB 153	12.6 (12.4)	8.6 (< 0.252—25.5)	1.01 (1.05)	0.629 (< 0.0438—2.1)
PCB 180	19.2 (18.4)	12.2 (< 0.292—36.5)	1.52 (1.64)	0.729 (< 0.0714—2.41)
**ΣPCB**_**6**_	**96.8 (93.4)**	**45.4 (45.6—163)**	**8.97 (7.12)**	**4.23 (6.11—19.9)**
PCB 77	0.347 (0.242)	0.345 (< 0.228—1.25)	0.034 (0.0284)	0.025 (< 0.01224—0.0728)
PCB 81	0.401 (0.387)	0.214 (< 0.366—< 1.7)	0.0454 (0.0345)	0.0342 (< 0.01962—< 0.212)
PCB 105	0.432 (0.206)	0.498 (< 0.162—1.43)	0.0444 (0.0287)	0.0543 (< 0.00868—0.188)
PCB 114	0.143 (0.139)	0.0766 (< 0.13—< 0.608)	0.0162 (0.0123)	0.0122 (< 0.007—< 0.076)
PCB 118	7.07 (3.6)	10.5 (2.2—36.8)	0.788 (0.32)	0.981 (0.156—2.68)
PCB 123	0.234 (0.227)	0.125 (< 0.214—< 0.994)	0.0265 (0.0202)	0.02 (< 0.01146—< 0.1242)
PCB 126	0.441 (0.232)	0.73 (< 0.192—2.49)	0.0805 (0.0181)	0.187 (< 0.01026—0.612)
PCB 156	0.324 (0.182)	0.49 (< 0.15—1.7)	0.0882 (0.0142)	0.229 (< 0.00804—0.74)
PCB 157	0.6 (0.185)	1.32 (< 0.168—4.36)	0.0678 (0.0158)	0.15 (< 0.009—0.492)
PCB 167	0.179 (0.174)	0.0961 (< 0.164—< 0.762)	0.0203 (0.0155)	0.0153 (< 0.00878—< 0.0952)
PCB 169	0.166 (0.161)	0.089 (< 0.152—< 0.706)	0.0189 (0.0144)	0.0142 (< 0.00816—< 0.0884)
PCB 189	0.184 (0.178)	0.0986 (< 0.168—< 0.782)	0.0209 (0.0159)	0.0157 (< 0.00902—< 0.0976)
**ΣDL-PCB**	**10.5 (7.45)**	**10.6 (4.55—40.3)**	**1.25 (0.71)**	**1.27 (0.243—3.99)**

**Note:** Mean = Mean value; Median = Median value; SD = Standard deviation; Min = Minimum value; Max = Maximum value, n by species = 10, total n = 90

In contrast to the Magdalena River, the species with the highest levels of $\sum$PCB_6_ in the Cauca River was *P. magdaleniatum,* followed by *S. cuspicaudus*. Regarding $\sum$dl-PCB, *S. cuspicaudus* had the highest levels. Also, *P. magdalenae* was the species with the lowest PCB levels in the Cauca River (Table 3).

Marine species in general had lower levels than river species. The species with the highest concentrations were *C. undecimalis* and *B. marinus*. Finally, the species *T. lepturus* had the lowest PCB levels in the marine species.

The concentrations of $\sum$PCB_6_ were in the range between 26.6 pg$\cdot$g^−1^ w.w and 4550 pg$\cdot$g^−1^ w.w, and the mean and median values were 395.0 and 186.0 pg$\cdot$g^−1^ w.w respectively. These concentrations were lower than those reported in other studies. For example, seven indicator PCB (including PCB 118) ranged from 300.0 pg$\cdot$g^−1^ w.w to 3.10$\times$10^6^ pg$\cdot$g^−1^ w.w (mean value 3.98$\times$10^5^ pg$\cdot$g^−1^ w.w) in marine fish from four areas of China (Liu et al., 2011). In addition, Tran-Lam et al. (2023) reported a mean dl-PCB in marine fish muscle of 42.5 ng$\cdot$g^−1^ fat, which is 20 times higher than the findings of this study (2.30 ng$\cdot$g^−1^ fat). Likewise, some reports from the United States revealed the presence of PCB 118 in fish (Li et al., 2022; McKelvey et al., 2010) with a higher median than that obtained in this work for this congener (26.9 pg$\cdot$g^−1^ w.w). Additionally, a study in fish from the southern Baltic Sea found mean values of $\sum$PCB_6_ and $\sum$dl-PCB in herring, sprat and salmon between 226 ng$\cdot$g^−1^ fat and 1090 ng$\cdot$g^−1^ fat; and between 51.3 ng$\cdot$g^−1^ fat and 247.3 ng$\cdot$g^−1^ fat during the 2002–2006 period (Szlinder-Richert et al., 2009).

All these reports indicate that the PCB presence in fish samples was higher than those found in this study. It is well known that PCB concentrations are higher in industrialized, POP-manufacturing countries, and countries of the northern hemisphere in comparison to those from non-manufacturing countries, in developing, warm and/or tropical countries (Hageman et al., 2015; Harrad, 2009). However, when the results of the present study are compared with other studies from tropical countries in the region, the PCB values are similar and in some cases in the same magnitude order. A study from Brazil found PCB in five of twelve analyzed species of fish in the range between 0.51 ng$\cdot$g^−1^ fat and 26.1 ng$\cdot$g^−1^ fat (Santos et al., 2020). Another study reported concentrations ranging from 2290.0 pg$\cdot$g^−1^ w.w to 27600.0 pg$\cdot$g^−1^ w.w in muscle of the three species (Lavandier et al., 2013). Furthermore, De Azevedo E Silva et al. (2007) found a mean concentration of $\sum$PCB_6_ of 3150.0 pg$\cdot$g^−1^ w.w and 6500 pg$\cdot$g^−1^ w.w in fish species from the Brazilian coast.

Furthermore, to the best of our knowledge, only one study on the content of PCB in fish from Colombia has been reported. Unfortunately, the study did not discriminate between the evaluated congeners and the results were expressed as total PCB in the fish muscle. The studied species were similar to those in this study, and the Magdalena River was one of the sample sources. PCB levels ranged from 5.0 ng$\cdot$g^−1^ fat to 5600.0 ng$\cdot$g^−1^ fat and the total number of analyzed samples was 32 (Cala & Södergren, 1999). Comparing the results, it could be assumed that $\sum$PCB_6_ is around 50.0% of the total PCB in fish muscle. The range of total PCB levels from the Magdalena River was between 1.46 ng$\cdot$g^−1^ fat to 164.3 ng$\cdot$g^−1^ fat (n = 30) with a mean of 18.4 ng$\cdot$g^−1^ fat. It is important to note that these results, in some cases, have the same magnitude order. However, the results suggest a decrease in PCB levels, which is consistent with the evidence of a reduction in PCB presence around the world (Fiedler et al., 2023; Hites & Holsen, 2019; Nyberg et al., 2015). All the findings indicated that the presence of PCB in fish from the Antioquia region (Colombia) is relatively low in comparison with reports around the world, including tropical regions.

**Fig. 2:**
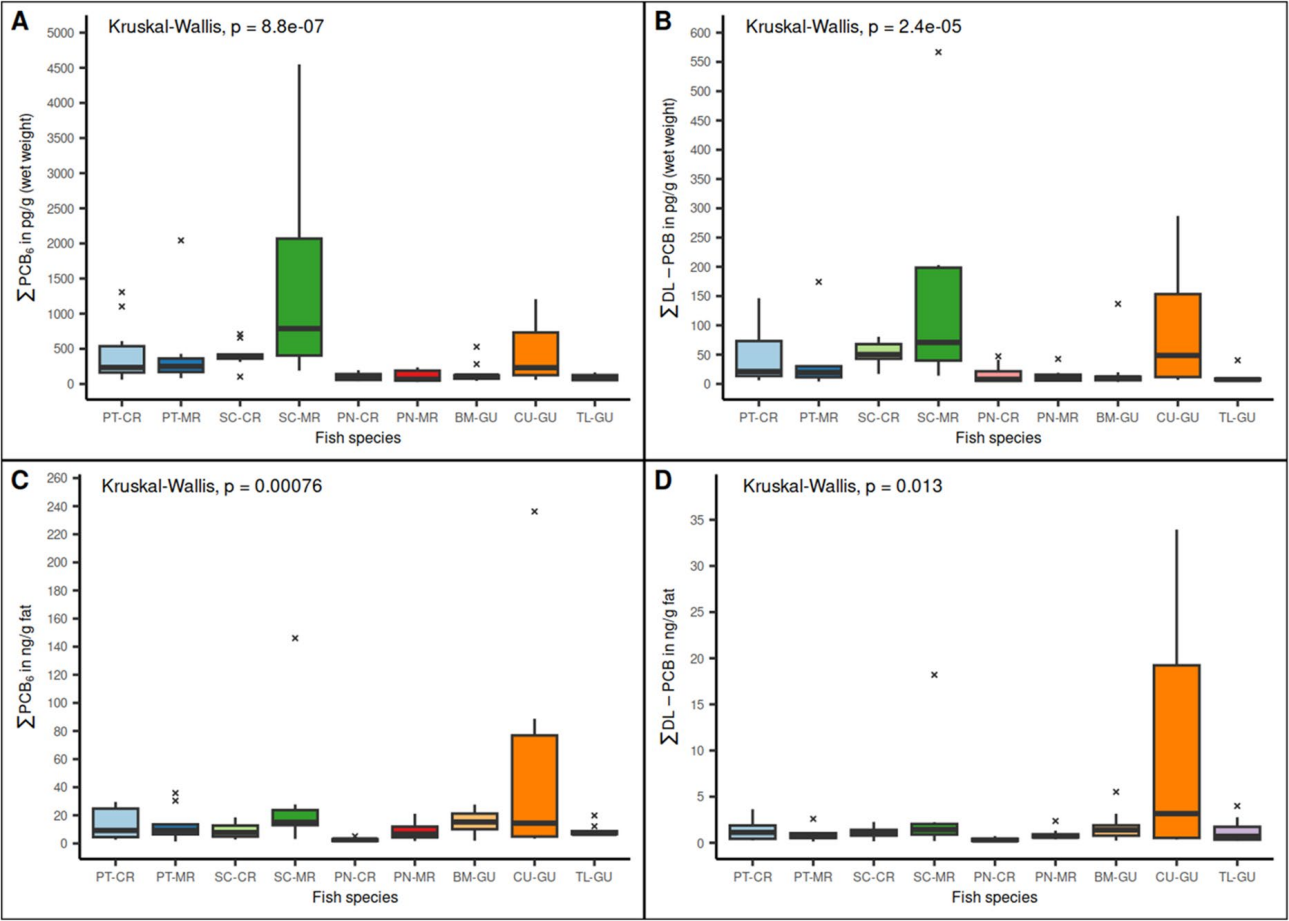
Boxplot of indicators PCB and dl-PCB by fish species. **A**: $\sum {\text{PCB}}_{6}$ (PCB 28, PCB 52, PCB 101, PCB 138, PCB 153, and PCB 180) in $\text{pg}\cdot{\text{g}}^{-1}$ w.w; **B**: $\sum \text{dl}-{\text{PCB}}_{ }$ (PCB 77, PCB 81, PCB 105, PCB 114, PCB 118, PCB 123, PCB 126, PCB 156, PCB 157, PCB 167, PCB 169, and PCB 189) in $\text{pg}\cdot{\text{g}}^{-1}$ w.w; **C**: $\sum {\text{PCB}}_{6}$ in $\text{ng}\cdot {\text{g}}^{-1} \text{fat}$; **D**: $\sum \text{dl}-{\text{PCB}}_{ }$ in $\text{ng}\cdot {\text{g}}^{-1}\text{fat}$. **PT-CR**: *P. magdaleniatum*-Cauca River; **PT-MR**: *P. magdaleniatum*-Magdalena River; **SC-CR**: *S. cuspicaudus*-Cauca River; **SC-MR**: *S. cuspicaudus*-Magdalena River; **PM-CR**: *P. magdalenae*-Cauca River; **PM-MR**: *P. magdalenae*-Magdalena River; **BM-GU**: *B. marinus*-Gulf of Uraba; **CU-GU**: *C. undecimalis*-Gulf of Uraba; **TL-GU**: *T. lepturus*-Gulf of Uraba; **Kruskal–Wallis**: non-parametric test, p-value < 0.05 indicates significant differences

The PCB congeners with the highest values in the river’s species were PCB 180 and PCB 153. PCB 180 was the congener with the highest mean concentration in *P. magdaleniatum* and *P. magdalenae* species in both rivers. In contrast with the above, the species *S. cuspicaudus* had higher levels of PCB 153 than PCB 180. In the marine species, the congeners with the highest levels were PCB 180, PCB 153, and PCB 138. Regarding dl-PCB congeners, the highest concentrations in descending order were PCB 118, PCB 105, PCB 156, and PCB 167. The species with the highest concentrations of PCB 118, PCB 105 and PCB 167 was *S. cuspicaudus* (Magdalena River). For PCB 156, the highest concentration was found in the species *C. undecimalis* (Gulf of Urabá). In Fig. 2 the boxplots of the results for the indicators PCB and dl-PCB levels by fish species are shown.

Significant differences were found (p-value < 0.05) by fish species. The main findings of significant differences between pairs were in fish species with relative higher and lower levels. In this sense, differences were found between, one on hand, *S. cuspicaudus* and *P. magdaleniatum* (species with relative high levels of PCB) from both rivers, and on the other, *P. magdalenae* and *T. lepturus* (species with relative low levels of PCB). Similarly, *S. cuspicaudus* from both rivers had significant differences with *B. marinus* (Gulf of Urabá), while no differences were found between *P. magdalenae* and *T. lepturus* species. Furthermore, there were no significant differences between *S. cuspicaudus* and *P. magdaleniatum* species, and *C. undecimalis* with *B. marinus*. In all cases the ${\varepsilon }^{2}$ values were higher than 0.14, indicating large effect sizes.

As mentioned above, the species from the Magdalena and Cauca rivers with higher fat content had higher levels of PCB, in contrast to those from the Gulf of Urabá (marine species). This fact explains why the high dispersion of PCB levels in *S. cuspicaudus* from the Magdalena River was significantly reduced when the data were normalized for fat content (Fig. 2). Furthermore, dl-PCB levels were influenced by the fat content of fish from both rivers, but not by that of marine fish species. This could be explained considering that non-ortho and mono-ortho PCB have higher octanol–water partition coefficients (Log *K*_*oW*_) than homologs with greater ortho substitutions, suggesting the potential to accumulate coplanar congeners due to the higher affinity for fat (Willman et al., 1997). The above does not apply to marine species, where a reduced dispersion and size of the boxplot in function of the fat content were not observed, suggesting that these species are less dependent on fat to accumulate pollutants or that the accumulation does not spread across the food chain.

**Fig. 3:**
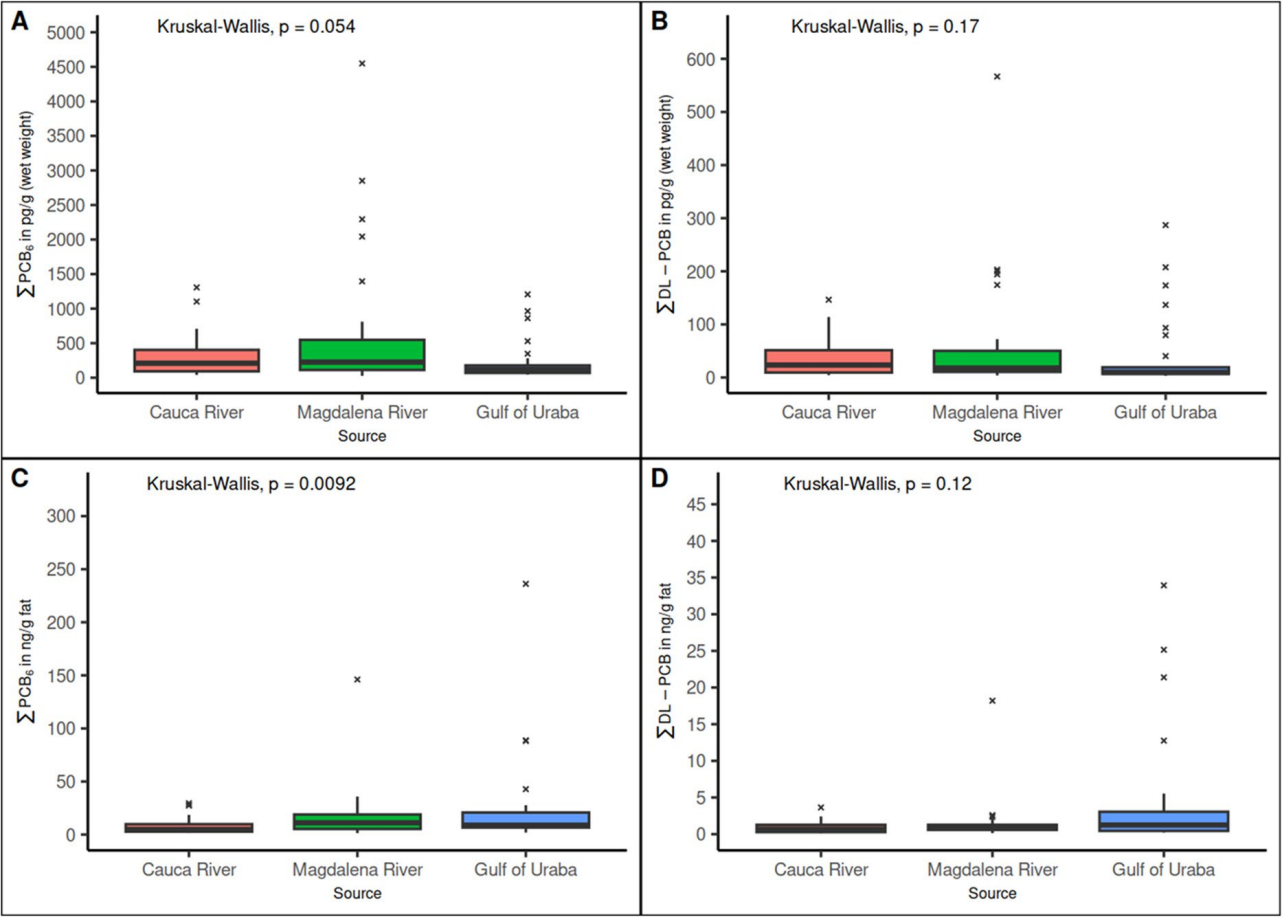
Boxplot of indicator PCB and dl-PCB in fish by source. **A:** ∑PCB_6_ (PCB 28, PCB 52, PCB 101, PCB 138, PCB 153, and PCB 180) in pg⋅g^−1^ (wet weight); **B:** ∑dl-PCB (PCB 77, PCB 81, PCB 105, PCB 114, PCB 118, PCB 123, PCB 126, PCB 156, PCB 157, PCB 167, PCB 169, and PCB 189) in pg⋅g^−1^ (wet weight); **C:** ∑PCB_6_ in ng⋅g^−1^ fat; **D:** ∑dl-PCB in ng⋅g^−1^ fat; **Kruskal–Wallis:** non-parametric test, p-value < 0.05 indicates there is significant differences

To evaluate the influence of the origin, the PCB concentrations of species from the same source were summed (Fig. 3). Only a significant difference between sources and indicator PCB in ng$\cdot$g fat^−1^ units was observed (p-value = 0.0092). This result suggests that the fat content influences the distribution of PCB level rather than the source. The Dunn-BH test showed differences between the Cauca River with the Magdalena River and the Gulf of Urabá.

The p-values between pairs were as follows: the Cauca River with the Magdalena River (p-value = 0.0148); and the Cauca River with the Gulf of Urabá (p-value = 0.0059). There were no differences between the Magdalena River and the Gulf of Urabá (p-value = 0.29). Finally, the ${\varepsilon }^{2}$ value was 0.11, indicating a medium effect size.

Significant differences between PCB levels in function with the fish species has been reported by other authors previously (Consales et al., 2023; Serrano et al., 2008; Tran-Lam et al., 2023; van Leeuwen et al., 2009). However, the differences could be associated with the origin and characteristics of the fish and its diet. In this case, the differences in the fish species are not associated with its origin, since the PCB levels were not significant when the source was considered (p-values were 0.054 for the indicator PCB and 0.17 for dl-PCB). Significant differences were found when the data were normalized according to the fat content. Indeed, this was confirmed with the Spearman correlation. In summary, the species of both rivers are highly influenced by their fat content. However, in marine species PCB levels are independent of this parameter. Therefore, significant differences were found between the pollutant levels in fish from the Gulf of Urabá and those from the Magdalena and Cauca rivers. According to the above, the influence of the sample origin on the differences in PCB levels is minimal. Differences could be associated with the characteristics of the species and its diet. Predatory species at a higher level of the chain food, such as *P. magdaleniatum* and *S. cuspicaudus,* had higher levels than *P. magdalenae,* as has been reported by other researches (Masset et al., 2019).

**Fig. 4:**
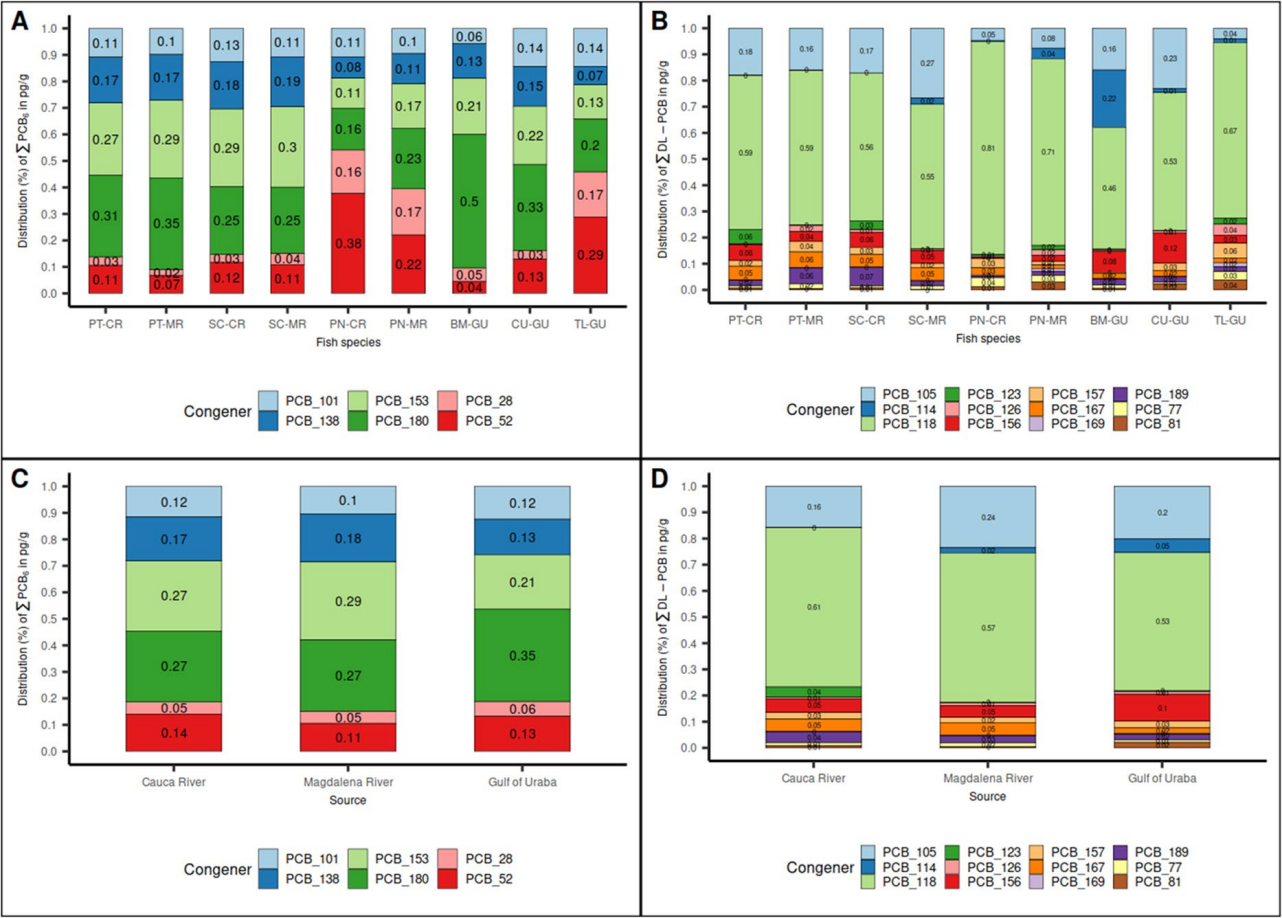
Distribution of PCB congeners by fish species and source. Proportion of the PCB indicator (PCB 28, PCB 52, PCB 101, PCB 138, PCB 153, and PCB 180) in $pg\cdot {g}^{-1}$ (wet weight) by fish species; **B**: Proportion of dl-PCB (PCB 77, PCB 81, PCB 105, PCB 114, PCB 118, PCB 123, PCB 126, PCB 156, PCB 157, PCB 167, PCB 169, and PCB 189) in $pg\cdot {g}^{-1}$ (wet weight) by fish species; **C**: Proportion of indicator PCB in $pg\cdot {g}^{-1}$ (wet weight) by source; **D**: Proportion of dl-PCB in $pg\cdot {g}^{-1}$ (wet weight) by source. **PT-CR**: *P. magdaleniatum*-Cauca River; **PT-MR**: *P. magdaleniatum*-Magdalena River; **SC-CR**: *S. cuspicaudus*-Cauca River; **SC-MR**: *S. cuspicaudus*-Magdalena River; **PM-CR**: *P. magdalenae*-Cauca River; **PM-MR**: *P. magdalenae*-Magdalena River; **BM-GU**: *B. marinus*-Gulf of Uraba; **CU-GU**: *C. undecimalis*-Gulf of Uraba; **TL-GU**: *T. lepturus*-Gulf of Uraba

The distribution of PCB congeners was evaluated by plotting the sum of each congener in proportion to the total amount (Fig. 4). The patterns between the homologous species of both rivers were very similar, with slight differences. Regarding the indicator PCB, the species *P. magdaleniatum* had the highest proportion of PCB 180, followed by PCB 153 and PCB 138. *S. cuspicaudus* presented the highest proportion of PCB 153. *P. magdalenae* and *T. lepturus* had the highest proportions of PCB 52 and PCB 28. Furthermore, *C. undecimalis* (Gulf of Urabá) had a distribution pattern very close that of *P. magdaleniatum*. Finally, *B. marinus* showed a different pattern, with the highest proportion of PCB 180 with respect to other species.

Regarding dl-PCB, the PCB 118 congener was the most abundant and showed the highest proportions of all the dl-PCB. The second most abundant congener was PCB 105, except for *B. marinus,* where PCB 114 was higher. The third proportion of congeners varied depending on the fish species. Finally, the species *P. magdalenae* and *T. lepturus* seemed to have patterns. However, as these species had low values and low detection percentages, many results were replaced by $\frac{1}{2}$MQL.

Regarding the PCB patterns by source, results of the indicator PCB and dl-PCB patterns were very similar, with slight differences associated with the sample sources. PCB 180 was slightly higher in the Gulf of Urabá in comparison to the rivers. Likewise, PCB 153 was slightly higher in the Magdalena River compared to the Gulf of Urabá. Regarding dl-PCB, the proportions of PCB 118 and PCB 105 are practically the same in all sources. In the Gulf of Urabá a higher presence of PCB 156 and PCB 114 was noted. In addition, PCB 123 presence was higher in the Cauca River.

The species at higher levels of the food chain presented higher levels of highly chlorinated PCB (PCB 180/PCB 153/PCB 138), which are more lipophilic, and their presence could be explained by the biomagnification process throughout the food chain (Reddy et al., 2019; Willman et al., 1997). In species at low levels in the food chain (*P. magdalenae* and *T. lepturus)* higher proportions of tri- and tetra-chlorinated PCB (PCB 52/PCB 28) were found, which are less lipophilic and more soluble in water. For example, *P. magdalenae* feeds by consuming detritus (decomposed organic matter) using its mouth to remove mud from the bottom to access this detritus. In this sense, the PCB content in these species could be associated more to its presence in sediment and water than to the consumption of other species of fish (Willman et al., 1997). Furthermore, regarding the proportions of the indicator PCB, as explained above, the most abundant congener was PCB 180 (hepta chlorinated) in the *P. magdaleniatum, B. marinus,* and *C. undecimalis* species, in proportions between 31.0% and 50.0%. These results differed from those previously reported in other places where PCB 153 and PCB 138 (hexa chlorinated) were the most abundant congeners (Consales et al., 2023; Liu et al., 2011). These finding suggests a specific pattern for the fish from the evaluated region which could be associated mainly to the background levels of Aroclor 1260, which contains in proportion a slightly higher content of hepta-chlorine PCB congeners than hexa-chlorine PCB (Frame et al., 1996). The background level could be a result of the fact that Colombia has never been a PCB manufacturer and imported these pollutants mainly through electric transformers (Avila et al., 2022a, 2022b, 2022c, 2022d). However, the results obtained in this study could vary according to the fish species, the geographic region and country.

These results agree with the reports of other researchers in which the most abundant congener was PCB 118, followed by PCB 105 (Mezzetta et al., 2011; Piskorska-Pliszczynska et al., 2012; Rawn et al., 2006; Suominen et al., 2011; Szlinder-Richert et al., 2009). The species *B. marinus* showed a special affinity for PCB 114, which is a penta-chlorinated PCB equal to PCB 118. *B. marinus* metabolism showed a preference or competency mechanism by accumulating PCB 114 (22.0%) instead of PCB 118, in contrast to the other species. This could be confirmed considering that the proportion of PCB 118 was reduced by 10.0 – 15.0% compared to the other species, but the sum of the proportions of these congeners (PCB 114/PCB 118) corresponded to 68.0%, which is similar to the proportion of PCB 118 in the other species.

Finally, the TEQ values of dl-PCB were calculated using the toxic equivalency factor (TEF) values WHO_2005_ (Van Den Berg et al., 2006). Most TEQ_PCB_ values were below 0.050 pg$\cdot$TEQ_PCB_$\cdot$g^−1^ w.w. Of all the samples, 15.6% (n = 14) had values between 0.050 pg$\cdot$TEQ_PCB_$\cdot$g^−1^ w.w and 0.50 pg$\cdot$TEQ_PCB_$\cdot$g^−1^ w.w. The two highest values were 0.46 pg$\cdot$TEQ_PCB_$\cdot$g^−1^ w.w (*P. magdaleniatum)* and 0.44 pg$\cdot$TEQ_PCB_$\cdot$g^−1^ w.w (*S. cuspicaudus)* (source Magdalena River). Other species with relatively higher values than 0.050 pg$\cdot$TEQ_PCB_$\cdot$g^−1^ w.w were *C. undecimalis* and *T. lepturus,* both from the Gulf of Urabá (3.0 pg$\cdot$TEQ_PCB_$\cdot$g^−1^ w.w). All calculated TEQ_PCB_ are in **Supplementary Material.**

Additionally, none of the analyzed samples exceeded the admissible level for fish for consumption proposed by the European Commission (75.0 ng$\cdot$g^−1^ w.w of $\sum$PCB_6_) (EU, 2023). In the same way, all results were below 8.0 pg$\cdot$TEQ$\cdot$g^−1^, the maximum w.w level proposed by Colombia (MINSALUD, 2012). Hence, the highest TEQ_PCB_ values were around 16 times lower than the regulated level. These results suggest low levels of PCB in the region, as has been reported previously (Avila et al., 2022a; Avila, Mendoza, et al., 2022a, 2022b, 2022c, 2022d).

Finally, PCB levels could be associated with the background levels of these pollutants in the region as a result of the ubiquitous presence of these compounds. Furthermore, no influence by origin was found and the concentrations of these chemical compounds are more dependent on the characteristics (fat content), diet, habits, and metabolism of the fish species. In this way, occasional hot spots of release or contamination from the origin of the fish were not related to the results. Furthermore, a baseline concentration of PCB was established to evaluate the trend of these pollutants in the region in future studies.

In summary, the levels of PCB and PCDD/PCDF in fish from the Antioquia region (Colombia) were low compared to reports from other countries and regions (Table 5). The influence of the fish fat content on PCB levels was significant in high trophic-level species of the Magdalena and Cauca River, in contrast to species from the Gulf of Urabá, where there was no association between fat content and pollutant presence. Similarly, the concentration of the congener hepta-chlorine (PCB 180) was higher in species with a high fat content because of its high lipophilicity and tendency to bioaccumulate across the food chain, while in species at the low trophic level the congeners with fewer chlorine substitutions (PCB 28/PCB 52) were detected, which could be related to the lower lipophilicity and greater solubility in water of those compounds. Furthermore, PCB levels in fish could be associated with reported data about the presence of Aroclor 1260. In addition, an association between fish aging and POPs levels was found. Finally, the variation in PCB was more influenced by the fish species due to their different habits, diet, trophic level and physiological characteristics, rather than the different levels of contamination at the source.

**Table 5:** Comparison of PCB levels in fish

Congeners	n	Range (ng$\cdot$g^−1^ fat)	Mean (ng$\cdot$g^−1^ fat)	Median (ng$\cdot$g^−1^ fat)	Reference
ΣPCB_6_	90	1.33 – 236	16.7	8.24	This study
Σdl-PCB		0.135—33.9	2.30	0.811	
ΣPCB_6_	173	–--	226 – 1090*	–-	(Szlinder-Richert et al., 2009)
Σdl-PCB		–-	51.3 – 247.3	–-	
ΣPCB_6_	187	4.5 – 711.6	121.3	71.5	(Tran-Lam et al., 2023)
Σdl-PCB			42.5	24.3	
ΣPCB_48_	12	–-	0.51 – 26.05*	–-	(Santos et al., 2020)
Total PCB	32	5.0 – 5600	–-	–-	(Cala & Södergren, 1999)
ΣPCB_7_	26	–-	24.3 – 106.4*	–-	(Manirakiza et al., 2002)

**ΣPCB**_**6**_**:** Indicator PCB, **Σdl-PCB:** dioxin-like-PCB, **ΣPCB48:** 48 congeners, ΣPCB_7_: Indicator PCB + PCB-118

^*^The mean depends on the fish species of the study

### PCDD/PCDF levels in fish

PCDD/PCDF were not detected in the fish samples. In all cases, the values were below the MQL. Consequently, no comparison was possible between fish species, sources, and patterns with respect to PCDD/PCDF levels. Results on PCDD/PCDF are presented in Table S5. The TEQ_PCDD/PCDF_ were calculated using the $\frac{1}{2}$MQL of each congener multiplied by the corresponding TEF value. Results were lower than the regulated value applied in Colombia. It is worth noting that these pollutants are among the most difficult POPs to analyze and detect because of their very low concentration in the environment and complex matrices. Although PCDD/PCDF were all below the MQLs of the analytical methodology, the results show the level at which these pollutants are not present in fish from the Antioquia region and give an overall perspective for future research regarding the presence of these pollutants in the country. The results are on similar lines to previous reports about POPs concentrations in other matrices, in which PCDD/PCDF were not detected or detected in very low concentrations (Aristizábal et al., 2011; Avila et al., 2022a, 2022b, 2022c, 2022d; Cortés et al., 2014, 2016; Pemberthy et al., 2016; Villa et al., 2016). In the 90 samples analyzed, the regulated Colombian limit and European limit was not exceeded using $\frac{1}{2}$MQL, confirming the low levels. Finally, the Environmental Quality Standards in biota (EQS_biota_) for sum of PCDD + PCDF + dl-PCB is 6.5 pg$\cdot$TEQ$\cdot$g^−1^ (EU, 2013). As was explained in before sections all results were below this threshold, therefore, the levels found do not pose an immediate risk to people who consume that fish.

## Conclusions

The levels of PCB, PCDD and PCDF in the fish samples were lower compared to those reported from other countries and regions, even in the tropics. The results suggest that the patterns and levels of POPs concentrations in fish in the region of Antioquia depend more on the characteristics of the fish, the level in the trophic food chain, diet, and habits than sample origin. In addition, the concentrations found were associated with background levels rather than hot spots or occasional contamination from any source.

PCB levels in fish from both rivers were strongly associated with the fat content, in contrast to marine fish species, because the marine food chain is much longer than for fish from terrestrial environment. Therefore, the biomagnification factor is higher in the sea (ocean) than on land (rivers and lakes). Furthermore, this study provided an overview of the current situation in the region and established a concentration baseline, which could support future research in the country and region.

Finally, POPs represent a challenge in developing countries and regions. An important barrier is due to the instrumentation and trained personnel that is needed to quantify these pollutants. The levels in the region of Antioquia were low, making them difficult to analyze. However, it is recommended to continue monitoring POPs to protect the health of the population and wildlife.

## Supplementary Information

Below is the link to the electronic supplementary material.

Supplementary file1 (DOCX 272 KB)

## Data Availability

No datasets were generated or analysed during the current study.
