# Supplementary material for: Occurrence, distribution, and levels of Polychlorinated Biphenyls (PCB), Polychlorinated Dibenzo-p–Dioxins (PCDD), and Polychlorinated Dibenzofurans (PCDF) in fish from the Antioquia Region, Colombia
Source: Environ Monit Assess. 2025 Apr 16;197(5):560. doi: 10.1007/s10661-025-13956-0 (PMC12003534; doi:10.1007/s10661-025-13956-0)
Supplement: Supplementary file 1 — Supplementary file1 (DOCX 272 KB) [file 10661_2025_13956_MOESM1_ESM.docx]

**SUPLEMENTARY MATERIAL**

**Occurrence, distribution, and levels of Polychlorinated Biphenyls (PCB), Polychlorinated Dibenzo-p–Dioxins (PCDD), and Polychlorinated Dibenzofurans (PCDF) in fish from the Department of Antioquia, Colombia**

Boris Santiago Avilaa∗, Diana Pemberthy Mendozaa, Henry Zúñiga-Beníteza,b, Gustavo A. Peñuelaa

aGrupo Diagnostico y Control de la Contaminación - GDCON, Facultad de Ingeniería, Sede de Investigación Universitaria (SIU), Universidad de Antioquia - UdeA, Calle 70 # 52 -21, Postal Code 050010, Medellín, Colombia.

bDepartamento de Ingeniería Química, Facultad de Ingeniería, Universidad de Antioquia - UdeA, Calle 70 # 52-21, Postal Code 050010, Medellín, Colombia.

^*^**Corresponding author**: Boris Santiago Ávila. Grupo GDCON, Facultad de Ingeniería, Sede de Investigación Universitaria (SIU), Universidad de Antioquia UdeA, Calle 70 # 52 -21, Postal Code 050010, Medellín, Colombia. E-mail: [boris.avila@udea.edu.co](mailto:boris.avila@udea.edu.co).

## **Standards**

Native PCB-mix 3 (PCB 28, PCB 52, PCB 101, PCB 118, PCB 138, PCB 153 and PCB 180) of 10 $\mu$g$\cdot$mL^-1^ in cyclohexane was purchased from Dr. Ehrenstorfer GmbH (Augsburg, Germany). The individual native standards of PCB 77, PCB 81, PCB 105, PCB 114, PCB 123, PCB 126, PCB 156, PCB 157, PCB 167, PCB 169, and PCB 189 were purchased from Dr. Ehrenstorfer GmbH (Augsburg, Germany) (purity $\geq$ 98%). Stock solutions of 500 $\mu$g$\cdot$mL^-1^ in hexane were prepared from the individual native standards, and a mixture of 10 $\mu$g$\cdot$mL^-1^ in hexane was made from the individual stock solutions. The labelled standard WP-LCS (^13^C_12_-PCB 77, ^13^C_12_-PCB 81, ^13^C_12_-PCB 105, ^13^C_12_-PCB 114, ^13^C_12_-PCB 118, ^13^C_12_-PCB 123, ^13^C_12_-PCB 126, ^13^C_12_-PCB 156, ^13^C_12_-PCB 157, ^13^C_12_-PCB 167, ^13^C_12_-PCB 169, and ^13^C_12_-PCB 189) was purchased from Wellington Laboratories (Ontario, Canada). A labelled PCB work solution of 50 pg$\cdot\mu$L^-1^ in isooctane was prepared from WP-LCS. Solutions of individual native standards of 2,3,7,8-TCDD to 10 $\mu$g$\cdot$mL^-1^ in toluene; 1,2,3,7,8-PeCDD, 1,2,3,4,7,8-HxCDD, 1,2,3,7,8,9-HxCDD, 1,2,3,6,7,8-HxCDD, 1,2,3,4,6,7,8-HpCDD, OCDD, 2,3,7,8-TCDF, 1,2,3,4,6,7,8-HpCDF, OCDF to 50 $\mu$g$\cdot$mL^-1^ in toluene; 2,3,4,7,8-PeCDF, 1,2,3,7,8-PeCDF, and 1,2,3,4,7,8-HxCDF to 5.0 $\mu$g$\cdot$mL^-1^ in toluene were purchased from Accustandard, Inc (New Haven, USA). Standard solutions of 1,2,3,6,7,8-HxCDF, 1,2,3,7,8,9-HxCDF, 2,3,4,6,7,8-HxCDF, and 1,2,3,4,7,8,9-HpCDF to 50 $\mu$g$\cdot$mL^-1^ in nonane were purchased from Cambridge Isotope Laboratories, inc (Tewksbury, USA). A work mixture with the 17 PCDD/PCDF congeners was prepared at 0.50 $\mu$g$\cdot$mL^-1^ in isooctane. The labelled standard EPA-1613LCS (^13^C_12_-2,3,7,8-TCDD, ^13^C_12_-1,2,3,7,8-PeCDD, ^13^C_12_-1,2,3,4,7,8-HxCDD, ^13^C_12_-1,2,3, 6,7,8-HxCDD, ^13^C_12_-1,2,3,4,6,7,8-HpCDD, ^13^C_12_-OCDD, ^13^C_12_-2,3,7,8-TCDF, ^13^C_12_-1,2, 3,7,8-PeCDF, ^13^C_12_-2,3,4,7,8-PeCDF, ^13^C_12_-1,2,3,4,7,8-HxCDF, ^13^C_12_-1,2,3,7,8,9-HxCDF, ^13^C_12_-1,2,3,6,7,8-HxCDF, ^13^C_12_-2,3,4,6,7,8-HxCDF, ^13^C_12_-1,2,3,4, 6,7,8-HpCDF, and ^13^C_12_-1,2,3,4,7,8,9-HpCDF) was purchased from Wellington Laboratories (Ontario, Canada). A labelled PCDD/PCDF work solution of 5.0 pg$\cdot\mu$L^-1^ in isooctane was prepared from EPA-1613LCS. All solutions were stored at -20 ${}^{\circ}$C $\pm$ 3 ${}^{\circ}$C.

**Clean-up columns**

The acid/basic column consisted of bottom to top: glass wool, 20.0 g of basic silica gel (33.0% NaOH, w/w)*,* 5.0 g of neutral silica gel, 40.0 g of acid silica gel (40.0% H_2_SO_4_, w/w), and 5.0 g of anhydrous sodium sulfate. The column was pre-conditioned with 100.0 mL of hexane prior to use. Column dimensions were 30 cm x 5.0 cm i.d (L x W). The nitrate column consisted of bottom to top: glass wool, 3.0 g of 10.0% AgNO_3_ (0.33 g AgNO_3_ in 1.0 mL of water by 3.0 g of neutral silica gel) activated at 180.0°C for 1.0 h, and 1.0 g of sodium sulfate anhydrous. The column was pre-conditioned with 25.0 mL of hexane. The dimensions of the column were 10.5 cm x 1.5 cm i.d (L x W). The florisil column consisted of 6.0 g of florisil. The column was pre-conditioned with 25.0 mL of hexane prior to use. The dimensions of the column were 25.0 cm x 1.5 cm i.d (L x W). The carbon column consisted of a SPE Supelclean ENVI-Carb cartridge of 250 mg purchased from Merck (Darmstadt, Germany). The cartridge was pre–conditioned with 5.0 mL of hexane.

## **Validation results and uncertainty.**

The validation of the method was performed to evaluate the performance. The parameters assessed were selectivity, linearity, method quantification limit (MQL), precision, and trueness. Furthermore, the uncertainty for each congener was calculated and the TEQ combined uncertainty for dl-PCB and PCDD/PCDF.

For PCB, MQLs were between 10.0 pg·g sample^-1^ and 25.5 pg·g sample^-1^, RSDs were between 2.50% and 14.0% and the percentage of recoveries were between 71.2% and 98.3%. For PCDD/F, MQLs were between 6.70 pg·g sample^-1^ and 87.3 pg·g sample^-1^, RSDs were between 4.77% and 13.9% and the percentage of recoveries were between 73.1% and 82.8%. In all cases the determinant coefficient was higher than 0.994. Furthermore, regression and lack of fit hypothesis were fulfilled. The results indicated that the performance of the method was adequate for the analysis of PCB and PCDD/F.

**TABLE S1. Summary of validation results.**

| **Compound** | **R^2^** | **MQL pg/g sample** | **RSD(%)** | **%REC** | **U_exp_(%)** |
| --- | --- | --- | --- | --- | --- |
| PCB-28 | 0.994 | 23.9 | 10.30 | 74.2 | 42.6 |
| PCB-52 | 0.995 | 16.9 | 10.80 | 81.0 | 43.5 |
| PCB-101 | 0.995 | 10.0 | 4.77 | 74.1 | 21.4 |
| PCB-138 | 0.997 | 12.5 | 2.50 | 94.4 | 17.1 |
| PCB-153 | 0.998 | 13.1 | 5.60 | 82.4 | 25.8 |
| PCB-180 | 0.995 | 10.7 | 11.40 | 94.5 | 34.7 |
| PCB-77 | 0.995 | 15.9 | 12.40 | 77.1 | 36.6 |
| PCB-81 | 0.997 | 25.5 | 6.22 | 78.8 | 29.7 |
| PCB-105 | 0.997 | 11.3 | 11.70 | 80.3 | 36.8 |
| PCB-114 | 0.997 | 14.6 | 8.57 | 91.3 | 29.1 |
| PCB-118 | 0.997 | 10.3 | 8.33 | 92.6 | 29.1 |
| PCB-123 | 0.997 | 14.9 | 14.00 | 77.3 | 38.2 |
| PCB-126 | 0.997 | 13.4 | 9.06 | 75.6 | 35.7 |
| PCB-156 | 0.997 | 10.4 | 11.20 | 85.6 | 37.6 |
| PCB-157 | 0.997 | 11.7 | 10.20 | 90.3 | 37.6 |
| PCB-167 | 0.997 | 11.4 | 6.86 | 95.5 | 27.0 |
| PCB-169 | 0.997 | 10.6 | 13.40 | 71.2 | 37.9 |
| PCB-189 | 0.997 | 11.7 | 7.20 | 98.3 | 31.4 |
| 2,3,7,8-TCDF | 0.999 | 8.53 | 4.77 | 75.6 | 23.8 |
| 2,3,7,8-TCDD | 0.998 | 9.99 | 9.46 | 76.8 | 37.8 |
| 1,2,3,7,8-PeCDF | 0.998 | 12.40 | 12.50 | 78.1 | 43.3 |
| 2,3,4,7,8-PeCDF | 0.999 | 8.33 | 12.00 | 82.8 | 41.5 |
| 1,2,3,7,8-PeCDD | 0.996 | 38.90 | 7.66 | 75.0 | 62.4 |
| 1,2,3,4,7,8-HxCDF | 0.998 | 7.70 | 10.70 | 82.8 | 36.6 |
| 1,2,3,6,7,8-HxCDF | 0.997 | 10.50 | 12.30 | 79.3 | 44.5 |
| 1,2,3,4,7,8-HxCDD | 0.999 | 38.90 | 10.30 | 82.3 | 63.3 |
| 2,3,4,6,7,8-HxCDF | 0.997 | 8.37 | 11.50 | 76.9 | 37.4 |
| 1,2,3,6,7,8-HxCDD | 0.998 | 38.90 | 8.94 | 76.3 | 64.7 |
| 1,2,3,7,8,9-HxCDD | 0.996 | 38.90 | 6.77 | 79.1 | 55.9 |
| 1,2,3,7,8,9-HxCDF | 0.995 | 6.70 | 13.10 | 75.1 | 44.8 |
| 1,2,3,4,6,7,8-HpCDF | 0.996 | 30.50 | 12.60 | 77.6 | 60.2 |
| 1,2,3,4,6,7,8-HpCDD | 0.998 | 8.06 | 13.90 | 73.1 | 42.9 |
| 1,2,3,4,7,8,9-HpCDF | 0.997 | 7.65 | 9.92 | 77.6 | 34.0 |
| OCDD | 0.995 | 87.30 | 9.92 | 76.8 | 99.3 |
| OCDF | 0.996 | 13.70 | 7.92 | 74.9 | 38.4 |

R^2^ = Determinant coefficient, MQL = Method Quantification Limit, RSD = Relative Standard Deviation, REC = Percentage of recovery, Uexp = Expanded Uncertainty (k=2).

**TABLE S1. Ions for PCB quantification (SIM).**

| **Compound** | **RT (min)** | **Target** | **Q1** | **Q2** |
| --- | --- | --- | --- | --- |
| PCB 28 | 12.0 | 256 | 258 | 260 |
| PCB 52 | 13.2 | 292 | 290 | 294 |
| PCB 101 | 16.7 | 326 | 328 | 324 |
| PCB 81 | 18.1 | 292 | 290 | 294 |
| PCB 77 | 18.6 | 292 | 290 | 294 |
| PCB 123 | 19.5 | 326 | 328 | 324 |
| PCB 118 | 19.7 | 326 | 328 | 324 |
| PCB 114 | 20.2 | 326 | 328 | 324 |
| PCB 153 | 20.8 | 360 | 362 | 358 |
| PCB 105 | 21.0 | 326 | 328 | 324 |
| PCB 138 | 22.1 | 360 | 362 | 358 |
| PCB 126 | 22.7 | 326 | 328 | 324 |
| PCB 167 | 23.6 | 360 | 362 | 358 |
| PCB 156 | 24.8 | 360 | 362 | 358 |
| PCB 157 | 25.0 | 360 | 362 | 358 |
| PCB 180 | 25.6 | 394 | 396 | 398 |
| PCB 169 | 26.8 | 360 | 362 | 358 |
| PCB 189 | 28.7 | 394 | 396 | 398 |
| 13C PCB 81 | 18.1 | 304 | 302 | 306 |
| 13C PCB 77 | 18.6 | 304 | 302 | 306 |
| 13C PCB 123 | 19.5 | 338 | 340 | 336 |
| 13C PCB 118 | 19.7 | 338 | 340 | 336 |
| 13C PCB 114 | 20.2 | 338 | 340 | 336 |
| 13C PCB 105 | 21.0 | 338 | 340 | 336 |
| 13C PCB 126 | 22.7 | 338 | 340 | 336 |
| 13C PCB 167 | 23.6 | 372 | 374 | 370 |
| 13C PCB 156 | 24.8 | 372 | 374 | 370 |
| 13C PCB 157 | 25.0 | 372 | 374 | 370 |
| 13C PCB 169 | 26.8 | 372 | 374 | 370 |
| 13C PCB 189 | 28.7 | 406 | 408 | 410 |

RT = retention time, Target = quantification ion, Q1 = qualification ion, Q2 = qualification ion.

**TABLE S2: Ions for PCDD/PCDF quantification (SIM).**

| **Compound** | **RT (min)** | **Target** | **Q1** | **Q2** |
| --- | --- | --- | --- | --- |
| 2,3,7,8-TCDF | 25.1 | 306 | 304 | 308 |
| 2,3,7,8-TCDD | 26.6 | 322 | 320 | 324 |
| 1,2,3,7,8-PeCDF | 30.9 | 340 | 342 | 338 |
| 2,3,4,7,8-PeCDF | 32.5 | 340 | 342 | 338 |
| 1,2,3,7,8-PeCDD | 32.8 | 354 | 358 | 356 |
| 1,2,3,4,7,8-HxCDF | 37.2 | 374 | 376 | 372 |
| 1,2,3,6,7,8-HxCDF | 37.4 | 374 | 376 | 372 |
| 2,3,4,6,7,8-HxCDF | 38.6 | 374 | 376 | 372 |
| 1,2,3,4,7,8-HxCDD | 38.9 | 390 | 392 | 388 |
| 1,2,3,6,7,8-HxCDD | 38.9 | 390 | 392 | 388 |
| 1,2,3,7,8,9-HxCDD | 39.5 | 390 | 392 | 388 |
| 1,2,3,7,8,9-HxCDF | 40.2 | 374 | 376 | 372 |
| 1,2,3,4,6,7,8-HpCDF | 43.0 | 408 | 410 | 412 |
| 1,2,3,4,6,7,8-HpCDD | 45.2 | 424 | 426 | 428 |
| 1,2,3,4,7,8,9-HpCDF | 46.2 | 408 | 410 | 412 |
| OCDD | 51.0 | 460 | 458 | 462 |
| OCDF | 51.5 | 442 | 444 | 446 |
| 13C-2,3,7,8-TCDF | 25.1 | 318 | 316 | 320 |
| 13C-2,3,7,8-TCDD | 26.6 | 334 | 332 | 336 |
| 13C-1,2,3,7,8-PeCDF | 30.9 | 352 | 354 | 350 |
| 13C-2,3,4,7,8-PeCDF | 32.5 | 352 | 354 | 350 |
| 13C-1,2,3,7,8-PeCDD | 32.8 | 366 | 370 | 368 |
| 13C-1,2,3,4,7,8-HxCDF | 37.2 | 386 | 388 | 384 |
| 13C-1,2,3,6,7,8-HxCDF | 37.4 | 386 | 388 | 384 |
| 13C-2,3,4,6,7,8-HxCDF | 38.6 | 386 | 388 | 384 |
| 13C-1,2,3,4,7,8-HxCDD | 38.9 | 402 | 404 | 400 |
| 13C-1,2,3,6,7,8-HxCDD | 38.9 | 402 | 404 | 400 |
| 13C-1,2,3,7,8,9-HxCDF | 40.2 | 386 | 388 | 384 |
| 13C-1,2,3,4,6,7,8-HpCDF | 43.0 | 420 | 422 | 424 |
| 13C-1,2,3,4,6,7,8-HpCDD | 45.2 | 436 | 438 | 440 |
| 13C-1,2,3,4,7,8,9-HpCDF | 46.2 | 420 | 422 | 424 |
| 13C-OCDD | 51.0 | 472 | 470 | 474 |

RT = retention time, Target = quantification ion, Q1 = qualification ion, Q2 = qualification ion.

## **Results.**

**TABLE S3 (a): Individual results for PCB in fish samples (n=90, PCB, pg/g w.w).**

| **ID** | **PCB 28** | **PCB 52** | **PCB 101** | **PCB 153** | **PCB 138** | **PCB 180** |
| --- | --- | --- | --- | --- | --- | --- |
| M1-1 | 5.05 | 53.90 | 44.00 | 86.00 | 51.80 | 48.60 |
| M1-2 | 11.7 | 29.4 | 47.5 | 136.0 | 77.1 | 128.0 |
| M1-3 | 6.57 | 8.64 | 8.91 | 16.70 | 7.36 | 34.30 |
| M1-4 | 15.8 | 12.8 | 12.4 | 12.9 | 6.5 | 127.0 |
| M1-5 | 1.84 | 5.58 | 6.07 | 28.50 | 21.10 | 42.30 |
| M1-6 | 14.7 | 34.1 | 36.2 | 62.6 | 38.7 | 98.5 |
| M1-7 | 10.5 | 20.3 | 22.6 | 43.1 | 20.9 | 71.8 |
| M1-8 | 400.0 | 772.0 | 558.0 | 975.0 | 704.0 | 1140.0 |
| M1-9 | 25.6 | 71.9 | 32.0 | 99.0 | 58.1 | 57.9 |
| M1-10 | 1.30 | 164.00 | 46.50 | 152.00 | 90.60 | 133.00 |
| M1-11 | 6.70 | 8.35 | 5.70 | 11.50 | 7.89 | 5.66 |
| M1-12 | 56.2 | 45.2 | 8.0 | 6.3 | <0.63 | 16.4 |
| M1-13 | 4.83 | 8.01 | 2.14 | 4.17 | 3.69 | 3.74 |
| M1-14 | 9.45 | 13.90 | 3.78 | 8.60 | 8.82 | 10.40 |
| M1-15 | 12.5 | 17.5 | 6.8 | 8.8 | 5.9 | 12.3 |
| C1-1 | 26.9 | 113.0 | 122.0 | 375.0 | 234.0 | 435.0 |
| C1-2 | 17.6 | 91.1 | 83.9 | 183.0 | 126.0 | 108.0 |
| C1-3 | 16.7 | 54.7 | 100.0 | 328.0 | 198.0 | 405.0 |
| C1-4 | <0.300 | <0.211 | 30.1 | 23.7 | 15.8 | 83.2 |
| C1-5 | 6.04 | 24.10 | 26.80 | 73.60 | 48.70 | 67.20 |
| C1-6 | 8.36 | 42.20 | 38.00 | 126.00 | 82.70 | 110.00 |
| C1-7 | <0.205 | 43.0 | 52.7 | 126.0 | 89.4 | 53.70 |
| C1-8 | 10.0 | 40.6 | 75.0 | 159.0 | 87.9 | 287.00 |
| C1-9 | 19.4 | 79.4 | 59.6 | 102.0 | 67.2 | 94.90 |
| C1-10 | 20.5 | 56.2 | 52.0 | 86.2 | 55.6 | 119.00 |
| C1-11 | 3.66 | 24.70 | 4.52 | 4.46 | 2.81 | 4.34 |
| C1-12 | 30.3 | 62.2 | 20.6 | 20.4 | 13.3 | 48.3 |
| C1-13 | 22.4 | 38.4 | 8.8 | 11.1 | 8.4 | <0.151 |
| C1-14 | 34.3 | 59.2 | 24.5 | 23.0 | 18.4 | 37.1 |
| C1-15 | 9.81 | 27.20 | 12.10 | 11.20 | 7.97 | 16.80 |
| U1-1 | 6.18 | 5.19 | 4.85 | 18.60 | 11.00 | 21.20 |
| U1-2 | 4.37 | 4.32 | 2.80 | 9.29 | 4.98 | 32.40 |
| U1-3 | 4.46 | 4.48 | 6.79 | 27.90 | 15.40 | 65.40 |
| U1-4 | 1.87 | 2.78 | 3.21 | 11.50 | 6.89 | 17.60 |
| U1-5 | 6.10 | 6.21 | 8.62 | 29.30 | 14.40 | 56.30 |
| U1-6 | 17.3 | 86.1 | 153.0 | 216.0 | 114.0 | 620.0 |
| U1-7 | 18.3 | 50.0 | 49.3 | 56.4 | 44.5 | 61.0 |
| U1-8 | 14.1 | 107.0 | 141.0 | 234.0 | 165.0 | 200.0 |
| U1-9 | 33.1 | 228.0 | 183.0 | 187.0 | 168.0 | 165.0 |
| U1-10 | 8.88 | 30.40 | 32.60 | 86.40 | 53.40 | 135.00 |
| U1-11 | 22.1 | 36.3 | 12.1 | 15.2 | 9.0 | 15.3 |
| U1-12 | 31.3 | 65.6 | 12.5 | 25.5 | 6.6 | 21.9 |
| U1-13 | 9.83 | 10.70 | 8.71 | 17.30 | 8.78 | 21.40 |
| U1-14 | 12.1 | 11.6 | 5.7 | 9.7 | 7.0 | 14.9 |
| U1-15 | 10.0 | 26.1 | 55.0 | <0.637 | <0.605 | 35.1 |
| M2-1 | 6.46 | 14.40 | 23.40 | 50.50 | 32.10 | 83.80 |
| M2-2 | 3.64 | 9.44 | 19.00 | 43.70 | 26.50 | 61.20 |
| M2-3 | 6.73 | 26.10 | 44.50 | 98.30 | 59.10 | 149.00 |
| M2-4 | 29.5 | 97.0 | 173.0 | 678.0 | 396.0 | 670.0 |
| M2-5 | 10.4 | 23.2 | 35.0 | 77.1 | 46.0 | 104.0 |
| M2-6 | <0.243 | 70.8 | 274.0 | 397.0 | 231.0 | 422.0 |
| M2-7 | 60.5 | 151.0 | 218.0 | 706.0 | 387.0 | 770.0 |
| M2-8 | 82.2 | 107.0 | 72.1 | 287.0 | 184.0 | 78.8 |
| M2-9 | <0.278 | 84.5 | 177.0 | 1330.0 | 803.0 | 452.0 |
| M2-10 | 8.82 | 45.50 | 70.10 | 223.00 | 131.00 | 287.00 |
| M2-11 | 18.9 | 27.6 | 6.0 | 8.8 | 9.2 | <0.539 |
| M2-12 | 6.68 | 9.77 | 4.38 | 5.99 | 1.89 | 8.48 |
| M2-13 | 27.4 | 40.4 | 18.8 | 42.7 | 31.1 | 48.2 |
| M2-14 | 18.9 | 25.2 | 27.8 | 47.0 | 31.3 | 87.3 |
| M2-15 | 28.5 | 45.2 | 20.4 | 38.6 | 24.8 | 56.7 |
| C2-1 | 33.3 | 87.7 | 41.0 | 63.7 | 40.2 | 51.4 |
| C2-2 | 5.16 | 14.40 | 8.98 | 17.40 | 11.00 | 18.70 |
| C2-3 | 10.3 | 26.0 | 25.7 | 53.4 | 31.6 | 76.0 |
| C2-4 | 15.5 | 27.4 | 16.2 | 48.1 | 27.0 | 58.0 |
| C2-5 | 7.79 | 14.70 | 9.23 | 9.90 | 7.10 | 12.60 |
| C2-6 | 24.5 | 39.3 | 27.0 | 46.3 | 28.7 | 146.0 |
| C2-7 | 8.57 | 11.10 | 11.30 | 34.20 | 22.30 | 16.10 |
| C2-8 | <0.255 | 62.9 | 46.3 | 102.0 | 65.8 | 131.0 |
| C2-9 | 34.2 | 36.6 | 98.0 | 336.0 | 172.0 | 31.6 |
| C2-10 | <0.245 | 71.2 | 59.7 | 96.8 | 67.0 | 63.6 |
| C2-11 | 37.7 | 74.2 | 7.3 | 6.3 | 4.9 | 16.3 |
| C2-12 | 10.0 | 10.7 | 7.5 | 10.5 | 4.4 | 12.1 |
| C2-13 | 16.1 | 65.2 | 12.2 | 11.1 | 8.5 | 7.9 |
| C2-14 | 1.62 | 10.00 | 5.06 | 8.11 | 5.29 | 10.70 |
| C2-15 | 4.91 | 19.00 | 8.94 | 12.10 | 8.49 | 9.32 |
| U2-1 | 4.83 | 2.32 | 7.92 | 26.30 | 13.80 | 53.20 |
| U2-2 | 35.1 | 17.7 | 7.7 | 11.6 | 5.0 | 47.4 |
| U2-3 | 6.31 | 7.41 | 13.60 | 94.50 | 56.00 | 104.00 |
| U2-4 | 1.45 | 1.94 | 3.13 | 30.20 | 15.40 | 47.40 |
| U2-5 | 8.65 | 17.50 | 32.00 | 71.00 | 60.20 | 340.00 |
| U2-6 | 4.44 | 8.07 | 12.20 | 21.50 | 13.00 | 61.20 |
| U2-7 | 8.03 | 8.73 | 14.50 | 62.70 | 40.40 | 49.20 |
| U2-8 | 18.8 | 13.3 | 10.4 | 33.4 | 20.5 | 42.4 |
| U2-9 | 8.01 | 8.99 | 7.75 | 23.10 | 8.84 | 28.10 |
| U2-10 | 4.21 | 7.71 | 8.20 | 11.50 | 7.06 | 19.40 |
| U2-11 | 8.16 | 14.20 | 6.22 | 8.46 | 3.55 | 11.90 |
| U2-12 | 35.9 | 38.2 | 14.1 | 21.6 | 12.0 | 36.5 |
| U2-13 | 11.4 | 16.0 | 9.0 | 8.6 | 4.6 | <0.291 |
| U2-14 | 10.4 | 25.9 | 4.3 | <0.251 | <0.238 | 4.8 |
| U2-15 | 14.3 | 33.9 | 11.7 | 18.9 | 14.8 | 30.0 |

**TABLE S3 (b): Individual results of PCB in the fish samples (n=90, PCB, ng/g fat).**

| **ID** | **PCB 28** | **PCB 52** | **PCB 101** | **PCB 153** | **PCB 138** | **PCB 180** |
| --- | --- | --- | --- | --- | --- | --- |
| M1-1 | 0.101 | 1.080 | 0.878 | 1.720 | 1.030 | 0.971 |
| M1-2 | 0.288 | 0.721 | 1.160 | 3.330 | 1.890 | 3.140 |
| M1-3 | 0.593 | 0.780 | 0.804 | 1.500 | 0.665 | 3.100 |
| M1-4 | 3.020 | 2.440 | 2.360 | 2.470 | 1.240 | 24.300 |
| M1-5 | 0.023 | 0.070 | 0.077 | 0.359 | 0.266 | 0.533 |
| M1-6 | 0.844 | 1.950 | 2.070 | 3.590 | 2.220 | 5.640 |
| M1-7 | 1.530 | 2.980 | 3.320 | 6.330 | 3.070 | 10.500 |
| M1-8 | 12.800 | 24.800 | 17.900 | 31.300 | 22.600 | 36.500 |
| M1-9 | 0.296 | 0.831 | 0.370 | 1.140 | 0.671 | 0.669 |
| M1-10 | 0.007 | 0.884 | 0.251 | 0.820 | 0.489 | 0.716 |
| M1-11 | 2.890 | 3.600 | 2.460 | 4.970 | 3.400 | 2.440 |
| M1-12 | 5.300 | 4.260 | 0.756 | 0.598 | <0.0594 | 1.550 |
| M1-13 | 0.307 | 0.509 | 0.136 | 0.265 | 0.234 | 0.238 |
| M1-14 | 0.333 | 0.488 | 0.133 | 0.303 | 0.311 | 0.368 |
| M1-15 | 4.140 | 5.780 | 2.240 | 2.920 | 1.950 | 4.080 |
| C1-1 | 0.572 | 2.410 | 2.600 | 7.960 | 4.970 | 9.230 |
| C1-2 | 0.198 | 1.020 | 0.943 | 2.060 | 1.420 | 1.210 |
| C1-3 | 0.414 | 1.360 | 2.490 | 8.140 | 4.920 | 10.100 |
| C1-4 | <0.00728 | <0.00513 | 0.731 | 0.575 | 0.384 | 2.020 |
| C1-5 | 0.223 | 0.892 | 0.991 | 2.720 | 1.800 | 2.490 |
| C1-6 | 0.154 | 0.778 | 0.700 | 2.330 | 1.520 | 2.030 |
| C1-7 | <0.0033 | 0.695 | 0.850 | 2.030 | 1.440 | 0.866 |
| C1-8 | 0.280 | 1.140 | 2.110 | 4.470 | 2.470 | 8.070 |
| C1-9 | 0.455 | 1.870 | 1.400 | 2.410 | 1.580 | 2.230 |
| C1-10 | 0.718 | 1.960 | 1.820 | 3.020 | 1.940 | 4.170 |
| C1-11 | 0.133 | 0.898 | 0.164 | 0.162 | 0.102 | 0.158 |
| C1-12 | 0.412 | 0.847 | 0.281 | 0.278 | 0.181 | 0.658 |
| C1-13 | 0.465 | 0.795 | 0.182 | 0.230 | 0.175 | <0.00313 |
| C1-14 | 0.533 | 0.921 | 0.381 | 0.357 | 0.286 | 0.577 |
| C1-15 | 0.171 | 0.476 | 0.212 | 0.195 | 0.139 | 0.293 |
| U1-1 | 0.821 | 0.691 | 0.645 | 2.480 | 1.470 | 2.820 |
| U1-2 | 0.596 | 0.589 | 0.382 | 1.270 | 0.679 | 4.420 |
| U1-3 | 0.491 | 0.494 | 0.748 | 3.070 | 1.690 | 7.200 |
| U1-4 | 0.082 | 0.122 | 0.141 | 0.505 | 0.303 | 0.772 |
| U1-5 | 0.749 | 0.763 | 1.060 | 3.600 | 1.770 | 6.920 |
| U1-6 | 3.380 | 16.900 | 29.900 | 42.400 | 22.300 | 121.000 |
| U1-7 | 5.790 | 15.800 | 15.600 | 17.800 | 14.100 | 19.300 |
| U1-8 | 1.450 | 11.100 | 14.600 | 24.100 | 17.000 | 20.600 |
| U1-9 | 1.470 | 10.100 | 8.120 | 8.300 | 7.480 | 7.310 |
| U1-10 | 0.526 | 1.800 | 1.930 | 5.120 | 3.170 | 8.030 |
| U1-11 | 1.280 | 2.110 | 0.701 | 0.883 | 0.524 | 0.891 |
| U1-12 | 1.170 | 2.450 | 0.469 | 0.953 | 0.245 | 0.820 |
| U1-13 | 0.939 | 1.030 | 0.833 | 1.650 | 0.838 | 2.040 |
| U1-14 | 1.360 | 1.310 | 0.640 | 1.090 | 0.788 | 1.680 |
| U1-15 | 0.689 | 1.790 | 3.780 | <0.0438 | <0.0416 | 2.410 |
| M2-1 | 0.245 | 0.548 | 0.887 | 1.920 | 1.220 | 3.180 |
| M2-2 | 0.138 | 0.357 | 0.720 | 1.650 | 1.000 | 2.310 |
| M2-3 | 0.205 | 0.792 | 1.350 | 2.990 | 1.800 | 4.540 |
| M2-4 | 0.438 | 1.440 | 2.570 | 10.100 | 5.890 | 9.960 |
| M2-5 | 0.499 | 1.110 | 1.680 | 3.700 | 2.200 | 4.990 |
| M2-6 | <0.00224 | 0.652 | 2.520 | 3.660 | 2.130 | 3.890 |
| M2-7 | 0.598 | 1.490 | 2.160 | 6.980 | 3.820 | 7.610 |
| M2-8 | 1.360 | 1.770 | 1.190 | 4.740 | 3.040 | 1.300 |
| M2-9 | <0.00235 | 0.714 | 1.500 | 11.300 | 6.790 | 3.820 |
| M2-10 | 0.158 | 0.817 | 1.260 | 3.990 | 2.360 | 5.150 |
| M2-11 | 1.290 | 1.880 | 0.412 | 0.601 | 0.628 | <0.0368 |
| M2-12 | 0.732 | 1.070 | 0.480 | 0.656 | 0.208 | 0.929 |
| M2-13 | 1.080 | 1.600 | 0.744 | 1.690 | 1.230 | 1.900 |
| M2-14 | 0.407 | 0.543 | 0.600 | 1.010 | 0.675 | 1.880 |
| M2-15 | 1.360 | 2.150 | 0.975 | 1.840 | 1.180 | 2.700 |
| C2-1 | 0.977 | 2.580 | 1.200 | 1.870 | 1.180 | 1.510 |
| C2-2 | 0.213 | 0.594 | 0.371 | 0.719 | 0.455 | 0.774 |
| C2-3 | 0.779 | 1.960 | 1.940 | 4.040 | 2.390 | 5.740 |
| C2-4 | 2.380 | 4.210 | 2.490 | 7.400 | 4.150 | 8.930 |
| C2-5 | 0.337 | 0.634 | 0.399 | 0.428 | 0.307 | 0.544 |
| C2-6 | 0.222 | 0.357 | 0.246 | 0.421 | 0.261 | 1.330 |
| C2-7 | 0.229 | 0.297 | 0.300 | 0.912 | 0.593 | 0.428 |
| C2-8 | <0.00936 | 2.310 | 1.700 | 3.750 | 2.410 | 4.820 |
| C2-9 | 0.381 | 0.407 | 1.090 | 3.730 | 1.920 | 0.351 |
| C2-10 | <0.00308 | 0.895 | 0.751 | 1.220 | 0.843 | 0.800 |
| C2-11 | 1.310 | 2.580 | 0.253 | 0.219 | 0.171 | 0.568 |
| C2-12 | 0.448 | 0.476 | 0.336 | 0.468 | 0.196 | 0.540 |
| C2-13 | 0.522 | 2.110 | 0.397 | 0.359 | 0.276 | 0.256 |
| C2-14 | 0.067 | 0.410 | 0.207 | 0.332 | 0.217 | 0.439 |
| C2-15 | 0.234 | 0.904 | 0.426 | 0.574 | 0.404 | 0.443 |
| U2-1 | 0.696 | 0.335 | 1.140 | 3.800 | 1.990 | 7.670 |
| U2-2 | 7.790 | 3.940 | 1.720 | 2.580 | 1.100 | 10.500 |
| U2-3 | 0.466 | 0.547 | 1.000 | 6.980 | 4.140 | 7.710 |
| U2-4 | 0.369 | 0.496 | 0.799 | 7.720 | 3.930 | 12.100 |
| U2-5 | 0.349 | 0.707 | 1.290 | 2.860 | 2.430 | 13.700 |
| U2-6 | 0.171 | 0.311 | 0.468 | 0.826 | 0.501 | 2.350 |
| U2-7 | 0.360 | 0.392 | 0.653 | 2.820 | 1.810 | 2.210 |
| U2-8 | 0.607 | 0.430 | 0.335 | 1.080 | 0.662 | 1.370 |
| U2-9 | 0.530 | 0.595 | 0.514 | 1.530 | 0.585 | 1.860 |
| U2-10 | 0.246 | 0.450 | 0.479 | 0.672 | 0.412 | 1.130 |
| U2-11 | 1.070 | 1.870 | 0.818 | 1.110 | 0.467 | 1.570 |
| U2-12 | 1.980 | 2.110 | 0.776 | 1.190 | 0.661 | 2.020 |
| U2-13 | 2.790 | 3.920 | 2.200 | 2.100 | 1.130 | <0.0715 |
| U2-14 | 4.520 | 11.300 | 1.850 | <0.109 | <0.104 | 2.110 |
| U2-15 | 0.761 | 1.810 | 0.623 | 1.010 | 0.791 | 1.600 |

**TABLE S3 (c): Individual results of dl-PCB in the fish samples (n=90, dl-PCB, pg/g w.w).**

| **ID** | **PCB 81** | **PCB 77** | **PCB 123** | **PCB 118** | **PCB 114** | **PCB 105** | **PCB 126** | **PCB 167** | **PCB 156** | **PCB 157** | **PCB 169** | **PCB 189** |
| --- | --- | --- | --- | --- | --- | --- | --- | --- | --- | --- | --- | --- |
| M1-1 | <0.245 | <0.153 | <0.143 | 23.90 | <0.0876 | <0.108 | <0.128 | <0.11 | 5.76 | <0.112 | <0.102 | <0.113 |
| M1-2 | <0.288 | <0.18 | <0.168 | 25.4 | <0.103 | 3.6 | <0.151 | <0.129 | <0.118 | <0.132 | <0.12 | <0.132 |
| M1-3 | <0.332 | <0.208 | <0.194 | 3.13 | <0.119 | <0.147 | <0.174 | <0.149 | <0.136 | <0.153 | <0.138 | <0.153 |
| M1-4 | <0.297 | <0.185 | <0.173 | 4.8 | <0.106 | <0.131 | <0.155 | <0.133 | <0.122 | <0.136 | <0.123 | <0.136 |
| M1-5 | <0.293 | <0.183 | <0.171 | 7.87 | <0.105 | <0.13 | <0.154 | <0.131 | 2.09 | <0.135 | <0.122 | <0.135 |
| M1-6 | <0.335 | <0.209 | <0.196 | 10.4 | <0.12 | 4.0 | <0.175 | <0.15 | <0.137 | <0.154 | <0.139 | <0.154 |
| M1-7 | <0.322 | <0.201 | <0.188 | 10.0 | <0.115 | 3.2 | <0.169 | <0.144 | <0.132 | <0.148 | <0.134 | <0.148 |
| M1-8 | <0.285 | 16.8 | <0.166 | 290.0 | 22.5 | 185.0 | 4.2 | 21.8 | 10.9 | 7.6 | <0.118 | 7.8 |
| M1-9 | <0.27 | <0.169 | <0.158 | 137.0 | <0.0966 | 56.1 | <0.142 | <0.121 | <0.111 | <0.124 | <0.112 | <0.124 |
| M1-10 | <0.309 | <0.193 | <0.18 | 19.00 | <0.11 | 4.00 | 2.95 | 1.42 | 7.50 | <0.142 | <0.128 | <0.142 |
| M1-11 | <0.311 | <0.194 | <0.182 | 3.80 | <0.111 | <0.138 | 0.87 | <0.139 | <0.128 | <0.143 | <0.129 | <0.143 |
| M1-12 | <1.29 | <0.804 | <0.753 | <0.521 | <0.46 | <0.57 | <0.674 | <0.577 | <0.528 | <0.591 | <0.535 | <0.592 |
| M1-13 | <1 | <0.627 | <0.587 | 5.73 | <0.359 | <0.444 | <0.526 | <0.45 | <0.411 | <0.461 | <0.417 | <0.461 |
| M1-14 | <1.45 | <0.905 | <0.847 | 7.70 | <0.518 | <0.641 | <0.759 | <0.649 | 1.77 | <0.665 | <0.602 | <0.666 |
| M1-15 | <0.659 | <0.411 | <0.385 | 2.1 | <0.235 | <0.291 | <0.345 | <0.295 | <0.27 | <0.302 | <0.274 | <0.303 |
| C1-1 | <0.238 | 1.7 | <0.139 | 70.2 | <0.0851 | 22.0 | <0.125 | 9.8 | 6.6 | 3.4 | <0.0991 | <0.11 |
| C1-2 | <0.287 | <0.179 | <0.167 | 53.2 | <0.102 | 22.9 | <0.15 | <0.128 | 9.3 | <0.131 | <0.119 | <0.132 |
| C1-3 | <0.287 | <0.179 | 17.9 | 85.5 | <0.102 | 22.4 | <0.15 | 6.2 | 6.1 | 3.9 | <0.119 | 4.1 |
| C1-4 | <0.319 | <0.199 | <0.186 | 10.6 | <0.114 | <0.141 | <0.167 | <0.143 | <0.131 | <0.146 | <0.133 | 4.2 |
| C1-5 | 1.78 | 1.54 | 7.11 | 16.40 | <0.12 | 4.06 | <0.175 | 3.29 | <0.137 | <0.154 | <0.139 | <0.154 |
| C1-6 | 2.46 | 0.80 | <0.178 | 30.70 | <0.109 | 9.70 | <0.159 | 2.24 | <0.125 | 1.48 | <0.126 | <0.14 |
| C1-7 | <0.218 | <0.136 | <0.127 | 32.4 | <0.0779 | 10.0 | <0.114 | 1.55 | <0.0893 | <0.1 | <0.0906 | 27.20 |
| C1-8 | <0.302 | <0.189 | <0.177 | 46.6 | <0.108 | 15.7 | 2.34 | 3.91 | 1.69 | 6.43 | <0.126 | 3.47 |
| C1-9 | <0.272 | 1.2 | <0.159 | 27.2 | <0.0971 | 9.6 | 1.86 | 2.66 | 6.46 | 1.72 | <0.113 | 1.59 |
| C1-10 | <0.261 | <0.163 | <0.153 | 23.6 | <0.0933 | 9.2 | <0.137 | 1.27 | 5.42 | <0.12 | <0.109 | 1.62 |
| C1-11 | <0.27 | <0.169 | <0.158 | 2.19 | <0.0966 | <0.12 | <0.142 | 3.72 | <0.111 | <0.124 | <0.112 | <0.124 |
| C1-12 | <0.633 | <0.395 | <0.37 | 39.4 | <0.226 | <0.28 | <0.331 | <0.283 | <0.259 | <0.29 | <0.263 | <0.291 |
| C1-13 | <0.36 | <0.225 | <0.21 | 5.8 | <0.128 | <0.159 | <0.188 | <0.161 | <0.147 | <0.165 | <0.15 | <0.165 |
| C1-14 | <0.362 | <0.226 | <0.211 | 42.1 | <0.129 | 4.0 | <0.189 | <0.162 | <0.148 | <0.166 | <0.15 | <0.166 |
| C1-15 | <0.411 | <0.257 | <0.24 | 21.40 | <0.147 | 2.85 | <0.215 | <0.184 | <0.169 | <0.189 | <0.171 | <0.189 |
| U1-1 | <0.192 | 0.32 | <0.112 | 2.94 | <0.0685 | 0.74 | <0.1 | <0.0859 | <0.0786 | <0.088 | <0.0797 | <0.0881 |
| U1-2 | <0.234 | <0.146 | <0.137 | 1.28 | <0.0836 | 0.34 | <0.122 | 0.16 | 0.79 | <0.107 | <0.0972 | <0.108 |
| U1-3 | <0.326 | 0.47 | <0.191 | 5.10 | <0.116 | 1.48 | <0.171 | 0.56 | 3.66 | <0.15 | <0.136 | <0.15 |
| U1-4 | <0.213 | <0.133 | <0.124 | 2.22 | <0.0759 | 0.78 | 0.21 | 0.28 | 0.90 | 0.33 | <0.0883 | 0.25 |
| U1-5 | <0.335 | <0.209 | <0.196 | 4.89 | <0.12 | 0.95 | <0.175 | 0.40 | 2.20 | <0.154 | <0.139 | 2.48 |
| U1-6 | <0.343 | <0.214 | <0.2 | 78.7 | 1.2 | 34.8 | 3.3 | 4.1 | 29.4 | 6.9 | <0.142 | 14.5 |
| U1-7 | <0.279 | <0.174 | <0.163 | 42.4 | 1.0 | 23.4 | <0.146 | 1.8 | 9.4 | 1.2 | <0.116 | <0.128 |
| U1-8 | <0.33 | 2.0 | <0.193 | 109.0 | 3.3 | 45.9 | 2.0 | 5.8 | 30.4 | 6.1 | <0.137 | 2.5 |
| U1-9 | <0.22 | 2.1 | <0.128 | 181.0 | 6.9 | 78.7 | <0.115 | 5.4 | 3.9 | 8.4 | <0.0914 | <0.101 |
| U1-10 | 15.20 | <0.18 | <0.169 | 32.20 | <0.103 | 13.90 | 1.16 | 1.50 | 26.40 | 1.88 | <0.12 | 0.94 |
| U1-11 | <0.914 | 1.3 | <0.534 | 3.5 | <0.327 | <0.404 | <0.478 | <0.409 | <0.375 | <0.419 | <0.38 | <0.42 |
| U1-12 | <0.948 | <0.592 | <0.554 | 4.2 | <0.339 | <0.419 | <0.496 | <0.425 | <0.388 | <0.435 | <0.394 | <0.436 |
| U1-13 | <1.7 | <1.06 | <0.993 | 3.68 | <0.607 | <0.752 | <0.89 | <0.761 | <0.697 | <0.78 | <0.707 | <0.781 |
| U1-14 | <0.859 | <0.536 | <0.502 | 3.8 | <0.307 | <0.38 | <0.449 | <0.385 | <0.352 | 4.4 | <0.357 | <0.395 |
| U1-15 | <1.24 | <0.773 | <0.723 | 36.8 | <0.442 | <0.548 | <0.648 | <0.554 | <0.507 | <0.568 | <0.515 | <0.569 |
| M2-1 | <0.228 | 0.70 | <0.133 | 7.43 | <0.0813 | 2.64 | <0.119 | 0.81 | 0.21 | 1.11 | <0.0946 | <0.105 |
| M2-2 | <0.326 | 1.06 | <0.19 | 7.52 | <0.116 | 2.29 | <0.17 | 0.80 | 1.45 | 0.79 | <0.135 | 0.78 |
| M2-3 | <0.295 | 0.59 | <0.173 | 15.90 | <0.106 | 5.37 | 1.26 | 2.47 | 1.03 | 1.18 | <0.123 | 2.12 |
| M2-4 | <0.357 | 3.3 | <0.209 | 91.6 | <0.128 | 35.5 | 4.6 | 15.1 | 1.5 | 7.5 | <0.148 | 14.9 |
| M2-5 | <0.222 | 0.7 | <0.13 | 11.8 | <0.0792 | 4.4 | 1.2 | 0.6 | 0.5 | 2.6 | <0.0922 | 1.9 |
| M2-6 | <0.259 | <0.161 | <0.151 | 33.9 | 9.5 | 7.6 | <0.135 | 8.0 | 7.6 | 3.5 | <0.108 | 1.9 |
| M2-7 | <1.03 | <0.642 | <0.601 | 103.0 | <0.367 | 43.5 | <0.538 | 13.6 | 23.7 | 3.7 | <0.427 | 10.4 |
| M2-8 | <0.289 | <0.18 | <0.169 | 50.2 | <0.103 | 15.8 | <0.151 | 2.6 | <0.118 | <0.133 | <0.12 | <0.133 |
| M2-9 | <0.296 | 2.1 | <0.173 | 107.0 | <0.106 | 51.9 | <0.155 | 18.6 | 11.8 | 6.4 | <0.123 | 4.9 |
| M2-10 | <0.335 | 1.27 | <0.196 | 26.00 | <0.12 | 9.73 | <0.175 | 4.38 | 8.20 | 1.06 | <0.139 | 1.65 |
| M2-11 | <1.28 | 1.6 | <0.749 | 5.7 | <0.458 | <0.567 | <0.671 | <0.574 | <0.525 | <0.588 | <0.533 | <0.589 |
| M2-12 | <0.415 | <0.259 | <0.243 | 4.75 | <0.148 | 0.57 | <0.217 | <0.186 | <0.17 | <0.191 | <0.173 | <0.191 |
| M2-13 | <0.538 | <0.336 | <0.314 | 11.5 | <0.192 | 3.5 | <0.282 | <0.241 | <0.22 | <0.247 | <0.224 | <0.247 |
| M2-14 | <0.318 | <0.199 | <0.186 | 38.8 | <0.114 | 2.9 | <0.167 | <0.143 | <0.13 | <0.146 | <0.132 | <0.146 |
| M2-15 | <0.334 | <0.209 | <0.195 | 12.7 | 4.0 | 1.6 | <0.175 | <0.15 | <0.137 | <0.153 | <0.139 | <0.154 |
| C2-1 | <0.22 | <0.137 | <0.128 | 10.9 | <0.0785 | 5.6 | <0.115 | <0.0984 | 2.6 | 1.1 | <0.0914 | <0.101 |
| C2-2 | <0.199 | 0.26 | <0.116 | 3.42 | <0.0712 | 1.25 | <0.104 | 0.47 | 0.15 | 0.35 | <0.0828 | <0.0916 |
| C2-3 | <0.236 | 0.5 | <0.138 | 11.1 | <0.0844 | 3.7 | 0.7 | 1.3 | 2.6 | 0.9 | <0.0981 | 0.6 |
| C2-4 | <0.726 | <0.454 | <0.425 | 8.4 | <0.26 | <0.321 | <0.38 | 2.4 | <0.298 | <0.333 | <0.302 | <0.334 |
| C2-5 | <0.211 | <0.132 | <0.123 | 3.44 | <0.0753 | 1.26 | <0.11 | 0.43 | 0.42 | <0.0967 | <0.0876 | <0.0969 |
| C2-6 | <0.258 | <0.161 | <0.151 | 13.3 | <0.092 | <0.114 | <0.135 | 0.9 | 3.2 | <0.118 | <0.107 | <0.118 |
| C2-7 | <0.199 | <0.124 | <0.116 | 8.86 | <0.0712 | <4.6 | <0.104 | <0.0892 | 2.98 | 2.46 | <0.0828 | <0.0916 |
| C2-8 | <0.272 | 1.2 | 5.5 | 18.8 | <0.097 | 6.3 | <0.142 | 2.3 | 9.1 | 1.6 | <0.113 | 1.4 |
| C2-9 | <0.304 | <0.19 | <0.177 | 40.0 | <0.108 | 19.9 | <0.159 | 8.5 | <0.124 | <0.139 | <0.126 | <0.14 |
| C2-10 | <0.26 | 0.9 | 11.4 | 45.6 | <0.0931 | 4.2 | <0.136 | 1.0 | 0.6 | <0.12 | <0.108 | <0.12 |
| C2-11 | <0.259 | 1.0 | <0.151 | 3.7 | <0.0924 | <0.114 | <0.135 | <0.116 | <0.106 | 4.8 | <0.108 | <0.119 |
| C2-12 | <0.29 | <0.181 | <0.17 | 3.7 | <0.104 | <0.128 | <0.152 | <0.13 | <0.119 | <0.133 | <0.121 | <0.133 |
| C2-13 | <0.375 | 1.0 | <0.219 | 2.5 | <0.134 | <0.166 | <0.196 | <0.168 | <0.154 | <0.172 | <0.156 | <0.172 |
| C2-14 | <0.302 | 0.86 | <0.177 | 3.24 | <0.108 | <0.134 | <0.158 | <0.135 | <0.124 | <0.139 | <0.126 | <0.139 |
| C2-15 | <0.315 | 2.21 | <0.184 | 6.08 | <0.113 | <0.139 | <0.165 | <0.141 | <0.129 | <0.145 | <0.131 | <0.145 |
| U2-1 | <0.347 | <0.217 | <0.203 | 4.40 | <0.124 | 1.25 | <0.182 | 0.54 | 2.46 | <0.159 | <0.144 | <0.159 |
| U2-2 | <0.287 | 1.4 | <0.168 | 4.1 | <0.103 | 1.5 | <0.15 | <0.129 | 1.6 | <0.132 | <0.119 | <0.132 |
| U2-3 | <0.303 | <0.189 | <0.177 | 13.90 | <0.108 | 3.02 | <0.159 | 0.98 | <0.124 | <0.139 | <0.126 | 1.28 |
| U2-4 | <0.302 | <0.189 | <0.177 | 5.67 | <0.108 | 1.86 | <0.158 | 0.63 | 3.07 | <0.139 | <0.126 | 0.49 |
| U2-5 | <0.214 | <0.134 | <0.125 | 59.60 | 48.70 | 23.80 | <0.112 | 0.85 | 3.38 | <0.0983 | <0.0891 | <0.0985 |
| U2-6 | <0.278 | <0.174 | <0.163 | 9.17 | <0.0995 | 2.99 | 0.60 | <0.125 | 1.51 | <0.128 | <0.116 | <0.128 |
| U2-7 | 3.44 | <0.216 | <0.202 | 8.42 | <0.124 | 4.19 | <0.181 | 0.72 | <0.142 | <0.159 | <0.144 | <0.159 |
| U2-8 | 0.8 | 0.9 | <0.167 | 5.2 | <0.102 | 2.5 | <0.15 | 0.8 | <0.117 | <0.131 | <0.119 | <0.131 |
| U2-9 | <0.353 | 0.81 | <0.206 | 4.07 | <0.126 | 0.67 | <0.185 | <0.158 | 1.53 | <0.162 | <0.147 | <0.162 |
| U2-10 | <0.276 | <0.172 | <0.161 | 3.43 | <0.0987 | 1.07 | <0.145 | 0.46 | 1.21 | <0.127 | <0.115 | <0.127 |
| U2-11 | <0.366 | <0.228 | <0.214 | 2.20 | <0.131 | 1.43 | <0.192 | <0.164 | <0.15 | <0.168 | <0.152 | <0.168 |
| U2-12 | <0.44 | <0.275 | <0.257 | 3.4 | <0.157 | 1.3 | <0.231 | <0.197 | <0.18 | <0.202 | <0.183 | <0.202 |
| U2-13 | <0.693 | <0.433 | <0.405 | 3.5 | <0.248 | <0.307 | 2.5 | <0.31 | <0.284 | <0.318 | <0.288 | <0.318 |
| U2-14 | <0.488 | <0.304 | <0.285 | 6.2 | <0.174 | <0.216 | <0.255 | <0.218 | 1.7 | <0.224 | <0.203 | <0.224 |
| U2-15 | <0.367 | <0.229 | <0.215 | 3.5 | <0.131 | <0.163 | <0.192 | <0.165 | <0.151 | <0.169 | <0.153 | <0.169 |

**TABLE S3 (d): Individual results of dl-PCB in the fish samples (n=90, dl-PCB, ng/g fat).**

| **ID** | **PCB 81** | **PCB 77** | **PCB 123** | **PCB 118** | **PCB 114** | **PCB 105** | **PCB 126** | **PCB 167** | **PCB 156** | **PCB 157** | **PCB 169** | **PCB 189** |
| --- | --- | --- | --- | --- | --- | --- | --- | --- | --- | --- | --- | --- |
| M1-1 | <0.00489 | <0.00306 | <0.00286 | 0.478 | <0.00175 | <0.00217 | <0.00256 | <0.00219 | 0.115 | <0.00225 | <0.00203 | <0.00225 |
| M1-2 | <0.00706 | <0.00441 | <0.00413 | 0.624 | <0.00252 | 0.087 | <0.0037 | <0.00316 | <0.00289 | <0.00324 | <0.00294 | <0.00325 |
| M1-3 | <0.03 | <0.0187 | <0.0175 | 0.282 | <0.0107 | <0.0133 | <0.0157 | <0.0134 | <0.0123 | <0.0138 | <0.0125 | <0.0138 |
| M1-4 | <0.0567 | <0.0354 | <0.0331 | 0.917 | <0.0202 | <0.0251 | <0.0297 | <0.0254 | <0.0232 | <0.026 | <0.0236 | <0.026 |
| M1-5 | <0.0037 | <0.00231 | <0.00216 | 0.099 | <0.00132 | <0.00163 | <0.00193 | <0.00166 | 0.026 | <0.0017 | <0.00154 | <0.0017 |
| M1-6 | <0.0192 | <0.012 | <0.0112 | 0.594 | <0.00685 | 0.227 | <0.01 | <0.00859 | <0.00786 | <0.0088 | <0.00797 | <0.00881 |
| M1-7 | <0.0472 | <0.0295 | <0.0276 | 1.470 | <0.0169 | 0.469 | <0.0247 | <0.0211 | <0.0194 | <0.0217 | <0.0196 | <0.0217 |
| M1-8 | <0.00914 | 0.541 | <0.00534 | 9.320 | 0.721 | 5.940 | 0.136 | 0.699 | 0.349 | 0.245 | <0.0038 | 0.249 |
| M1-9 | <0.00313 | <0.00195 | <0.00183 | 1.590 | <0.00112 | 0.648 | <0.00164 | <0.0014 | <0.00128 | <0.00143 | <0.0013 | <0.00144 |
| M1-10 | <0.00167 | <0.00104 | <0.000974 | 0.102 | <0.000595 | 0.022 | 0.016 | 0.008 | 0.041 | <0.000765 | <0.000693 | <0.000766 |
| M1-11 | <0.134 | <0.0838 | <0.0784 | 1.640 | <0.0479 | <0.0594 | 0.377 | <0.0601 | <0.055 | <0.0616 | <0.0558 | <0.0617 |
| M1-12 | <0.121 | <0.0758 | <0.071 | <0.0491 | <0.0434 | <0.0537 | <0.0636 | <0.0544 | <0.0498 | <0.0557 | <0.0505 | <0.0558 |
| M1-13 | <0.0638 | <0.0398 | <0.0373 | 0.364 | <0.0228 | <0.0282 | <0.0334 | <0.0285 | <0.0261 | <0.0292 | <0.0265 | <0.0293 |
| M1-14 | <0.051 | <0.0318 | <0.0298 | 0.271 | <0.0182 | <0.0226 | <0.0267 | <0.0228 | 0.062 | <0.0234 | <0.0212 | <0.0234 |
| M1-15 | <0.218 | <0.136 | <0.127 | 0.692 | <0.0779 | <0.0964 | <0.114 | <0.0976 | <0.0893 | <0.1 | <0.0906 | <0.1 |
| C1-1 | <0.00506 | 0.037 | <0.00296 | 1.490 | <0.00181 | 0.466 | <0.00265 | 0.208 | 0.139 | 0.072 | <0.0021 | <0.00233 |
| C1-2 | <0.00322 | <0.00201 | <0.00188 | 0.598 | <0.00115 | 0.257 | <0.00169 | <0.00144 | 0.104 | <0.00148 | <0.00134 | <0.00148 |
| C1-3 | <0.00712 | <0.00445 | 0.444 | 2.120 | <0.00254 | 0.556 | <0.00373 | 0.154 | 0.152 | 0.098 | <0.00296 | 0.102 |
| C1-4 | <0.00775 | <0.00484 | <0.00453 | 0.258 | <0.00277 | <0.00343 | <0.00406 | <0.00347 | <0.00318 | <0.00356 | <0.00322 | 0.102 |
| C1-5 | 0.066 | 0.057 | 0.263 | 0.608 | <0.00442 | 0.150 | <0.00648 | 0.122 | <0.00507 | <0.00568 | <0.00515 | <0.00569 |
| C1-6 | 0.045 | 0.015 | <0.00327 | 0.566 | <0.002 | 0.179 | <0.00293 | 0.041 | <0.0023 | 0.027 | <0.00233 | <0.00258 |
| C1-7 | <0.00352 | <0.0022 | <0.00206 | 0.523 | <0.00126 | 0.161 | <0.00184 | 0.025 | <0.00144 | <0.00161 | <0.00146 | 0.438 |
| C1-8 | <0.0085 | <0.00531 | <0.00497 | 1.310 | <0.00304 | 0.441 | 0.066 | 0.110 | 0.048 | 0.181 | <0.00353 | 0.098 |
| C1-9 | <0.00639 | 0.027 | <0.00373 | 0.640 | <0.00228 | 0.226 | 0.044 | 0.063 | 0.152 | 0.040 | <0.00266 | 0.037 |
| C1-10 | <0.00914 | <0.00571 | <0.00534 | 0.825 | <0.00327 | 0.323 | <0.00478 | 0.044 | 0.190 | <0.00419 | <0.0038 | 0.057 |
| C1-11 | <0.00981 | <0.00612 | <0.00573 | 0.079 | <0.0035 | <0.00434 | <0.00513 | 0.135 | <0.00402 | <0.0045 | <0.00408 | <0.00451 |
| C1-12 | <0.00861 | <0.00538 | <0.00503 | 0.536 | <0.00308 | <0.00381 | <0.00451 | <0.00386 | <0.00353 | <0.00395 | <0.00358 | <0.00396 |
| C1-13 | <0.00746 | <0.00465 | <0.00436 | 0.120 | <0.00266 | <0.0033 | <0.0039 | <0.00334 | <0.00306 | <0.00342 | <0.0031 | <0.00343 |
| C1-14 | <0.00563 | <0.00351 | <0.00329 | 0.656 | <0.00201 | 0.063 | <0.00295 | <0.00252 | <0.00231 | <0.00258 | <0.00234 | <0.00259 |
| C1-15 | <0.00718 | <0.00448 | <0.0042 | 0.374 | <0.00257 | 0.050 | <0.00376 | <0.00322 | <0.00294 | <0.0033 | <0.00299 | <0.0033 |
| U1-1 | <0.0255 | 0.043 | <0.0149 | 0.392 | <0.00911 | 0.098 | <0.0134 | <0.0114 | <0.0104 | <0.0117 | <0.0106 | <0.0117 |
| U1-2 | <0.0319 | <0.0199 | <0.0186 | 0.175 | <0.0114 | 0.046 | <0.0167 | 0.022 | 0.108 | <0.0146 | <0.0132 | <0.0147 |
| U1-3 | <0.0359 | 0.051 | <0.021 | 0.562 | <0.0128 | 0.163 | <0.0188 | 0.062 | 0.403 | <0.0165 | <0.0149 | <0.0165 |
| U1-4 | <0.00934 | <0.00583 | <0.00546 | 0.097 | <0.00334 | 0.034 | 0.009 | 0.012 | 0.040 | 0.015 | <0.00388 | 0.011 |
| U1-5 | <0.0411 | <0.0257 | <0.024 | 0.601 | <0.0147 | 0.117 | <0.0215 | 0.049 | 0.271 | <0.0189 | <0.0171 | 0.305 |
| U1-6 | <0.0671 | <0.0419 | <0.0392 | 15.400 | 0.243 | 6.810 | 0.636 | 0.806 | 5.760 | 1.350 | <0.0279 | 2.830 |
| U1-7 | <0.0879 | <0.0549 | <0.0514 | 13.400 | 0.301 | 7.370 | <0.046 | 0.581 | 2.970 | 0.389 | <0.0366 | <0.0404 |
| U1-8 | <0.034 | 0.206 | <0.0199 | 11.300 | 0.344 | 4.730 | 0.205 | 0.593 | 3.140 | 0.631 | <0.0141 | 0.256 |
| U1-9 | <0.00977 | 0.093 | <0.00571 | 8.060 | 0.307 | 3.500 | <0.00511 | 0.240 | 0.175 | 0.372 | <0.00406 | <0.00449 |
| U1-10 | 0.904 | <0.0107 | <0.01 | 1.910 | <0.00611 | 0.823 | 0.069 | 0.089 | 1.570 | 0.111 | <0.00711 | 0.056 |
| U1-11 | <0.0531 | 0.073 | <0.031 | 0.203 | <0.019 | <0.0235 | <0.0278 | <0.0238 | <0.0218 | <0.0244 | <0.0221 | <0.0244 |
| U1-12 | <0.0354 | <0.0221 | <0.0207 | 0.156 | <0.0127 | <0.0157 | <0.0185 | <0.0159 | <0.0145 | <0.0163 | <0.0147 | <0.0163 |
| U1-13 | <0.162 | <0.101 | <0.0949 | 0.351 | <0.058 | <0.0718 | <0.085 | <0.0727 | <0.0666 | <0.0745 | <0.0675 | <0.0746 |
| U1-14 | <0.097 | <0.0605 | <0.0567 | 0.427 | <0.0346 | <0.0429 | <0.0508 | <0.0434 | <0.0397 | 0.492 | <0.0403 | <0.0446 |
| U1-15 | <0.085 | <0.0531 | <0.0497 | 2.530 | <0.0304 | <0.0376 | <0.0445 | <0.0381 | <0.0348 | <0.039 | <0.0353 | <0.0391 |
| M2-1 | <0.00864 | 0.027 | <0.00505 | 0.282 | <0.00309 | 0.100 | <0.00453 | 0.031 | 0.008 | 0.042 | <0.00359 | <0.00397 |
| M2-2 | <0.0123 | 0.040 | <0.0072 | 0.284 | <0.0044 | 0.087 | <0.00645 | 0.030 | 0.055 | 0.030 | <0.00512 | 0.030 |
| M2-3 | <0.00898 | 0.018 | <0.00525 | 0.483 | <0.00321 | 0.163 | 0.038 | 0.075 | 0.031 | 0.036 | <0.00373 | 0.065 |
| M2-4 | <0.00531 | 0.049 | <0.0031 | 1.360 | <0.0019 | 0.527 | 0.068 | 0.224 | 0.022 | 0.111 | <0.00221 | 0.222 |
| M2-5 | <0.0106 | 0.032 | <0.00621 | 0.567 | <0.0038 | 0.210 | 0.058 | 0.030 | 0.024 | 0.125 | <0.00442 | 0.089 |
| M2-6 | <0.00238 | <0.00149 | <0.00139 | 0.313 | 0.087 | 0.070 | <0.00125 | 0.074 | 0.070 | 0.032 | <0.000991 | 0.017 |
| M2-7 | <0.0102 | <0.00634 | <0.00594 | 1.020 | <0.00363 | 0.430 | <0.00532 | 0.135 | 0.234 | 0.037 | <0.00422 | 0.102 |
| M2-8 | <0.00478 | <0.00298 | <0.00279 | 0.830 | <0.00171 | 0.261 | <0.0025 | 0.044 | <0.00196 | <0.00219 | <0.00199 | <0.00219 |
| M2-9 | <0.0025 | 0.017 | <0.00146 | 0.904 | <0.000893 | 0.439 | <0.00131 | 0.157 | 0.100 | 0.054 | <0.00104 | 0.041 |
| M2-10 | <0.00601 | 0.023 | <0.00351 | 0.467 | <0.00215 | 0.175 | <0.00315 | 0.079 | 0.147 | 0.019 | <0.0025 | 0.030 |
| M2-11 | <0.0876 | 0.111 | <0.0512 | 0.389 | <0.0313 | <0.0388 | <0.0459 | <0.0392 | <0.0359 | <0.0402 | <0.0364 | <0.0403 |
| M2-12 | <0.0455 | <0.0284 | <0.0266 | 0.521 | <0.0163 | 0.063 | <0.0238 | <0.0204 | <0.0187 | <0.0209 | <0.0189 | <0.0209 |
| M2-13 | <0.0213 | <0.0133 | <0.0124 | 0.453 | <0.00759 | 0.140 | <0.0111 | <0.00952 | <0.00871 | <0.00975 | <0.00883 | <0.00977 |
| M2-14 | <0.00685 | <0.00428 | <0.00401 | 0.836 | <0.00245 | 0.063 | <0.00359 | <0.00307 | <0.00281 | <0.00315 | <0.00285 | <0.00315 |
| M2-15 | <0.0159 | <0.00995 | <0.00931 | 0.607 | 0.191 | 0.074 | <0.00834 | <0.00714 | <0.00653 | <0.00731 | <0.00662 | <0.00733 |
| C2-1 | <0.00646 | <0.00403 | <0.00377 | 0.319 | <0.00231 | 0.163 | <0.00338 | <0.00289 | 0.077 | 0.033 | <0.00268 | <0.00297 |
| C2-2 | <0.00823 | 0.011 | <0.00481 | 0.141 | <0.00294 | 0.052 | <0.00431 | 0.019 | 0.006 | 0.015 | <0.00342 | <0.00378 |
| C2-3 | <0.0178 | 0.036 | <0.0104 | 0.840 | <0.00637 | 0.282 | 0.049 | 0.096 | 0.192 | 0.070 | <0.00741 | 0.047 |
| C2-4 | <0.112 | <0.0698 | <0.0654 | 1.290 | <0.04 | <0.0495 | <0.0586 | 0.370 | <0.0458 | <0.0513 | <0.0465 | <0.0514 |
| C2-5 | <0.00911 | <0.00569 | <0.00532 | 0.149 | <0.00325 | 0.054 | <0.00477 | 0.019 | 0.018 | <0.00418 | <0.00379 | <0.00419 |
| C2-6 | <0.00234 | <0.00146 | <0.00137 | 0.121 | <0.000836 | <0.00103 | <0.00122 | 0.008 | 0.029 | <0.00107 | <0.000972 | <0.00108 |
| C2-7 | <0.00531 | <0.00332 | <0.0031 | 0.236 | <0.0019 | <0.123 | <0.00278 | <0.00238 | 0.080 | 0.066 | <0.00221 | <0.00244 |
| C2-8 | <0.00996 | 0.046 | 0.203 | 0.691 | <0.00356 | 0.230 | <0.00521 | 0.082 | 0.333 | 0.060 | <0.00414 | 0.050 |
| C2-9 | <0.00338 | <0.00211 | <0.00197 | 0.445 | <0.00121 | 0.222 | <0.00177 | 0.095 | <0.00138 | <0.00155 | <0.0014 | <0.00155 |
| C2-10 | <0.00328 | 0.012 | 0.143 | 0.574 | <0.00117 | 0.052 | <0.00172 | 0.013 | 0.007 | <0.0015 | <0.00136 | <0.00151 |
| C2-11 | <0.00901 | 0.033 | <0.00527 | 0.130 | <0.00322 | <0.00399 | <0.00472 | <0.00404 | <0.00369 | 0.169 | <0.00375 | <0.00414 |
| C2-12 | <0.0129 | <0.00808 | <0.00756 | 0.166 | <0.00462 | <0.00573 | <0.00678 | <0.0058 | <0.0053 | <0.00594 | <0.00538 | <0.00595 |
| C2-13 | <0.0121 | 0.033 | <0.0071 | 0.081 | <0.00434 | <0.00537 | <0.00636 | <0.00544 | <0.00498 | <0.00557 | <0.00505 | <0.00558 |
| C2-14 | <0.0124 | 0.035 | <0.00723 | 0.133 | <0.00442 | <0.00548 | <0.00648 | <0.00554 | <0.00507 | <0.00568 | <0.00515 | <0.00569 |
| C2-15 | <0.015 | 0.105 | <0.00876 | 0.289 | <0.00536 | <0.00664 | <0.00785 | <0.00672 | <0.00615 | <0.00688 | <0.00624 | <0.00689 |
| U2-1 | <0.05 | <0.0312 | <0.0292 | 0.634 | <0.0179 | 0.180 | <0.0262 | 0.077 | 0.354 | <0.0229 | <0.0208 | <0.023 |
| U2-2 | <0.0638 | 0.316 | <0.0373 | 0.902 | <0.0228 | 0.337 | <0.0334 | <0.0285 | 0.347 | <0.0292 | <0.0265 | <0.0293 |
| U2-3 | <0.0224 | <0.014 | <0.0131 | 1.030 | <0.00799 | 0.223 | <0.0117 | 0.072 | <0.00917 | <0.0103 | <0.0093 | 0.095 |
| U2-4 | <0.0773 | <0.0482 | <0.0452 | 1.450 | <0.0276 | 0.475 | <0.0405 | 0.160 | 0.785 | <0.0355 | <0.0321 | 0.125 |
| U2-5 | <0.00864 | <0.0054 | <0.00505 | 2.400 | 1.970 | 0.959 | <0.00453 | 0.034 | 0.136 | <0.00397 | <0.00359 | <0.00397 |
| U2-6 | <0.0107 | <0.00669 | <0.00626 | 0.353 | <0.00383 | 0.115 | 0.023 | <0.0048 | 0.058 | <0.00492 | <0.00445 | <0.00492 |
| U2-7 | 0.154 | <0.00971 | <0.00909 | 0.378 | <0.00555 | 0.188 | <0.00814 | 0.032 | <0.00637 | <0.00713 | <0.00646 | <0.00715 |
| U2-8 | 0.027 | 0.030 | <0.0054 | 0.167 | <0.0033 | 0.081 | <0.00484 | 0.025 | <0.00379 | <0.00424 | <0.00384 | <0.00425 |
| U2-9 | <0.0234 | 0.054 | <0.0137 | 0.269 | <0.00836 | 0.044 | <0.0122 | <0.0105 | 0.101 | <0.0107 | <0.00972 | <0.0108 |
| U2-10 | <0.0161 | <0.0101 | <0.00943 | 0.200 | <0.00577 | 0.063 | <0.00845 | 0.027 | 0.071 | <0.00741 | <0.00671 | <0.00742 |
| U2-11 | <0.0481 | <0.03 | <0.0281 | 0.290 | <0.0172 | 0.188 | <0.0252 | <0.0215 | <0.0197 | <0.0221 | <0.02 | <0.0221 |
| U2-12 | <0.0243 | <0.0152 | <0.0142 | 0.188 | <0.00868 | 0.071 | <0.0127 | <0.0109 | <0.00995 | <0.0111 | <0.0101 | <0.0112 |
| U2-13 | <0.17 | <0.106 | <0.0993 | 0.863 | <0.0607 | <0.0752 | 0.612 | <0.0761 | <0.0697 | <0.078 | <0.0707 | <0.0781 |
| U2-14 | <0.213 | <0.133 | <0.124 | 2.680 | <0.0759 | <0.094 | <0.111 | <0.0952 | 0.740 | <0.0975 | <0.0883 | <0.0977 |
| U2-15 | <0.0196 | <0.0122 | <0.0115 | 0.187 | <0.00701 | <0.00868 | <0.0103 | <0.00878 | <0.00804 | <0.009 | <0.00815 | <0.00902 |

**TABLE S3 (e): TEQ_PCB_ in the fish samples. Congeners in which the result was <MQL were replaced by 1/2MQL (n=90, pg⋅TEQ/g w.w).**

| **ID** | **PCB-77** | **PCB-81** | **PCB-105** | **PCB-114** | **PCB-118** | **PCB-123** | **PCB-126** | **PCB-156** | **PCB-157** | **PCB-167** | **PCB-169** | **PCB-189** | **TEQ_PCB** |
| --- | --- | --- | --- | --- | --- | --- | --- | --- | --- | --- | --- | --- | --- |
| M1-1 | 0.000008 | 0.000037 | 0.000002 | 0.000001 | 0.000718 | 0.000002 | 0.006418 | 0.000173 | 0.000002 | 0.000002 | 0.001529 | 0.000002 | 0.00889307 |
| M1-2 | 0.000009 | 0.000043 | 0.000107 | 0.000002 | 0.000763 | 0.000003 | 0.007534 | 0.000002 | 0.000002 | 0.000002 | 0.001795 | 0.000002 | 0.01026138 |
| M1-3 | 0.00001 | 0.00005 | 0.000002 | 0.000002 | 0.000094 | 0.000003 | 0.008703 | 0.000002 | 0.000002 | 0.000002 | 0.002073 | 0.000002 | 0.01094563 |
| M1-4 | 0.000009 | 0.000045 | 0.000002 | 0.000002 | 0.000144 | 0.000003 | 0.007771 | 0.000002 | 0.000002 | 0.000002 | 0.001851 | 0.000002 | 0.00983364 |
| M1-5 | 0.000009 | 0.000044 | 0.000002 | 0.000002 | 0.000236 | 0.000003 | 0.007681 | 0.000063 | 0.000002 | 0.000002 | 0.00183 | 0.000002 | 0.00987497 |
| M1-6 | 0.00001 | 0.00005 | 0.000119 | 0.000002 | 0.000311 | 0.000003 | 0.00876 | 0.000002 | 0.000002 | 0.000002 | 0.002087 | 0.000002 | 0.01135106 |
| M1-7 | 0.00001 | 0.000048 | 0.000096 | 0.000002 | 0.0003 | 0.000003 | 0.008428 | 0.000002 | 0.000002 | 0.000002 | 0.002008 | 0.000002 | 0.01090337 |
| M1-8 | 0.001684 | 0.000043 | 0.005545 | 0.000674 | 0.008703 | 0.000002 | 0.421933 | 0.000326 | 0.000229 | 0.000653 | 0.001775 | 0.000233 | 0.44179889 |
| M1-9 | 0.000008 | 0.000041 | 0.001682 | 0.000001 | 0.004124 | 0.000002 | 0.007078 | 0.000002 | 0.000002 | 0.000002 | 0.001686 | 0.000002 | 0.01463045 |
| M1-10 | 0.00001 | 0.000046 | 0.00012 | 0.000002 | 0.000569 | 0.000003 | 0.295326 | 0.000225 | 0.000002 | 0.000043 | 0.001925 | 0.000002 | 0.29827175 |
| M1-11 | 0.00001 | 0.000047 | 0.000002 | 0.000002 | 0.000114 | 0.000003 | 0.08741 | 0.000002 | 0.000002 | 0.000002 | 0.001941 | 0.000002 | 0.08953683 |
| M1-12 | 0.00004 | 0.000193 | 0.000009 | 0.000007 | 0.000008 | 0.000011 | 0.033712 | 0.000008 | 0.000009 | 0.000009 | 0.00803 | 0.000009 | 0.04204467 |
| M1-13 | 0.000031 | 0.000151 | 0.000007 | 0.000005 | 0.000172 | 0.000009 | 0.02628 | 0.000006 | 0.000007 | 0.000007 | 0.00626 | 0.000007 | 0.03294075 |
| M1-14 | 0.000045 | 0.000217 | 0.00001 | 0.000008 | 0.000231 | 0.000013 | 0.037926 | 0.000053 | 0.00001 | 0.00001 | 0.009034 | 0.00001 | 0.04756673 |
| M1-15 | 0.000021 | 0.000099 | 0.000004 | 0.000004 | 0.000063 | 0.000006 | 0.017248 | 0.000004 | 0.000005 | 0.000004 | 0.004109 | 0.000005 | 0.02156998 |
| C1-1 | 0.000174 | 0.000036 | 0.000659 | 0.000001 | 0.002105 | 0.000002 | 0.006238 | 0.000197 | 0.000101 | 0.000293 | 0.001486 | 0.000002 | 0.01129386 |
| C1-2 | 0.000009 | 0.000043 | 0.000686 | 0.000002 | 0.001597 | 0.000003 | 0.0075 | 0.000278 | 0.000002 | 0.000002 | 0.001787 | 0.000002 | 0.01190944 |
| C1-3 | 0.000009 | 0.000043 | 0.000671 | 0.000002 | 0.002564 | 0.000536 | 0.0075 | 0.000183 | 0.000118 | 0.000186 | 0.001787 | 0.000124 | 0.01372087 |
| C1-4 | 0.00001 | 0.000048 | 0.000002 | 0.000002 | 0.000319 | 0.000003 | 0.008354 | 0.000002 | 0.000002 | 0.000002 | 0.00199 | 0.000126 | 0.01085991 |
| C1-5 | 0.000154 | 0.000533 | 0.000122 | 0.000002 | 0.000493 | 0.000213 | 0.00876 | 0.000002 | 0.000002 | 0.000099 | 0.002087 | 0.000002 | 0.01246947 |
| C1-6 | 0.00008 | 0.000739 | 0.000291 | 0.000002 | 0.000921 | 0.000003 | 0.007956 | 0.000002 | 0.000044 | 0.000067 | 0.001895 | 0.000002 | 0.01200199 |
| C1-7 | 0.000007 | 0.000033 | 0.000299 | 0.000001 | 0.000972 | 0.000002 | 0.005705 | 0.000001 | 0.000002 | 0.000047 | 0.001359 | 0.000815 | 0.00924209 |
| C1-8 | 0.000009 | 0.000045 | 0.000471 | 0.000002 | 0.001399 | 0.000003 | 0.233745 | 0.000051 | 0.000193 | 0.000117 | 0.001884 | 0.000104 | 0.23802258 |
| C1-9 | 0.000116 | 0.000041 | 0.000289 | 0.000001 | 0.000817 | 0.000002 | 0.185558 | 0.000194 | 0.000052 | 0.00008 | 0.001695 | 0.000048 | 0.18889184 |
| C1-10 | 0.000008 | 0.000039 | 0.000277 | 0.000001 | 0.000708 | 0.000002 | 0.006839 | 0.000163 | 0.000002 | 0.000038 | 0.001629 | 0.000049 | 0.00975512 |
| C1-11 | 0.000008 | 0.000041 | 0.000002 | 0.000001 | 0.000066 | 0.000002 | 0.007078 | 0.000002 | 0.000002 | 0.000112 | 0.001686 | 0.000002 | 0.00900176 |
| C1-12 | 0.00002 | 0.000095 | 0.000004 | 0.000003 | 0.001181 | 0.000006 | 0.016563 | 0.000004 | 0.000004 | 0.000004 | 0.003945 | 0.000004 | 0.02183464 |
| C1-13 | 0.000011 | 0.000054 | 0.000002 | 0.000002 | 0.000173 | 0.000003 | 0.009415 | 0.000002 | 0.000002 | 0.000002 | 0.002243 | 0.000002 | 0.01191243 |
| C1-14 | 0.000011 | 0.000054 | 0.000121 | 0.000002 | 0.001264 | 0.000003 | 0.009468 | 0.000002 | 0.000002 | 0.000002 | 0.002255 | 0.000002 | 0.01318917 |
| C1-15 | 0.000013 | 0.000062 | 0.000085 | 0.000002 | 0.000642 | 0.000004 | 0.010766 | 0.000003 | 0.000003 | 0.000003 | 0.002565 | 0.000003 | 0.01414958 |
| U1-1 | 0.000032 | 0.000029 | 0.000022 | 0.000001 | 0.000088 | 0.000002 | 0.005019 | 0.000001 | 0.000001 | 0.000001 | 0.001195 | 0.000001 | 0.00639376 |
| U1-2 | 0.000007 | 0.000035 | 0.00001 | 0.000001 | 0.000039 | 0.000002 | 0.006124 | 0.000024 | 0.000002 | 0.000005 | 0.001459 | 0.000002 | 0.00770869 |
| U1-3 | 0.000047 | 0.000049 | 0.000044 | 0.000002 | 0.000153 | 0.000003 | 0.008536 | 0.00011 | 0.000002 | 0.000017 | 0.002033 | 0.000002 | 0.01099787 |
| U1-4 | 0.000007 | 0.000032 | 0.000023 | 0.000001 | 0.000066 | 0.000002 | 0.021428 | 0.000027 | 0.00001 | 0.000008 | 0.001325 | 0.000007 | 0.02293671 |
| U1-5 | 0.00001 | 0.00005 | 0.000028 | 0.000002 | 0.000147 | 0.000003 | 0.00876 | 0.000066 | 0.000002 | 0.000012 | 0.002087 | 0.000074 | 0.01124192 |
| U1-6 | 0.000011 | 0.000051 | 0.001044 | 0.000037 | 0.002361 | 0.000003 | 0.32507 | 0.000882 | 0.000207 | 0.000123 | 0.002137 | 0.000434 | 0.33236169 |
| U1-7 | 0.000009 | 0.000042 | 0.000701 | 0.000029 | 0.001273 | 0.000002 | 0.007295 | 0.000282 | 0.000037 | 0.000055 | 0.001738 | 0.000002 | 0.01146433 |
| U1-8 | 0.000199 | 0.000049 | 0.001376 | 0.0001 | 0.003276 | 0.000003 | 0.198877 | 0.000913 | 0.000184 | 0.000173 | 0.002057 | 0.000075 | 0.20728213 |
| U1-9 | 0.000209 | 0.000033 | 0.00236 | 0.000207 | 0.005439 | 0.000002 | 0.005754 | 0.000118 | 0.000251 | 0.000162 | 0.001371 | 0.000002 | 0.01590843 |
| U1-10 | 0.000009 | 0.004574 | 0.000417 | 0.000002 | 0.000966 | 0.000003 | 0.116266 | 0.000793 | 0.000056 | 0.000045 | 0.001801 | 0.000028 | 0.12495861 |
| U1-11 | 0.000125 | 0.000137 | 0.000006 | 0.000005 | 0.000105 | 0.000008 | 0.023925 | 0.000006 | 0.000006 | 0.000006 | 0.005699 | 0.000006 | 0.03003433 |
| U1-12 | 0.00003 | 0.000142 | 0.000006 | 0.000005 | 0.000125 | 0.000008 | 0.024814 | 0.000006 | 0.000007 | 0.000006 | 0.005911 | 0.000007 | 0.03106703 |
| U1-13 | 0.000053 | 0.000255 | 0.000011 | 0.000009 | 0.00011 | 0.000015 | 0.0445 | 0.00001 | 0.000012 | 0.000011 | 0.0106 | 0.000012 | 0.05559891 |
| U1-14 | 0.000027 | 0.000129 | 0.000006 | 0.000005 | 0.000113 | 0.000008 | 0.022475 | 0.000005 | 0.000131 | 0.000006 | 0.005354 | 0.000006 | 0.02826285 |
| U1-15 | 0.000039 | 0.000186 | 0.000008 | 0.000007 | 0.001104 | 0.000011 | 0.032403 | 0.000008 | 0.000009 | 0.000008 | 0.007718 | 0.000009 | 0.04150842 |
| M2-1 | 0.00007 | 0.000034 | 0.000079 | 0.000001 | 0.000223 | 0.000002 | 0.00596 | 0.000006 | 0.000033 | 0.000024 | 0.00142 | 0.000002 | 0.00785461 |
| M2-2 | 0.000106 | 0.000049 | 0.000069 | 0.000002 | 0.000226 | 0.000003 | 0.008525 | 0.000043 | 0.000024 | 0.000024 | 0.002031 | 0.000023 | 0.01112439 |
| M2-3 | 0.000059 | 0.000044 | 0.000161 | 0.000002 | 0.000477 | 0.000003 | 0.126248 | 0.000031 | 0.000035 | 0.000074 | 0.001842 | 0.000064 | 0.1290397 |
| M2-4 | 0.000328 | 0.000054 | 0.001064 | 0.000002 | 0.002748 | 0.000003 | 0.457538 | 0.000045 | 0.000225 | 0.000453 | 0.002227 | 0.000447 | 0.46513177 |
| M2-5 | 0.000068 | 0.000033 | 0.000131 | 0.000001 | 0.000355 | 0.000002 | 0.12005 | 0.000015 | 0.000078 | 0.000019 | 0.001383 | 0.000055 | 0.12219117 |
| M2-6 | 0.000008 | 0.000039 | 0.000228 | 0.000285 | 0.001018 | 0.000002 | 0.00677 | 0.000227 | 0.000103 | 0.000241 | 0.001613 | 0.000056 | 0.01058939 |
| M2-7 | 0.000032 | 0.000154 | 0.001305 | 0.000006 | 0.003094 | 0.000009 | 0.026915 | 0.000711 | 0.000112 | 0.000409 | 0.006411 | 0.000311 | 0.0394697 |
| M2-8 | 0.000009 | 0.000043 | 0.000473 | 0.000002 | 0.001507 | 0.000003 | 0.007559 | 0.000002 | 0.000002 | 0.000079 | 0.001801 | 0.000002 | 0.01148135 |
| M2-9 | 0.000205 | 0.000044 | 0.001558 | 0.000002 | 0.00321 | 0.000003 | 0.007744 | 0.000354 | 0.000193 | 0.000558 | 0.001845 | 0.000146 | 0.01586102 |
| M2-10 | 0.000127 | 0.00005 | 0.000292 | 0.000002 | 0.000781 | 0.000003 | 0.008771 | 0.000246 | 0.000032 | 0.000132 | 0.002089 | 0.000049 | 0.01257356 |
| M2-11 | 0.000162 | 0.000192 | 0.000009 | 0.000007 | 0.000171 | 0.000011 | 0.033543 | 0.000008 | 0.000009 | 0.000009 | 0.00799 | 0.000009 | 0.04211855 |
| M2-12 | 0.000013 | 0.000062 | 0.000017 | 0.000002 | 0.000142 | 0.000004 | 0.010871 | 0.000003 | 0.000003 | 0.000003 | 0.00259 | 0.000003 | 0.0137127 |
| M2-13 | 0.000017 | 0.000081 | 0.000106 | 0.000003 | 0.000344 | 0.000005 | 0.014082 | 0.000003 | 0.000004 | 0.000004 | 0.003354 | 0.000004 | 0.01800629 |
| M2-14 | 0.00001 | 0.000048 | 0.000088 | 0.000002 | 0.001165 | 0.000003 | 0.008333 | 0.000002 | 0.000002 | 0.000002 | 0.001985 | 0.000002 | 0.01164237 |
| M2-15 | 0.00001 | 0.00005 | 0.000046 | 0.00012 | 0.000382 | 0.000003 | 0.008748 | 0.000002 | 0.000002 | 0.000002 | 0.002084 | 0.000002 | 0.01145326 |
| C2-1 | 0.000007 | 0.000033 | 0.000167 | 0.000001 | 0.000326 | 0.000002 | 0.005754 | 0.000078 | 0.000034 | 0.000001 | 0.001371 | 0.000002 | 0.00777562 |
| C2-2 | 0.000026 | 0.00003 | 0.000037 | 0.000001 | 0.000103 | 0.000002 | 0.005215 | 0.000005 | 0.000011 | 0.000014 | 0.001242 | 0.000001 | 0.00668662 |
| C2-3 | 0.000048 | 0.000035 | 0.000112 | 0.000001 | 0.000333 | 0.000002 | 0.065277 | 0.000076 | 0.000028 | 0.000038 | 0.001472 | 0.000019 | 0.06744249 |
| C2-4 | 0.000023 | 0.000109 | 0.000005 | 0.000004 | 0.000252 | 0.000006 | 0.019017 | 0.000004 | 0.000005 | 0.000072 | 0.00453 | 0.000005 | 0.02403242 |
| C2-5 | 0.000007 | 0.000032 | 0.000038 | 0.000001 | 0.000103 | 0.000002 | 0.005517 | 0.000013 | 0.000001 | 0.000013 | 0.001314 | 0.000001 | 0.00704109 |
| C2-6 | 0.000008 | 0.000039 | 0.000002 | 0.000001 | 0.000399 | 0.000002 | 0.006742 | 0.000096 | 0.000002 | 0.000026 | 0.001606 | 0.000002 | 0.00892521 |
| C2-7 | 0.000006 | 0.00003 | 0.000069 | 0.000001 | 0.000266 | 0.000002 | 0.005215 | 0.000089 | 0.000074 | 0.000001 | 0.001242 | 0.000001 | 0.00699679 |
| C2-8 | 0.000124 | 0.000041 | 0.000188 | 0.000001 | 0.000565 | 0.000166 | 0.007109 | 0.000273 | 0.000049 | 0.000067 | 0.001693 | 0.000041 | 0.0103175 |
| C2-9 | 0.000009 | 0.000046 | 0.000598 | 0.000002 | 0.001199 | 0.000003 | 0.007946 | 0.000002 | 0.000002 | 0.000256 | 0.001893 | 0.000002 | 0.01195854 |
| C2-10 | 0.000092 | 0.000039 | 0.000125 | 0.000001 | 0.001368 | 0.000341 | 0.006818 | 0.000017 | 0.000002 | 0.00003 | 0.001624 | 0.000002 | 0.01045938 |
| C2-11 | 0.000095 | 0.000039 | 0.000002 | 0.000001 | 0.000112 | 0.000002 | 0.00677 | 0.000002 | 0.000145 | 0.000002 | 0.001613 | 0.000002 | 0.00878385 |
| C2-12 | 0.000009 | 0.000044 | 0.000002 | 0.000002 | 0.000112 | 0.000003 | 0.007603 | 0.000002 | 0.000002 | 0.000002 | 0.001811 | 0.000002 | 0.00959165 |
| C2-13 | 0.000101 | 0.000056 | 0.000002 | 0.000002 | 0.000075 | 0.000003 | 0.009816 | 0.000002 | 0.000003 | 0.000003 | 0.002338 | 0.000003 | 0.01240469 |
| C2-14 | 0.000086 | 0.000045 | 0.000002 | 0.000002 | 0.000097 | 0.000003 | 0.007909 | 0.000002 | 0.000002 | 0.000002 | 0.001884 | 0.000002 | 0.01003559 |
| C2-15 | 0.000221 | 0.000047 | 0.000002 | 0.000002 | 0.000182 | 0.000003 | 0.008251 | 0.000002 | 0.000002 | 0.000002 | 0.001965 | 0.000002 | 0.01068176 |
| U2-1 | 0.000011 | 0.000052 | 0.000037 | 0.000002 | 0.000132 | 0.000003 | 0.009082 | 0.000074 | 0.000002 | 0.000016 | 0.002163 | 0.000002 | 0.01157661 |
| U2-2 | 0.000142 | 0.000043 | 0.000046 | 0.000002 | 0.000122 | 0.000003 | 0.007517 | 0.000047 | 0.000002 | 0.000002 | 0.001791 | 0.000002 | 0.00971723 |
| U2-3 | 0.000009 | 0.000045 | 0.000091 | 0.000002 | 0.000418 | 0.000003 | 0.007928 | 0.000002 | 0.000002 | 0.000029 | 0.001888 | 0.000038 | 0.01045549 |
| U2-4 | 0.000009 | 0.000045 | 0.000056 | 0.000002 | 0.00017 | 0.000003 | 0.007918 | 0.000092 | 0.000002 | 0.000019 | 0.001886 | 0.000015 | 0.01021705 |
| U2-5 | 0.000007 | 0.000032 | 0.000714 | 0.001461 | 0.001787 | 0.000002 | 0.005609 | 0.000101 | 0.000001 | 0.000026 | 0.001336 | 0.000001 | 0.01107793 |
| U2-6 | 0.000009 | 0.000042 | 0.00009 | 0.000001 | 0.000275 | 0.000002 | 0.060284 | 0.000045 | 0.000002 | 0.000002 | 0.001736 | 0.000002 | 0.06249 |
| U2-7 | 0.000011 | 0.001031 | 0.000126 | 0.000002 | 0.000253 | 0.000003 | 0.009069 | 0.000002 | 0.000002 | 0.000022 | 0.00216 | 0.000002 | 0.01268291 |
| U2-8 | 0.000094 | 0.000251 | 0.000075 | 0.000002 | 0.000155 | 0.000003 | 0.007483 | 0.000002 | 0.000002 | 0.000024 | 0.001783 | 0.000002 | 0.00987329 |
| U2-9 | 0.000081 | 0.000053 | 0.00002 | 0.000002 | 0.000122 | 0.000003 | 0.009245 | 0.000046 | 0.000002 | 0.000002 | 0.002202 | 0.000002 | 0.01178126 |
| U2-10 | 0.000009 | 0.000041 | 0.000032 | 0.000001 | 0.000103 | 0.000002 | 0.007232 | 0.000036 | 0.000002 | 0.000014 | 0.001723 | 0.000002 | 0.00919738 |
| U2-11 | 0.000011 | 0.000055 | 0.000043 | 0.000002 | 0.000066 | 0.000003 | 0.009577 | 0.000002 | 0.000003 | 0.000002 | 0.002281 | 0.000003 | 0.0120481 |
| U2-12 | 0.000014 | 0.000066 | 0.000039 | 0.000002 | 0.000102 | 0.000004 | 0.011528 | 0.000003 | 0.000003 | 0.000003 | 0.002746 | 0.000003 | 0.0145136 |
| U2-13 | 0.000022 | 0.000104 | 0.000005 | 0.000004 | 0.000106 | 0.000006 | 0.249288 | 0.000004 | 0.000005 | 0.000005 | 0.004321 | 0.000005 | 0.25387294 |
| U2-14 | 0.000015 | 0.000073 | 0.000003 | 0.000003 | 0.000185 | 0.000004 | 0.012763 | 0.000051 | 0.000003 | 0.000003 | 0.00304 | 0.000003 | 0.01614727 |
| U2-15 | 0.000011 | 0.000055 | 0.000002 | 0.000002 | 0.000105 | 0.000003 | 0.009618 | 0.000002 | 0.000003 | 0.000002 | 0.002291 | 0.000003 | 0.01209816 |

**TABLE S4: Characteristics of analyzed fish samples.**

| **ID** | **Source** | **Species** | **Length (cm)** | **Weight (g)** | **Fat (%)** |
| --- | --- | --- | --- | --- | --- |
| M1-1 | Magdalena River | *P magdaleniatum* | 63.2 | 1650 | 5.00 |
| M1-2 | Magdalena River | *P magdaleniatum* | 62.7 | 1470 | 4.07 |
| M1-3 | Magdalena River | *P magdaleniatum* | 68.5 | 1710 | 1.11 |
| M1-4 | Magdalena River | *P magdaleniatum* | 65.6 | 1740 | 0.524 |
| M1-5 | Magdalena River | *P magdaleniatum* | 63.6 | 1430 | 7.94 |
| M1-6 | Magdalena River | *S cuspicaudus* | 52.4 | 731 | 1.75 |
| M1-7 | Magdalena River | *S cuspicaudus* | 52.3 | 681 | 0.682 |
| M1-8 | Magdalena River | *S cuspicaudus* | 58.0 | 1140 | 3.11 |
| M1-9 | Magdalena River | *S cuspicaudus* | 57.5 | 1020 | 8.66 |
| M1-10 | Magdalena River | *S cuspicaudus* | 55.0 | 1120 | 18.5 |
| M1-11 | Magdalena River | *P magdalenae* | 39.1 | 507 | 0.232 |
| M1-12 | Magdalena River | *P magdalenae* | 28.4 | 197 | 1.06 |
| M1-13 | Magdalena River | *P magdalenae* | 28.1 | 214 | 1.57 |
| M1-14 | Magdalena River | *P magdalenae* | 26.6 | 197 | 2.85 |
| M1-15 | Magdalena River | *P magdalenae* | 35.7 | 405 | 0.302 |
| C1-1 | Cauca River | *P magdaleniatum* | 122 | 15200 | 4.73 |
| C1-2 | Cauca River | *P magdaleniatum* | 93.6 | 4310 | 8.89 |
| C1-3 | Cauca River | *P magdaleniatum* | 76.3 | 2950 | 4.02 |
| C1-4 | Cauca River | *P magdaleniatum* | 74.0 | 2490 | 4.12 |
| C1-5 | Cauca River | *P magdaleniatum* | 93.2 | 5670 | 2.70 |
| C1-6 | Cauca River | *S cuspicaudus* | 70.1 | 2270 | 5.42 |
| C1-7 | Cauca River | *S cuspicaudus* | 59.1 | 1220 | 6.17 |
| C1-8 | Cauca River | *S cuspicaudus* | 56.0 | 680 | 3.55 |
| C1-9 | Cauca River | *S cuspicaudus* | 54.0 | 680 | 4.25 |
| C1-10 | Cauca River | *S cuspicaudus* | 61.2 | 1590 | 2.86 |
| C1-11 | Cauca River | *P magdalenae* | 30.1 | 363 | 2.76 |
| C1-12 | Cauca River | *P magdalenae* | 32.3 | 454 | 7.35 |
| C1-13 | Cauca River | *P magdalenae* | 29.6 | 363 | 4.83 |
| C1-14 | Cauca River | *P magdalenae* | 32.6 | 454 | 6.42 |
| C1-15 | Cauca River | *P magdalenae* | 30.6 | 408 | 5.72 |
| U1-1 | Gulf of Urabá | *B marinus* | 69.1 | 2270 | 0.753 |
| U1-2 | Gulf of Urabá | *B marinus* | 65.4 | 2380 | 0.731 |
| U1-3 | Gulf of Urabá | *B marinus* | 73.0 | 2840 | 0.908 |
| U1-4 | Gulf of Urabá | *B marinus* | 66.5 | 2490 | 2.28 |
| U1-5 | Gulf of Urabá | *B marinus* | 72.1 | 3400 | 0.814 |
| U1-6 | Gulf of Urabá | *C undecimalis* | 48.0 | 1000 | 0.511 |
| U1-7 | Gulf of Urabá | *C undecimalis* | 49.1 | 1050 | 0.317 |
| U1-8 | Gulf of Urabá | *C undecimalis* | 51.3 | 1100 | 0.97 |
| U1-9 | Gulf of Urabá | *C undecimalis* | 51.5 | 1200 | 2.24 |
| U1-10 | Gulf of Urabá | *C undecimalis* | 55.5 | 1400 | 1.69 |
| U1-11 | Gulf of Urabá | *T lepturus* | 71.0 | 250 | 1.72 |
| U1-12 | Gulf of Urabá | *T lepturus* | 76.0 | 300 | 2.68 |
| U1-13 | Gulf of Urabá | *T lepturus* | 80.0 | 400 | 1.05 |
| U1-14 | Gulf of Urabá | *T lepturus* | 79.0 | 380 | 0.887 |
| U1-15 | Gulf of Urabá | *T lepturus* | 73.0 | 300 | 1.45 |
| M2-1 | Magdalena River | *P magdaleniatum* | 67.7 | 3180 | 2.63 |
| M2-2 | Magdalena River | *P magdaleniatum* | 72.5 | 2380 | 2.64 |
| M2-3 | Magdalena River | *P magdaleniatum* | 71.8 | 2270 | 3.29 |
| M2-4 | Magdalena River | *P magdaleniatum* | 72.5 | 2610 | 6.72 |
| M2-5 | Magdalena River | *P magdaleniatum* | 77.0 | 2720 | 2.09 |
| M2-6 | Magdalena River | *S cuspicaudus* | 60.3 | 1360 | 10.8 |
| M2-7 | Magdalena River | *S cuspicaudus* | 63.2 | 1700 | 10.2 |
| M2-8 | Magdalena River | *S cuspicaudus* | 56.0 | 1250 | 6.04 |
| M2-9 | Magdalena River | *S cuspicaudus* | 61.0 | 1360 | 11.8 |
| M2-10 | Magdalena River | *S cuspicaudus* | 62.2 | 1590 | 5.57 |
| M2-11 | Magdalena River | *P magdalenae* | 30.3 | 454 | 1.46 |
| M2-12 | Magdalena River | *P magdalenae* | 32.8 | 454 | 0.912 |
| M2-13 | Magdalena River | *P magdalenae* | 31.0 | 454 | 2.53 |
| M2-14 | Magdalena River | *P magdalenae* | 34.3 | 567 | 4.64 |
| M2-15 | Magdalena River | *P magdalenae* | 35.0 | 567 | 2.10 |
| C2-1 | Cauca River | *P magdaleniatum* | 89.0 | 4990 | 3.39 |
| C2-2 | Cauca River | *P magdaleniatum* | 89.0 | 4990 | 2.42 |
| C2-3 | Cauca River | *P magdaleniatum* | 105 | 7260 | 1.32 |
| C2-4 | Cauca River | *P magdaleniatum* | 116 | 8390 | 0.650 |
| C2-5 | Cauca River | *P magdaleniatum* | 76.0 | 2720 | 2.31 |
| C2-6 | Cauca River | *S cuspicaudus* | 62.0 | 1360 | 11.0 |
| C2-7 | Cauca River | *S cuspicaudus* | 56.0 | 1130 | 3.74 |
| C2-8 | Cauca River | *S cuspicaudus* | 58.0 | 907 | 2.73 |
| C2-9 | Cauca River | *S cuspicaudus* | 62.0 | 1360 | 8.99 |
| C2-10 | Cauca River | *S cuspicaudus* | 63.0 | 1590 | 7.95 |
| C2-11 | Cauca River | *P magdalenae* | 34.6 | 454 | 2.87 |
| C2-12 | Cauca River | *P magdalenae* | 32.3 | 408 | 2.24 |
| C2-13 | Cauca River | *P magdalenae* | 31.5 | 499 | 3.09 |
| C2-14 | Cauca River | *P magdalenae* | 31.5 | 340 | 2.44 |
| C2-15 | Cauca River | *P magdalenae* | 31.5 | 340 | 2.10 |
| U2-1 | Gulf of Urabá | *B marinus* | 58.4 | 1600 | 0.694 |
| U2-2 | Gulf of Urabá | *B marinus* | 57.2 | 1450 | 0.450 |
| U2-3 | Gulf of Urabá | *B marinus* | 59.8 | 1800 | 1.35 |
| U2-4 | Gulf of Urabá | *B marinus* | 53.8 | 1200 | 0.391 |
| U2-5 | Gulf of Urabá | *B marinus* | 62.2 | 1900 | 2.47 |
| U2-6 | Gulf of Urabá | *C undecimalis* | 69.3 | 3250 | 2.60 |
| U2-7 | Gulf of Urabá | *C undecimalis* | 68.4 | 3500 | 2.23 |
| U2-8 | Gulf of Urabá | *C undecimalis* | 62.2 | 2400 | 3.09 |
| U2-9 | Gulf of Urabá | *C undecimalis* | 64.0 | 2200 | 1.51 |
| U2-10 | Gulf of Urabá | *C undecimalis* | 56.3 | 1600 | 1.71 |
| U2-11 | Gulf of Urabá | *T lepturus* | 81.7 | 400 | 0.761 |
| U2-12 | Gulf of Urabá | *T lepturus* | 73.3 | 300 | 1.81 |
| U2-13 | Gulf of Urabá | *T lepturus* | 71.5 | 250 | 0.408 |
| U2-14 | Gulf of Urabá | *T lepturus* | 72.4 | 300 | 0.229 |
| U2-15 | Gulf of Urabá | *T lepturus* | 71.3 | 300 | 1.87 |

**TABLE S5 (a): Individual results of PCDD/PCDF in the fish samples (n=90, pg/g w.w).**

| **ID** | **2,3,7,8-TCDF** | **2,3,7,8-TCDD** | **1,2,3,7,8-PeCDF** | **2,3,4,7,8-PeCDF** | **1,2,3,7,8-PeCDD** | **1,2,3,4,7,8-HxCDF** | **1,2,3,6,7,8-HxCDF** | **1,2,3,4,7,8-HxCDD** | **2,3,4,6,7,8-HxCDF** | **1,2,3,6,7,8-HxCDD** | **1,2,3,7,8,9-HxCDD** | **1,2,3,7,8,9-HxCDF** | **1,2,3,4,6,7,8-HpCDF** | **1,2,3,4,6,7,8-HpCDD** | **1,2,3,4,7,8,9-HpCDF** | **OCDD** | **OCDF** |
| --- | --- | --- | --- | --- | --- | --- | --- | --- | --- | --- | --- | --- | --- | --- | --- | --- | --- |
| M1-1 | <0.082 | <0.0961 | <0.119 | <0.0801 | <0.374 | <0.074 | <0.101 | <0.374 | <0.0805 | <0.374 | <0.374 | <0.0644 | <0.293 | <0.0775 | <0.0736 | <0.84 | <0.131 |
| M1-2 | <0.0963 | <0.113 | <0.14 | <0.094 | <0.439 | <0.0869 | <0.119 | <0.439 | <0.0945 | <0.439 | <0.439 | <0.0756 | <0.344 | <0.091 | <0.0863 | <0.985 | <0.154 |
| M1-3 | <0.111 | <0.13 | <0.161 | <0.109 | <0.507 | <0.1 | <0.137 | <0.507 | <0.109 | <0.507 | <0.507 | <0.0874 | <0.398 | <0.105 | <0.0997 | <1.14 | <0.178 |
| M1-4 | <0.0993 | <0.116 | <0.144 | <0.097 | <0.452 | <0.0896 | <0.122 | <0.452 | <0.0974 | <0.452 | <0.452 | <0.078 | <0.355 | <0.0938 | <0.0891 | <1.02 | <0.159 |
| M1-5 | <0.0982 | <0.115 | <0.142 | <0.0959 | <0.447 | <0.0886 | <0.121 | <0.447 | <0.0963 | <0.447 | <0.447 | <0.0771 | <0.351 | <0.0928 | <0.088 | <1 | <0.157 |
| M1-6 | <0.112 | <0.131 | <0.162 | <0.109 | <0.51 | <0.101 | <0.138 | <0.51 | <0.11 | <0.51 | <0.51 | <0.0879 | <0.401 | <0.106 | <0.1 | <1.15 | <0.179 |
| M1-7 | <0.108 | <0.126 | <0.156 | <0.105 | <0.491 | <0.0972 | <0.133 | <0.491 | <0.106 | <0.491 | <0.491 | <0.0846 | <0.385 | <0.102 | <0.0966 | <1.1 | <0.172 |
| M1-8 | <0.0952 | <0.111 | <0.138 | <0.093 | <0.434 | <0.0859 | <0.117 | <0.434 | <0.0934 | <0.434 | <0.434 | <0.0748 | <0.341 | <0.09 | <0.171 | <0.974 | <0.152 |
| M1-9 | <0.0905 | <0.106 | <0.131 | <0.0883 | <0.412 | <0.0817 | <0.111 | <0.412 | <0.0888 | <0.412 | <0.412 | <0.071 | <0.324 | <0.0855 | <0.0811 | <0.926 | <0.145 |
| M1-10 | <0.103 | <0.121 | <0.15 | <0.101 | <0.47 | <0.0932 | <0.127 | <0.47 | <0.101 | <0.47 | <0.47 | <0.0811 | <0.369 | <0.0976 | <0.0926 | <1.06 | <0.165 |
| M1-11 | <0.104 | <0.122 | <0.151 | <0.102 | <0.474 | <0.094 | <0.128 | <0.474 | <0.102 | <0.474 | <0.474 | <0.0818 | <0.373 | <0.0984 | <0.0934 | <1.07 | <0.167 |
| M1-12 | <0.431 | <0.505 | <0.624 | <0.421 | <1.96 | <0.389 | <0.53 | <1.96 | <0.423 | <1.96 | <1.96 | <0.338 | <1.54 | <0.407 | <0.386 | <4.41 | <0.69 |
| M1-13 | <0.336 | <0.393 | <0.487 | <0.328 | <1.53 | <0.303 | <0.413 | <1.53 | <0.33 | <1.53 | <1.53 | <0.264 | <1.20 | <0.317 | <0.301 | <3.44 | <0.538 |
| M1-14 | <0.485 | <0.568 | <0.702 | <0.473 | <2.21 | <0.438 | <0.597 | <2.21 | <0.476 | <2.21 | <2.21 | <0.381 | <1.73 | <0.458 | <0.435 | <4.96 | <0.776 |
| M1-15 | <0.22 | <0.258 | <0.319 | <0.215 | <1.00 | <0.199 | <0.271 | <1.00 | <0.216 | <1.00 | <1.00 | <0.173 | <0.789 | <0.208 | <0.198 | <2.26 | <0.353 |
| C1-1 | <0.0797 | <0.0934 | <0.116 | <0.0779 | <0.363 | <0.072 | <0.0981 | <0.363 | <0.0782 | <0.363 | <0.363 | <0.0626 | <0.285 | <0.0753 | <0.143 | <0.816 | <0.128 |
| C1-2 | <0.0958 | <0.112 | <0.139 | <0.0936 | <0.437 | <0.0865 | <0.118 | <0.437 | <0.094 | <0.437 | <0.437 | <0.0753 | <0.343 | <0.0906 | <0.086 | <0.981 | <0.153 |
| C1-3 | <0.0958 | <0.112 | <0.139 | <0.0936 | <0.437 | <0.0865 | <0.118 | <0.437 | <0.094 | <0.437 | <0.437 | <0.0753 | <0.343 | <0.0906 | <0.172 | <0.981 | <0.153 |
| C1-4 | <0.107 | <0.125 | <0.155 | <0.104 | <0.486 | <0.0964 | <0.131 | <0.486 | <0.105 | <0.486 | <0.486 | <0.0839 | <0.382 | <0.101 | <0.0957 | <1.09 | <0.171 |
| C1-5 | <0.112 | <0.131 | <0.162 | <0.109 | <0.51 | <0.101 | <0.138 | <0.51 | <0.11 | <0.51 | <0.51 | <0.0879 | <0.401 | <0.106 | <0.1 | <1.15 | <0.179 |
| C1-6 | <0.102 | <0.119 | <0.147 | <0.0993 | <0.463 | <0.0918 | <0.125 | <0.463 | <0.0998 | <0.463 | <0.463 | <0.0799 | <0.364 | <0.0961 | <0.182 | <1.04 | <0.163 |
| C1-7 | <0.0729 | <0.0854 | <0.106 | <0.0712 | <0.332 | <0.0658 | <0.0897 | <0.332 | <0.0715 | <0.332 | <0.332 | <0.0573 | <0.261 | <0.0689 | <0.0654 | <0.746 | <0.117 |
| C1-8 | <0.101 | <0.118 | <0.146 | <0.0987 | <0.46 | <0.0912 | <0.124 | <0.46 | <0.0992 | <0.46 | <0.46 | <0.0794 | <0.362 | <0.0955 | <0.181 | <1.03 | <0.162 |
| C1-9 | <0.0909 | <0.107 | <0.132 | <0.0888 | <0.414 | <0.0821 | <0.112 | <0.414 | <0.0892 | <0.414 | <0.414 | <0.0714 | <0.325 | <0.0859 | <0.163 | <0.931 | <0.146 |
| C1-10 | <0.0874 | <0.102 | <0.127 | <0.0853 | <0.398 | <0.0789 | <0.108 | <0.398 | <0.0858 | <0.398 | <0.398 | <0.0686 | <0.313 | <0.0826 | <0.0784 | <0.895 | <0.14 |
| C1-11 | <0.0905 | <0.106 | <0.131 | <0.0883 | <0.412 | <0.0817 | <0.111 | <0.412 | <0.0888 | <0.412 | <0.412 | <0.071 | <0.324 | <0.0855 | <0.0811 | <0.926 | <0.145 |
| C1-12 | <0.212 | <0.248 | <0.307 | <0.207 | <0.964 | <0.191 | <0.261 | <0.964 | <0.208 | <0.964 | <0.964 | <0.166 | <0.757 | <0.2 | <0.19 | <2.17 | <0.339 |
| C1-13 | <0.12 | <0.141 | <0.174 | <0.117 | <0.548 | <0.109 | <0.148 | <0.548 | <0.118 | <0.548 | <0.548 | <0.0945 | <0.43 | <0.114 | <0.108 | <1.23 | <0.193 |
| C1-14 | <0.121 | <0.142 | <0.175 | <0.118 | <0.551 | <0.109 | <0.149 | <0.551 | <0.119 | <0.551 | <0.551 | <0.095 | <0.433 | <0.114 | <0.109 | <1.24 | <0.194 |
| C1-15 | <0.138 | <0.161 | <0.199 | <0.134 | <0.627 | <0.124 | <0.169 | <0.627 | <0.135 | <0.627 | <0.627 | <0.108 | <0.492 | <0.13 | <0.123 | <1.41 | <0.22 |
| U1-1 | <0.0641 | <0.0751 | <0.0929 | <0.0626 | <0.292 | <0.0579 | <0.0789 | <0.292 | <0.0629 | <0.292 | <0.292 | <0.0504 | <0.229 | <0.0606 | <0.0575 | <0.656 | <0.103 |
| U1-2 | <0.0783 | <0.0917 | <0.113 | <0.0764 | <0.357 | <0.0706 | <0.0963 | <0.357 | <0.0768 | <0.357 | <0.357 | <0.0615 | <0.28 | <0.0739 | <0.0702 | <0.801 | <0.125 |
| U1-3 | <0.109 | <0.128 | <0.158 | <0.107 | <0.497 | <0.0985 | <0.134 | <0.497 | <0.107 | <0.497 | <0.497 | <0.0857 | <0.39 | <0.103 | <0.0978 | <1.12 | <0.175 |
| U1-4 | <0.0711 | <0.0833 | <0.103 | <0.0694 | <0.324 | <0.0642 | <0.0875 | <0.324 | <0.0698 | <0.324 | <0.324 | <0.0558 | <0.254 | <0.0672 | <0.128 | <0.728 | <0.114 |
| U1-5 | <0.112 | <0.131 | <0.162 | <0.109 | <0.51 | <0.101 | <0.138 | <0.51 | <0.11 | <0.51 | <0.51 | <0.0879 | <0.401 | <0.106 | <0.1 | <1.15 | <0.179 |
| U1-6 | <0.115 | <0.134 | <0.166 | <0.112 | <0.522 | <0.103 | <0.141 | <0.522 | <0.113 | <0.522 | <0.522 | <0.0901 | <0.41 | <0.108 | <0.206 | <1.17 | <0.184 |
| U1-7 | <0.0932 | <0.109 | <0.135 | <0.091 | <0.425 | <0.0842 | <0.115 | <0.425 | <0.0915 | <0.425 | <0.425 | <0.0732 | <0.334 | <0.0881 | <0.167 | <0.954 | <0.149 |
| U1-8 | <0.11 | <0.129 | <0.16 | <0.108 | <0.503 | <0.0996 | <0.136 | <0.503 | <0.108 | <0.503 | <0.503 | <0.0867 | <0.395 | <0.104 | <0.198 | <1.13 | <0.177 |
| U1-9 | <0.0735 | <0.0861 | <0.107 | <0.0718 | <0.335 | <0.0664 | <0.0905 | <0.335 | <0.0722 | <0.335 | <0.335 | <0.0578 | <0.263 | <0.0695 | <0.132 | <0.753 | <0.118 |
| U1-10 | <0.0966 | <0.113 | <0.14 | <0.0943 | <0.44 | <0.0872 | <0.119 | <0.44 | <0.0948 | <0.44 | <0.44 | <0.0759 | <0.346 | <0.0913 | <0.173 | <0.989 | <0.155 |
| U1-11 | <0.306 | <0.358 | <0.443 | <0.299 | <1.39 | <0.276 | <0.376 | <1.39 | <0.3 | <1.39 | <1.39 | <0.24 | <1.09 | <0.289 | <0.274 | <3.13 | <0.49 |
| U1-12 | <0.317 | <0.371 | <0.459 | <0.31 | <1.44 | <0.286 | <0.39 | <1.44 | <0.311 | <1.44 | <1.44 | <0.249 | <1.13 | <0.3 | <0.284 | <3.25 | <0.508 |
| U1-13 | <0.569 | <0.666 | <0.824 | <0.555 | <2.59 | <0.513 | <0.7 | <2.59 | <0.558 | <2.59 | <2.59 | <0.447 | <2.03 | <0.537 | <0.51 | <5.82 | <0.911 |
| U1-14 | <0.287 | <0.336 | <0.416 | <0.28 | <1.31 | <0.259 | <0.354 | <1.31 | <0.282 | <1.31 | <1.31 | <0.226 | <1.03 | <0.271 | <0.515 | <2.94 | <0.46 |
| U1-15 | <0.414 | <0.485 | <0.6 | <0.404 | <1.89 | <0.374 | <0.51 | <1.89 | <0.406 | <1.89 | <1.89 | <0.325 | <1.48 | <0.391 | <0.371 | <4.24 | <0.663 |
| M2-1 | <0.0762 | <0.0892 | <0.11 | <0.0744 | <0.347 | <0.0688 | <0.0938 | <0.347 | <0.0747 | <0.347 | <0.347 | <0.0598 | <0.273 | <0.072 | <0.137 | <0.78 | <0.122 |
| M2-2 | <0.109 | <0.128 | <0.158 | <0.106 | <0.496 | <0.0983 | <0.134 | <0.496 | <0.107 | <0.496 | <0.496 | <0.0856 | <0.39 | <0.103 | <0.195 | <1.12 | <0.174 |
| M2-3 | <0.0988 | <0.116 | <0.143 | <0.0965 | <0.45 | <0.0892 | <0.122 | <0.45 | <0.097 | <0.45 | <0.45 | <0.0776 | <0.354 | <0.0934 | <0.177 | <1.01 | <0.158 |
| M2-4 | <0.119 | <0.14 | <0.173 | <0.117 | <0.544 | <0.108 | <0.147 | <0.544 | <0.117 | <0.544 | <0.544 | <0.0938 | <0.427 | <0.113 | <0.214 | <1.22 | <0.191 |
| M2-5 | <0.0742 | <0.0869 | <0.107 | <0.0724 | <0.338 | <0.067 | <0.0913 | <0.338 | <0.0728 | <0.338 | <0.338 | <0.0583 | <0.265 | <0.0701 | <0.133 | <0.759 | <0.119 |
| M2-6 | <0.0865 | <0.101 | <0.125 | <0.0845 | <0.394 | <0.0781 | <0.106 | <0.394 | <0.0849 | <0.394 | <0.394 | <0.068 | <0.31 | <0.0817 | <0.155 | <0.885 | <0.139 |
| M2-7 | <0.344 | <0.403 | <0.498 | <0.336 | <1.57 | <0.31 | <0.423 | <1.57 | <0.338 | <1.57 | <1.57 | <0.27 | <1.23 | <0.325 | <0.617 | <3.52 | <0.551 |
| M2-8 | <0.0966 | <0.113 | <0.14 | <0.0943 | <0.44 | <0.0872 | <0.119 | <0.44 | <0.0948 | <0.44 | <0.44 | <0.0759 | <0.346 | <0.0913 | <0.0866 | <0.989 | <0.155 |
| M2-9 | <0.099 | <0.116 | <0.143 | <0.0966 | <0.451 | <0.0893 | <0.122 | <0.451 | <0.0971 | <0.451 | <0.451 | <0.0777 | <0.354 | <0.0935 | <0.177 | <1.01 | <0.158 |
| M2-10 | <0.112 | <0.131 | <0.162 | <0.109 | <0.511 | <0.101 | <0.138 | <0.511 | <0.11 | <0.511 | <0.511 | <0.088 | <0.401 | <0.106 | <0.201 | <1.15 | <0.18 |
| M2-11 | <0.429 | <0.502 | <0.621 | <0.419 | <1.95 | <0.387 | <0.528 | <1.95 | <0.421 | <1.95 | <1.95 | <0.337 | <1.53 | <0.405 | <0.384 | <4.39 | <0.686 |
| M2-12 | <0.139 | <0.163 | <0.201 | <0.136 | <0.633 | <0.125 | <0.171 | <0.633 | <0.136 | <0.633 | <0.633 | <0.109 | <0.497 | <0.131 | <0.125 | <1.42 | <0.222 |
| M2-13 | <0.18 | <0.211 | <0.261 | <0.176 | <0.82 | <0.162 | <0.222 | <0.82 | <0.177 | <0.82 | <0.82 | <0.141 | <0.644 | <0.17 | <0.161 | <1.84 | <0.288 |
| M2-14 | <0.106 | <0.125 | <0.154 | <0.104 | <0.485 | <0.0961 | <0.131 | <0.485 | <0.104 | <0.485 | <0.485 | <0.0836 | <0.381 | <0.101 | <0.0955 | <1.09 | <0.171 |
| M2-15 | <0.112 | <0.131 | <0.162 | <0.109 | <0.509 | <0.101 | <0.138 | <0.509 | <0.11 | <0.509 | <0.509 | <0.0878 | <0.4 | <0.106 | <0.1 | <1.14 | <0.179 |
| C2-1 | <0.0735 | <0.0861 | <0.107 | <0.0718 | <0.335 | <0.0664 | <0.0905 | <0.335 | <0.0722 | <0.335 | <0.335 | <0.0578 | <0.263 | <0.0695 | <0.132 | <0.753 | <0.118 |
| C2-2 | <0.0666 | <0.078 | <0.0966 | <0.0651 | <0.304 | <0.0602 | <0.082 | <0.304 | <0.0654 | <0.304 | <0.304 | <0.0523 | <0.238 | <0.063 | <0.12 | <0.682 | <0.107 |
| C2-3 | <0.079 | <0.0925 | <0.114 | <0.0771 | <0.36 | <0.0713 | <0.0972 | <0.36 | <0.0775 | <0.36 | <0.36 | <0.062 | <0.283 | <0.0746 | <0.142 | <0.808 | <0.126 |
| C2-4 | <0.243 | <0.285 | <0.352 | <0.237 | <1.11 | <0.219 | <0.299 | <1.11 | <0.238 | <1.11 | <1.11 | <0.191 | <0.87 | <0.23 | <0.218 | <2.49 | <0.389 |
| C2-5 | <0.0705 | <0.0826 | <0.102 | <0.0688 | <0.321 | <0.0636 | <0.0868 | <0.321 | <0.0692 | <0.321 | <0.321 | <0.0554 | <0.252 | <0.0666 | <0.0632 | <0.722 | <0.113 |
| C2-6 | <0.0862 | <0.101 | <0.125 | <0.0841 | <0.393 | <0.0778 | <0.106 | <0.393 | <0.0845 | <0.393 | <0.393 | <0.0677 | <0.308 | <0.0814 | <0.0773 | <0.882 | <0.138 |
| C2-7 | <0.0666 | <0.078 | <0.0966 | <0.0651 | <0.304 | <0.0602 | <0.082 | <0.304 | <0.0654 | <0.304 | <0.304 | <0.0523 | <0.238 | <0.063 | <0.12 | <0.682 | <0.107 |
| C2-8 | <0.0908 | <0.106 | <0.132 | <0.0887 | <0.414 | <0.082 | <0.112 | <0.414 | <0.0891 | <0.414 | <0.414 | <0.0714 | <0.325 | <0.0858 | <0.163 | <0.93 | <0.145 |
| C2-9 | <0.102 | <0.119 | <0.147 | <0.0992 | <0.463 | <0.0917 | <0.125 | <0.463 | <0.0996 | <0.463 | <0.463 | <0.0798 | <0.363 | <0.096 | <0.0911 | <1.04 | <0.163 |
| C2-10 | <0.0871 | <0.102 | <0.126 | <0.0851 | <0.397 | <0.0787 | <0.107 | <0.397 | <0.0855 | <0.397 | <0.397 | <0.0684 | <0.312 | <0.0823 | <0.0781 | <0.892 | <0.14 |
| C2-11 | <0.0865 | <0.101 | <0.125 | <0.0845 | <0.394 | <0.0781 | <0.106 | <0.394 | <0.0849 | <0.394 | <0.394 | <0.068 | <0.31 | <0.0817 | <0.155 | <0.885 | <0.139 |
| C2-12 | <0.0972 | <0.114 | <0.141 | <0.0949 | <0.443 | <0.0877 | <0.12 | <0.443 | <0.0953 | <0.443 | <0.443 | <0.0763 | <0.348 | <0.0918 | <0.0871 | <0.994 | <0.156 |
| C2-13 | <0.125 | <0.147 | <0.182 | <0.123 | <0.571 | <0.113 | <0.154 | <0.571 | <0.123 | <0.571 | <0.571 | <0.0985 | <0.449 | <0.119 | <0.113 | <1.28 | <0.201 |
| C2-14 | <0.101 | <0.118 | <0.146 | <0.0987 | <0.46 | <0.0912 | <0.124 | <0.46 | <0.0992 | <0.46 | <0.46 | <0.0794 | <0.362 | <0.0955 | <0.0906 | <1.03 | <0.162 |
| C2-15 | <0.105 | <0.123 | <0.153 | <0.103 | <0.48 | <0.0952 | <0.13 | <0.48 | <0.103 | <0.48 | <0.48 | <0.0828 | <0.377 | <0.0996 | <0.0946 | <1.08 | <0.169 |
| U2-1 | <0.116 | <0.136 | <0.168 | <0.113 | <0.529 | <0.105 | <0.143 | <0.529 | <0.114 | <0.529 | <0.529 | <0.0912 | <0.415 | <0.11 | <0.104 | <1.19 | <0.186 |
| U2-2 | <0.0961 | <0.113 | <0.139 | <0.0938 | <0.438 | <0.0867 | <0.118 | <0.438 | <0.0943 | <0.438 | <0.438 | <0.0755 | <0.344 | <0.0908 | <0.0861 | <0.983 | <0.154 |
| U2-3 | <0.101 | <0.119 | <0.147 | <0.0989 | <0.462 | <0.0914 | <0.125 | <0.462 | <0.0994 | <0.462 | <0.462 | <0.0796 | <0.362 | <0.0957 | <0.0909 | <1.04 | <0.162 |
| U2-4 | <0.101 | <0.119 | <0.147 | <0.0988 | <0.461 | <0.0913 | <0.125 | <0.461 | <0.0993 | <0.461 | <0.461 | <0.0795 | <0.362 | <0.0956 | <0.0907 | <1.04 | <0.162 |
| U2-5 | <0.0717 | <0.0839 | <0.104 | <0.07 | <0.327 | <0.0647 | <0.0882 | <0.327 | <0.0703 | <0.327 | <0.327 | <0.0563 | <0.256 | <0.0677 | <0.0643 | <0.734 | <0.115 |
| U2-6 | <0.0931 | <0.109 | <0.135 | <0.0909 | <0.424 | <0.0841 | <0.115 | <0.424 | <0.0914 | <0.424 | <0.424 | <0.0731 | <0.333 | <0.088 | <0.0835 | <0.953 | <0.149 |
| U2-7 | <0.116 | <0.136 | <0.168 | <0.113 | <0.528 | <0.105 | <0.143 | <0.528 | <0.114 | <0.528 | <0.528 | <0.091 | <0.415 | <0.11 | <0.104 | <1.19 | <0.186 |
| U2-8 | <0.0956 | <0.112 | <0.139 | <0.0934 | <0.436 | <0.0863 | <0.118 | <0.436 | <0.0938 | <0.436 | <0.436 | <0.0751 | <0.342 | <0.0904 | <0.0858 | <0.979 | <0.153 |
| U2-9 | <0.118 | <0.138 | <0.171 | <0.115 | <0.538 | <0.107 | <0.145 | <0.538 | <0.116 | <0.538 | <0.538 | <0.0928 | <0.423 | <0.112 | <0.106 | <1.21 | <0.189 |
| U2-10 | <0.0924 | <0.108 | <0.134 | <0.0902 | <0.421 | <0.0834 | <0.114 | <0.421 | <0.0907 | <0.421 | <0.421 | <0.0726 | <0.331 | <0.0873 | <0.0829 | <0.946 | <0.148 |
| U2-11 | <0.122 | <0.143 | <0.177 | <0.12 | <0.558 | <0.11 | <0.151 | <0.558 | <0.12 | <0.558 | <0.558 | <0.0961 | <0.438 | <0.116 | <0.11 | <1.25 | <0.196 |
| U2-12 | <0.147 | <0.173 | <0.213 | <0.144 | <0.671 | <0.133 | <0.181 | <0.671 | <0.145 | <0.671 | <0.671 | <0.116 | <0.527 | <0.139 | <0.132 | <1.51 | <0.236 |
| U2-13 | <0.232 | <0.271 | <0.336 | <0.226 | <1.06 | <0.209 | <0.285 | <1.06 | <0.227 | <1.06 | <1.06 | <0.182 | <0.829 | <0.219 | <0.208 | <2.37 | <0.371 |
| U2-14 | <0.163 | <0.191 | <0.236 | <0.159 | <0.743 | <0.147 | <0.201 | <0.743 | <0.16 | <0.743 | <0.743 | <0.128 | <0.584 | <0.154 | <0.146 | <1.67 | <0.261 |
| U2-15 | <0.123 | <0.144 | <0.178 | <0.12 | <0.56 | <0.111 | <0.151 | <0.56 | <0.121 | <0.56 | <0.56 | <0.0965 | <0.44 | <0.116 | <0.11 | <1.26 | <0.197 |

**TABLE S5 (b): TEQ_PCDD/F_ in the fish samples. Congeners in which the result was <MQL were replaced by 1/2MQL (n=90, pg⋅TEQ/g w.w).**

| **ID** | **2,3,7,8-TCDF** | **2,3,7,8-TCDD** | **1,2,3,7,8-PeCDF** | **2,3,4,7,8-PeCDF** | **1,2,3,7,8-PeCDD** | **1,2,3,4,7,8-HxCDF** | **1,2,3,6,7,8-HxCDF** | **1,2,3,4,7,8-HxCDD** | **2,3,4,6,7,8-HxCDF** | **1,2,3,6,7,8-HxCDD** | **1,2,3,7,8,9-HxCDD** | **1,2,3,7,8,9-HxCDF** | **1,2,3,4,6,7,8-HpCDF** | **1,2,3,4,6,7,8-HpCDD** | **1,2,3,4,7,8,9-HpCDF** | **OCDD** | **OCDF** | **TEQ_PCDD_F** |
| --- | --- | --- | --- | --- | --- | --- | --- | --- | --- | --- | --- | --- | --- | --- | --- | --- | --- | --- |
| M1-1 | 4.10E-03 | 4.80E-02 | 1.78E-03 | 1.20E-02 | 1.87E-01 | 3.70E-03 | 5.05E-03 | 1.87E-02 | 4.02E-03 | 1.87E-02 | 1.87E-02 | 3.22E-03 | 1.47E-03 | 3.88E-04 | 3.68E-04 | 1.26E-04 | 1.97E-05 | 0.327165341 |
| M1-2 | 4.81E-03 | 5.64E-02 | 2.09E-03 | 1.41E-02 | 2.19E-01 | 4.35E-03 | 5.93E-03 | 2.19E-02 | 4.72E-03 | 2.19E-02 | 2.19E-02 | 3.78E-03 | 1.72E-03 | 4.55E-04 | 4.32E-04 | 1.48E-04 | 2.31E-05 | 0.384031552 |
| M1-3 | 5.56E-03 | 6.51E-02 | 2.42E-03 | 1.63E-02 | 2.53E-01 | 5.02E-03 | 6.84E-03 | 2.53E-02 | 5.46E-03 | 2.53E-02 | 2.53E-02 | 4.37E-03 | 1.99E-03 | 5.25E-04 | 4.99E-04 | 1.71E-04 | 2.67E-05 | 0.443614022 |
| M1-4 | 4.97E-03 | 5.81E-02 | 2.16E-03 | 1.45E-02 | 2.26E-01 | 4.48E-03 | 6.11E-03 | 2.26E-02 | 4.87E-03 | 2.26E-02 | 2.26E-02 | 3.90E-03 | 1.78E-03 | 4.69E-04 | 4.45E-04 | 1.52E-04 | 2.39E-05 | 0.396102392 |
| M1-5 | 4.91E-03 | 5.75E-02 | 2.13E-03 | 1.44E-02 | 2.24E-01 | 4.43E-03 | 6.04E-03 | 2.24E-02 | 4.82E-03 | 2.24E-02 | 2.24E-02 | 3.86E-03 | 1.76E-03 | 4.64E-04 | 4.40E-04 | 1.51E-04 | 2.36E-05 | 0.391544252 |
| M1-6 | 5.60E-03 | 6.56E-02 | 2.43E-03 | 1.64E-02 | 2.55E-01 | 5.05E-03 | 6.89E-03 | 2.55E-02 | 5.49E-03 | 2.55E-02 | 2.55E-02 | 4.40E-03 | 2.00E-03 | 5.29E-04 | 5.02E-04 | 1.72E-04 | 2.69E-05 | 0.446524875 |
| M1-7 | 5.39E-03 | 6.31E-02 | 2.34E-03 | 1.58E-02 | 2.45E-01 | 4.86E-03 | 6.63E-03 | 2.45E-02 | 5.28E-03 | 2.45E-02 | 2.45E-02 | 4.23E-03 | 1.93E-03 | 5.09E-04 | 4.83E-04 | 1.65E-04 | 2.59E-05 | 0.429611054 |
| M1-8 | 4.76E-03 | 5.57E-02 | 2.07E-03 | 1.39E-02 | 2.17E-01 | 4.30E-03 | 5.86E-03 | 2.17E-02 | 4.67E-03 | 2.17E-02 | 2.17E-02 | 3.74E-03 | 1.70E-03 | 4.50E-04 | 4.27E-04 | 1.46E-04 | 2.29E-05 | 0.379745485 |
| M1-9 | 4.52E-03 | 5.30E-02 | 1.97E-03 | 1.33E-02 | 2.06E-01 | 4.08E-03 | 5.57E-03 | 2.06E-02 | 4.44E-03 | 2.06E-02 | 2.06E-02 | 3.55E-03 | 1.62E-03 | 4.27E-04 | 4.06E-04 | 1.39E-04 | 2.17E-05 | 0.360818616 |
| M1-10 | 5.16E-03 | 6.05E-02 | 2.24E-03 | 1.51E-02 | 2.35E-01 | 4.66E-03 | 6.36E-03 | 2.35E-02 | 5.07E-03 | 2.35E-02 | 2.35E-02 | 4.06E-03 | 1.85E-03 | 4.88E-04 | 4.63E-04 | 1.59E-04 | 2.48E-05 | 0.411927306 |
| M1-11 | 5.21E-03 | 6.10E-02 | 2.26E-03 | 1.53E-02 | 2.37E-01 | 4.70E-03 | 6.41E-03 | 2.37E-02 | 5.11E-03 | 2.37E-02 | 2.37E-02 | 4.09E-03 | 1.86E-03 | 4.92E-04 | 4.67E-04 | 1.60E-04 | 2.50E-05 | 0.415448053 |
| M1-12 | 2.15E-02 | 2.52E-01 | 9.36E-03 | 6.31E-02 | 9.81E-01 | 1.94E-02 | 2.65E-02 | 9.81E-02 | 2.11E-02 | 9.81E-02 | 9.81E-02 | 1.69E-02 | 7.71E-03 | 2.04E-03 | 1.93E-03 | 6.61E-04 | 1.03E-04 | 1.718444217 |
| M1-13 | 1.68E-02 | 1.97E-01 | 7.30E-03 | 4.92E-02 | 7.65E-01 | 1.52E-02 | 2.07E-02 | 7.65E-02 | 1.65E-02 | 7.65E-02 | 7.65E-02 | 1.32E-02 | 6.01E-03 | 1.59E-03 | 1.51E-03 | 5.16E-04 | 8.07E-05 | 1.339574626 |
| M1-14 | 2.42E-02 | 2.84E-01 | 1.05E-02 | 7.10E-02 | 1.10E+00 | 2.19E-02 | 2.98E-02 | 1.10E-01 | 2.38E-02 | 1.10E-01 | 1.10E-01 | 1.90E-02 | 8.67E-03 | 2.29E-03 | 2.17E-03 | 7.44E-04 | 1.16E-04 | 1.933249744 |
| M1-15 | 1.10E-02 | 1.29E-01 | 4.79E-03 | 3.23E-02 | 5.02E-01 | 9.95E-03 | 1.36E-02 | 5.02E-02 | 1.08E-02 | 5.02E-02 | 5.02E-02 | 8.66E-03 | 3.94E-03 | 1.04E-03 | 9.88E-04 | 3.38E-04 | 5.29E-05 | 0.879204018 |
| C1-1 | 3.99E-03 | 4.67E-02 | 1.73E-03 | 1.17E-02 | 1.82E-01 | 3.60E-03 | 4.91E-03 | 1.82E-02 | 3.91E-03 | 1.82E-02 | 1.82E-02 | 3.13E-03 | 1.43E-03 | 3.77E-04 | 3.57E-04 | 1.22E-04 | 1.91E-05 | 0.317992481 |
| C1-2 | 4.79E-03 | 5.61E-02 | 2.08E-03 | 1.40E-02 | 2.18E-01 | 4.33E-03 | 5.90E-03 | 2.18E-02 | 4.70E-03 | 2.18E-02 | 2.18E-02 | 3.76E-03 | 1.71E-03 | 4.53E-04 | 4.30E-04 | 1.47E-04 | 2.30E-05 | 0.382305567 |
| C1-3 | 4.79E-03 | 5.61E-02 | 2.08E-03 | 1.40E-02 | 2.18E-01 | 4.33E-03 | 5.90E-03 | 2.18E-02 | 4.70E-03 | 2.18E-02 | 2.18E-02 | 3.76E-03 | 1.71E-03 | 4.53E-04 | 4.30E-04 | 1.47E-04 | 2.30E-05 | 0.382305567 |
| C1-4 | 5.34E-03 | 6.25E-02 | 2.32E-03 | 1.56E-02 | 2.43E-01 | 4.82E-03 | 6.57E-03 | 2.43E-02 | 5.24E-03 | 2.43E-02 | 2.43E-02 | 4.19E-03 | 1.91E-03 | 5.04E-04 | 4.79E-04 | 1.64E-04 | 2.56E-05 | 0.425847253 |
| C1-5 | 5.60E-03 | 6.56E-02 | 2.43E-03 | 1.64E-02 | 2.55E-01 | 5.05E-03 | 6.89E-03 | 2.55E-02 | 5.49E-03 | 2.55E-02 | 2.55E-02 | 4.40E-03 | 2.00E-03 | 5.29E-04 | 5.02E-04 | 1.72E-04 | 2.69E-05 | 0.446524875 |
| C1-6 | 5.08E-03 | 5.95E-02 | 2.21E-03 | 1.49E-02 | 2.32E-01 | 4.59E-03 | 6.26E-03 | 2.32E-02 | 4.99E-03 | 2.32E-02 | 2.32E-02 | 3.99E-03 | 1.82E-03 | 4.80E-04 | 4.56E-04 | 1.56E-04 | 2.44E-05 | 0.405544642 |
| C1-7 | 3.65E-03 | 4.27E-02 | 1.58E-03 | 1.07E-02 | 1.66E-01 | 3.29E-03 | 4.49E-03 | 1.66E-02 | 3.58E-03 | 1.66E-02 | 1.66E-02 | 2.86E-03 | 1.30E-03 | 3.44E-04 | 3.27E-04 | 1.12E-04 | 1.75E-05 | 0.290813637 |
| C1-8 | 5.05E-03 | 5.92E-02 | 2.20E-03 | 1.48E-02 | 2.30E-01 | 4.56E-03 | 6.22E-03 | 2.30E-02 | 4.96E-03 | 2.30E-02 | 2.30E-02 | 3.97E-03 | 1.81E-03 | 4.77E-04 | 4.53E-04 | 1.55E-04 | 2.43E-05 | 0.403142127 |
| C1-9 | 4.55E-03 | 5.33E-02 | 1.98E-03 | 1.33E-02 | 2.07E-01 | 4.10E-03 | 5.60E-03 | 2.07E-02 | 4.46E-03 | 2.07E-02 | 2.07E-02 | 3.57E-03 | 1.63E-03 | 4.30E-04 | 4.08E-04 | 1.40E-04 | 2.18E-05 | 0.362741956 |
| C1-10 | 4.37E-03 | 5.12E-02 | 1.90E-03 | 1.28E-02 | 1.99E-01 | 3.94E-03 | 5.38E-03 | 1.99E-02 | 4.29E-03 | 1.99E-02 | 1.99E-02 | 3.43E-03 | 1.56E-03 | 4.13E-04 | 3.92E-04 | 1.34E-04 | 2.10E-05 | 0.348618806 |
| C1-11 | 4.52E-03 | 5.30E-02 | 1.97E-03 | 1.33E-02 | 2.06E-01 | 4.08E-03 | 5.57E-03 | 2.06E-02 | 4.44E-03 | 2.06E-02 | 2.06E-02 | 3.55E-03 | 1.62E-03 | 4.27E-04 | 4.06E-04 | 1.39E-04 | 2.17E-05 | 0.360818616 |
| C1-12 | 1.06E-02 | 1.24E-01 | 4.60E-03 | 3.10E-02 | 4.82E-01 | 9.55E-03 | 1.30E-02 | 4.82E-02 | 1.04E-02 | 4.82E-02 | 4.82E-02 | 8.31E-03 | 3.79E-03 | 1.00E-03 | 9.49E-04 | 3.25E-04 | 5.08E-05 | 0.844297655 |
| C1-13 | 6.02E-03 | 7.05E-02 | 2.61E-03 | 1.76E-02 | 2.74E-01 | 5.43E-03 | 7.40E-03 | 2.74E-02 | 5.90E-03 | 2.74E-02 | 2.74E-02 | 4.72E-03 | 2.15E-03 | 5.68E-04 | 5.39E-04 | 1.85E-04 | 2.89E-05 | 0.479904027 |
| C1-14 | 6.05E-03 | 7.09E-02 | 2.63E-03 | 1.77E-02 | 2.76E-01 | 5.46E-03 | 7.45E-03 | 2.76E-02 | 5.94E-03 | 2.76E-02 | 2.76E-02 | 4.75E-03 | 2.16E-03 | 5.72E-04 | 5.43E-04 | 1.86E-04 | 2.91E-05 | 0.482626887 |
| C1-15 | 6.88E-03 | 8.06E-02 | 2.99E-03 | 2.02E-02 | 3.13E-01 | 6.21E-03 | 8.47E-03 | 3.13E-02 | 6.75E-03 | 3.13E-02 | 3.13E-02 | 5.40E-03 | 2.46E-03 | 6.50E-04 | 6.17E-04 | 2.11E-04 | 3.30E-05 | 0.548793476 |
| U1-1 | 3.21E-03 | 3.76E-02 | 1.39E-03 | 9.39E-03 | 1.46E-01 | 2.89E-03 | 3.95E-03 | 1.46E-02 | 3.15E-03 | 1.46E-02 | 1.46E-02 | 2.52E-03 | 1.15E-03 | 3.03E-04 | 2.88E-04 | 9.85E-05 | 1.54E-05 | 0.255828538 |
| U1-2 | 3.91E-03 | 4.58E-02 | 1.70E-03 | 1.15E-02 | 1.78E-01 | 3.53E-03 | 4.82E-03 | 1.78E-02 | 3.84E-03 | 1.78E-02 | 1.78E-02 | 3.07E-03 | 1.40E-03 | 3.70E-04 | 3.51E-04 | 1.20E-04 | 1.88E-05 | 0.312157757 |
| U1-3 | 5.45E-03 | 6.39E-02 | 2.37E-03 | 1.60E-02 | 2.48E-01 | 4.92E-03 | 6.71E-03 | 2.48E-02 | 5.35E-03 | 2.48E-02 | 2.48E-02 | 4.28E-03 | 1.95E-03 | 5.15E-04 | 4.89E-04 | 1.67E-04 | 2.62E-05 | 0.435104802 |
| U1-4 | 3.55E-03 | 4.16E-02 | 1.55E-03 | 1.04E-02 | 1.62E-01 | 3.21E-03 | 4.38E-03 | 1.62E-02 | 3.49E-03 | 1.62E-02 | 1.62E-02 | 2.79E-03 | 1.27E-03 | 3.36E-04 | 3.19E-04 | 1.09E-04 | 1.71E-05 | 0.283543296 |
| U1-5 | 5.60E-03 | 6.56E-02 | 2.43E-03 | 1.64E-02 | 2.55E-01 | 5.05E-03 | 6.89E-03 | 2.55E-02 | 5.49E-03 | 2.55E-02 | 2.55E-02 | 4.40E-03 | 2.00E-03 | 5.29E-04 | 5.02E-04 | 1.72E-04 | 2.69E-05 | 0.446524875 |
| U1-6 | 5.73E-03 | 6.71E-02 | 2.49E-03 | 1.68E-02 | 2.61E-01 | 5.17E-03 | 7.06E-03 | 2.61E-02 | 5.63E-03 | 2.61E-02 | 2.61E-02 | 4.50E-03 | 2.05E-03 | 5.42E-04 | 5.14E-04 | 1.76E-04 | 2.75E-05 | 0.457327897 |
| U1-7 | 4.66E-03 | 5.46E-02 | 2.03E-03 | 1.37E-02 | 2.12E-01 | 4.21E-03 | 5.74E-03 | 2.12E-02 | 4.57E-03 | 2.12E-02 | 2.12E-02 | 3.66E-03 | 1.67E-03 | 4.40E-04 | 4.18E-04 | 1.43E-04 | 2.24E-05 | 0.37186006 |
| U1-8 | 5.52E-03 | 6.46E-02 | 2.40E-03 | 1.62E-02 | 2.51E-01 | 4.98E-03 | 6.79E-03 | 2.51E-02 | 5.41E-03 | 2.51E-02 | 2.51E-02 | 4.33E-03 | 1.97E-03 | 5.21E-04 | 4.95E-04 | 1.69E-04 | 2.65E-05 | 0.440170705 |
| U1-9 | 3.68E-03 | 4.31E-02 | 1.60E-03 | 1.08E-02 | 1.68E-01 | 3.32E-03 | 4.53E-03 | 1.68E-02 | 3.61E-03 | 1.68E-02 | 1.68E-02 | 2.89E-03 | 1.32E-03 | 3.47E-04 | 3.30E-04 | 1.13E-04 | 1.77E-05 | 0.293320651 |
| U1-10 | 4.83E-03 | 5.66E-02 | 2.10E-03 | 1.42E-02 | 2.20E-01 | 4.36E-03 | 5.95E-03 | 2.20E-02 | 4.74E-03 | 2.20E-02 | 2.20E-02 | 3.79E-03 | 1.73E-03 | 4.56E-04 | 4.33E-04 | 1.48E-04 | 2.32E-05 | 0.385336302 |
| U1-11 | 1.53E-02 | 1.79E-01 | 6.65E-03 | 4.48E-02 | 6.96E-01 | 1.38E-02 | 1.88E-02 | 6.96E-02 | 1.50E-02 | 6.96E-02 | 6.96E-02 | 1.20E-02 | 5.47E-03 | 1.44E-03 | 1.37E-03 | 4.69E-04 | 7.34E-05 | 1.219541057 |
| U1-12 | 1.59E-02 | 1.86E-01 | 6.89E-03 | 4.64E-02 | 7.22E-01 | 1.43E-02 | 1.95E-02 | 7.22E-02 | 1.56E-02 | 7.22E-02 | 7.22E-02 | 1.25E-02 | 5.67E-03 | 1.50E-03 | 1.42E-03 | 4.87E-04 | 7.62E-05 | 1.264877156 |
| U1-13 | 2.84E-02 | 3.33E-01 | 1.24E-02 | 8.33E-02 | 1.30E+00 | 2.57E-02 | 3.50E-02 | 1.30E-01 | 2.79E-02 | 1.30E-01 | 1.30E-01 | 2.23E-02 | 1.02E-02 | 2.69E-03 | 2.55E-03 | 8.73E-04 | 1.37E-04 | 2.268346367 |
| U1-14 | 1.44E-02 | 1.68E-01 | 6.24E-03 | 4.21E-02 | 6.54E-01 | 1.30E-02 | 1.77E-02 | 6.54E-02 | 1.41E-02 | 6.54E-02 | 6.54E-02 | 1.13E-02 | 5.14E-03 | 1.36E-03 | 1.29E-03 | 4.41E-04 | 6.90E-05 | 1.145629478 |
| U1-15 | 2.07E-02 | 2.42E-01 | 9.00E-03 | 6.07E-02 | 9.43E-01 | 1.87E-02 | 2.55E-02 | 9.43E-02 | 2.03E-02 | 9.43E-02 | 9.43E-02 | 1.63E-02 | 7.41E-03 | 1.96E-03 | 1.86E-03 | 6.36E-04 | 9.95E-05 | 1.651708519 |
| M2-1 | 3.81E-03 | 4.46E-02 | 1.66E-03 | 1.12E-02 | 1.73E-01 | 3.44E-03 | 4.69E-03 | 1.73E-02 | 3.74E-03 | 1.73E-02 | 1.73E-02 | 2.99E-03 | 1.36E-03 | 3.60E-04 | 3.42E-04 | 1.17E-04 | 1.83E-05 | 0.303796388 |
| M2-2 | 5.45E-03 | 6.38E-02 | 2.37E-03 | 1.60E-02 | 2.48E-01 | 4.92E-03 | 6.70E-03 | 2.48E-02 | 5.34E-03 | 2.48E-02 | 2.48E-02 | 4.28E-03 | 1.95E-03 | 5.15E-04 | 4.89E-04 | 1.67E-04 | 2.62E-05 | 0.434549112 |
| M2-3 | 4.94E-03 | 5.79E-02 | 2.15E-03 | 1.45E-02 | 2.25E-01 | 4.46E-03 | 6.08E-03 | 2.25E-02 | 4.85E-03 | 2.25E-02 | 2.25E-02 | 3.88E-03 | 1.77E-03 | 4.67E-04 | 4.43E-04 | 1.52E-04 | 2.37E-05 | 0.39426646 |
| M2-4 | 5.97E-03 | 7.00E-02 | 2.60E-03 | 1.75E-02 | 2.72E-01 | 5.39E-03 | 7.35E-03 | 2.72E-02 | 5.86E-03 | 2.72E-02 | 2.72E-02 | 4.69E-03 | 2.14E-03 | 5.64E-04 | 5.36E-04 | 1.83E-04 | 2.87E-05 | 0.476543354 |
| M2-5 | 3.71E-03 | 4.34E-02 | 1.61E-03 | 1.09E-02 | 1.69E-01 | 3.35E-03 | 4.57E-03 | 1.69E-02 | 3.64E-03 | 1.69E-02 | 1.69E-02 | 2.91E-03 | 1.33E-03 | 3.50E-04 | 3.33E-04 | 1.14E-04 | 1.78E-05 | 0.295871265 |
| M2-6 | 4.33E-03 | 5.07E-02 | 1.88E-03 | 1.27E-02 | 1.97E-01 | 3.90E-03 | 5.32E-03 | 1.97E-02 | 4.24E-03 | 1.97E-02 | 1.97E-02 | 3.40E-03 | 1.55E-03 | 4.09E-04 | 3.88E-04 | 1.33E-04 | 2.08E-05 | 0.345083119 |
| M2-7 | 1.72E-02 | 2.01E-01 | 7.48E-03 | 5.04E-02 | 7.83E-01 | 1.55E-02 | 2.12E-02 | 7.83E-02 | 1.69E-02 | 7.83E-02 | 7.83E-02 | 1.35E-02 | 6.15E-03 | 1.63E-03 | 1.54E-03 | 5.28E-04 | 8.26E-05 | 1.37198369 |
| M2-8 | 4.83E-03 | 5.66E-02 | 2.10E-03 | 1.42E-02 | 2.20E-01 | 4.36E-03 | 5.95E-03 | 2.20E-02 | 4.74E-03 | 2.20E-02 | 2.20E-02 | 3.79E-03 | 1.73E-03 | 4.56E-04 | 4.33E-04 | 1.48E-04 | 2.32E-05 | 0.385336302 |
| M2-9 | 4.95E-03 | 5.79E-02 | 2.15E-03 | 1.45E-02 | 2.25E-01 | 4.47E-03 | 6.09E-03 | 2.25E-02 | 4.85E-03 | 2.25E-02 | 2.25E-02 | 3.89E-03 | 1.77E-03 | 4.68E-04 | 4.44E-04 | 1.52E-04 | 2.38E-05 | 0.394723846 |
| M2-10 | 5.60E-03 | 6.56E-02 | 2.44E-03 | 1.64E-02 | 2.55E-01 | 5.06E-03 | 6.90E-03 | 2.55E-02 | 5.50E-03 | 2.55E-02 | 2.55E-02 | 4.40E-03 | 2.01E-03 | 5.30E-04 | 5.03E-04 | 1.72E-04 | 2.69E-05 | 0.447111636 |
| M2-11 | 2.14E-02 | 2.51E-01 | 9.32E-03 | 6.28E-02 | 9.76E-01 | 1.93E-02 | 2.64E-02 | 9.76E-02 | 2.10E-02 | 9.76E-02 | 9.76E-02 | 1.68E-02 | 7.67E-03 | 2.03E-03 | 1.92E-03 | 6.58E-04 | 1.03E-04 | 1.709808819 |
| M2-12 | 6.95E-03 | 8.14E-02 | 3.02E-03 | 2.04E-02 | 3.16E-01 | 6.27E-03 | 8.55E-03 | 3.16E-02 | 6.82E-03 | 3.16E-02 | 3.16E-02 | 5.46E-03 | 2.49E-03 | 6.56E-04 | 6.23E-04 | 2.13E-04 | 3.34E-05 | 0.554156279 |
| M2-13 | 9.00E-03 | 1.05E-01 | 3.91E-03 | 2.64E-02 | 4.10E-01 | 8.12E-03 | 1.11E-02 | 4.10E-02 | 8.83E-03 | 4.10E-02 | 4.10E-02 | 7.07E-03 | 3.22E-03 | 8.50E-04 | 8.07E-04 | 2.76E-04 | 4.32E-05 | 0.717831129 |
| M2-14 | 5.32E-03 | 6.24E-02 | 2.31E-03 | 1.56E-02 | 2.43E-01 | 4.81E-03 | 6.55E-03 | 2.43E-02 | 5.22E-03 | 2.43E-02 | 2.43E-02 | 4.18E-03 | 1.91E-03 | 5.03E-04 | 4.78E-04 | 1.64E-04 | 2.56E-05 | 0.424783964 |
| M2-15 | 5.59E-03 | 6.55E-02 | 2.43E-03 | 1.64E-02 | 2.55E-01 | 5.05E-03 | 6.88E-03 | 2.55E-02 | 5.48E-03 | 2.55E-02 | 2.55E-02 | 4.39E-03 | 2.00E-03 | 5.28E-04 | 5.01E-04 | 1.72E-04 | 2.69E-05 | 0.445939653 |
| C2-1 | 3.68E-03 | 4.31E-02 | 1.60E-03 | 1.08E-02 | 1.68E-01 | 3.32E-03 | 4.53E-03 | 1.68E-02 | 3.61E-03 | 1.68E-02 | 1.68E-02 | 2.89E-03 | 1.32E-03 | 3.47E-04 | 3.30E-04 | 1.13E-04 | 1.77E-05 | 0.293320651 |
| C2-2 | 3.33E-03 | 3.90E-02 | 1.45E-03 | 9.76E-03 | 1.52E-01 | 3.01E-03 | 4.10E-03 | 1.52E-02 | 3.27E-03 | 1.52E-02 | 1.52E-02 | 2.62E-03 | 1.19E-03 | 3.15E-04 | 2.99E-04 | 1.02E-04 | 1.60E-05 | 0.26582184 |
| C2-3 | 3.95E-03 | 4.63E-02 | 1.72E-03 | 1.16E-02 | 1.80E-01 | 3.56E-03 | 4.86E-03 | 1.80E-02 | 3.88E-03 | 1.80E-02 | 1.80E-02 | 3.10E-03 | 1.41E-03 | 3.73E-04 | 3.54E-04 | 1.21E-04 | 1.90E-05 | 0.315048106 |
| C2-4 | 1.22E-02 | 1.42E-01 | 5.28E-03 | 3.56E-02 | 5.54E-01 | 1.10E-02 | 1.50E-02 | 5.54E-02 | 1.19E-02 | 5.54E-02 | 5.54E-02 | 9.54E-03 | 4.35E-03 | 1.15E-03 | 1.09E-03 | 3.73E-04 | 5.84E-05 | 0.969378789 |
| C2-5 | 3.52E-03 | 4.13E-02 | 1.53E-03 | 1.03E-02 | 1.61E-01 | 3.18E-03 | 4.34E-03 | 1.61E-02 | 3.46E-03 | 1.61E-02 | 1.61E-02 | 2.77E-03 | 1.26E-03 | 3.33E-04 | 3.16E-04 | 1.08E-04 | 1.69E-05 | 0.281199963 |
| C2-6 | 4.31E-03 | 5.05E-02 | 1.87E-03 | 1.26E-02 | 1.96E-01 | 3.89E-03 | 5.30E-03 | 1.96E-02 | 4.23E-03 | 1.96E-02 | 1.96E-02 | 3.38E-03 | 1.54E-03 | 4.07E-04 | 3.86E-04 | 1.32E-04 | 2.07E-05 | 0.343688843 |
| C2-7 | 3.33E-03 | 3.90E-02 | 1.45E-03 | 9.76E-03 | 1.52E-01 | 3.01E-03 | 4.10E-03 | 1.52E-02 | 3.27E-03 | 1.52E-02 | 1.52E-02 | 2.62E-03 | 1.19E-03 | 3.15E-04 | 2.99E-04 | 1.02E-04 | 1.60E-05 | 0.26582184 |
| C2-8 | 4.54E-03 | 5.32E-02 | 1.97E-03 | 1.33E-02 | 2.07E-01 | 4.10E-03 | 5.59E-03 | 2.07E-02 | 4.46E-03 | 2.07E-02 | 2.07E-02 | 3.57E-03 | 1.63E-03 | 4.29E-04 | 4.07E-04 | 1.39E-04 | 2.18E-05 | 0.36235565 |
| C2-9 | 5.08E-03 | 5.95E-02 | 2.21E-03 | 1.49E-02 | 2.31E-01 | 4.58E-03 | 6.25E-03 | 2.31E-02 | 4.98E-03 | 2.31E-02 | 2.31E-02 | 3.99E-03 | 1.82E-03 | 4.80E-04 | 4.55E-04 | 1.56E-04 | 2.44E-05 | 0.405061851 |
| C2-10 | 4.36E-03 | 5.10E-02 | 1.89E-03 | 1.28E-02 | 1.98E-01 | 3.93E-03 | 5.36E-03 | 1.98E-02 | 4.27E-03 | 1.98E-02 | 1.98E-02 | 3.42E-03 | 1.56E-03 | 4.12E-04 | 3.91E-04 | 1.34E-04 | 2.09E-05 | 0.347550516 |
| C2-11 | 4.33E-03 | 5.07E-02 | 1.88E-03 | 1.27E-02 | 1.97E-01 | 3.90E-03 | 5.32E-03 | 1.97E-02 | 4.24E-03 | 1.97E-02 | 1.97E-02 | 3.40E-03 | 1.55E-03 | 4.09E-04 | 3.88E-04 | 1.33E-04 | 2.08E-05 | 0.345083119 |
| C2-12 | 4.86E-03 | 5.69E-02 | 2.11E-03 | 1.42E-02 | 2.21E-01 | 4.38E-03 | 5.98E-03 | 2.21E-02 | 4.77E-03 | 2.21E-02 | 2.21E-02 | 3.82E-03 | 1.74E-03 | 4.59E-04 | 4.36E-04 | 1.49E-04 | 2.33E-05 | 0.3875307 |
| C2-13 | 6.27E-03 | 7.35E-02 | 2.73E-03 | 1.84E-02 | 2.86E-01 | 5.66E-03 | 7.72E-03 | 2.86E-02 | 6.15E-03 | 2.86E-02 | 2.86E-02 | 4.93E-03 | 2.24E-03 | 5.93E-04 | 5.63E-04 | 1.93E-04 | 3.01E-05 | 0.500370522 |
| C2-14 | 5.05E-03 | 5.92E-02 | 2.20E-03 | 1.48E-02 | 2.30E-01 | 4.56E-03 | 6.22E-03 | 2.30E-02 | 4.96E-03 | 2.30E-02 | 2.30E-02 | 3.97E-03 | 1.81E-03 | 4.77E-04 | 4.53E-04 | 1.55E-04 | 2.43E-05 | 0.403142127 |
| C2-15 | 5.27E-03 | 6.17E-02 | 2.29E-03 | 1.54E-02 | 2.40E-01 | 4.76E-03 | 6.49E-03 | 2.40E-02 | 5.17E-03 | 2.40E-02 | 2.40E-02 | 4.14E-03 | 1.89E-03 | 4.98E-04 | 4.73E-04 | 1.62E-04 | 2.53E-05 | 0.420583381 |
| U2-1 | 5.80E-03 | 6.80E-02 | 2.52E-03 | 1.70E-02 | 2.64E-01 | 5.24E-03 | 7.14E-03 | 2.64E-02 | 5.69E-03 | 2.64E-02 | 2.64E-02 | 4.56E-03 | 2.08E-03 | 5.48E-04 | 5.20E-04 | 1.78E-04 | 2.79E-05 | 0.46292783 |
| U2-2 | 4.80E-03 | 5.63E-02 | 2.09E-03 | 1.41E-02 | 2.19E-01 | 4.34E-03 | 5.91E-03 | 2.19E-02 | 4.71E-03 | 2.19E-02 | 2.19E-02 | 3.77E-03 | 1.72E-03 | 4.54E-04 | 4.31E-04 | 1.47E-04 | 2.31E-05 | 0.383166616 |
| U2-3 | 5.07E-03 | 5.93E-02 | 2.20E-03 | 1.48E-02 | 2.31E-01 | 4.57E-03 | 6.24E-03 | 2.31E-02 | 4.97E-03 | 2.31E-02 | 2.31E-02 | 3.98E-03 | 1.81E-03 | 4.79E-04 | 4.54E-04 | 1.56E-04 | 2.43E-05 | 0.404099709 |
| U2-4 | 5.06E-03 | 5.93E-02 | 2.20E-03 | 1.48E-02 | 2.30E-01 | 4.57E-03 | 6.23E-03 | 2.30E-02 | 4.96E-03 | 2.30E-02 | 2.30E-02 | 3.97E-03 | 1.81E-03 | 4.78E-04 | 4.54E-04 | 1.55E-04 | 2.43E-05 | 0.40362035 |
| U2-5 | 3.58E-03 | 4.20E-02 | 1.56E-03 | 1.05E-02 | 1.63E-01 | 3.24E-03 | 4.41E-03 | 1.63E-02 | 3.52E-03 | 1.63E-02 | 1.63E-02 | 2.82E-03 | 1.28E-03 | 3.39E-04 | 3.21E-04 | 1.10E-04 | 1.72E-05 | 0.285926013 |
| U2-6 | 4.66E-03 | 5.45E-02 | 2.02E-03 | 1.36E-02 | 2.12E-01 | 4.20E-03 | 5.73E-03 | 2.12E-02 | 4.57E-03 | 2.12E-02 | 2.12E-02 | 3.66E-03 | 1.67E-03 | 4.40E-04 | 4.18E-04 | 1.43E-04 | 2.24E-05 | 0.371454099 |
| U2-7 | 5.79E-03 | 6.79E-02 | 2.52E-03 | 1.70E-02 | 2.64E-01 | 5.23E-03 | 7.13E-03 | 2.64E-02 | 5.69E-03 | 2.64E-02 | 2.64E-02 | 4.55E-03 | 2.07E-03 | 5.48E-04 | 5.20E-04 | 1.78E-04 | 2.78E-05 | 0.462298852 |
| U2-8 | 4.78E-03 | 5.60E-02 | 2.08E-03 | 1.40E-02 | 2.18E-01 | 4.32E-03 | 5.89E-03 | 2.18E-02 | 4.69E-03 | 2.18E-02 | 2.18E-02 | 3.76E-03 | 1.71E-03 | 4.52E-04 | 4.29E-04 | 1.47E-04 | 2.30E-05 | 0.38144838 |
| U2-9 | 5.91E-03 | 6.92E-02 | 2.57E-03 | 1.73E-02 | 2.69E-01 | 5.33E-03 | 7.27E-03 | 2.69E-02 | 5.80E-03 | 2.69E-02 | 2.69E-02 | 4.64E-03 | 2.11E-03 | 5.58E-04 | 5.30E-04 | 1.81E-04 | 2.84E-05 | 0.471263096 |
| U2-10 | 4.62E-03 | 5.41E-02 | 2.01E-03 | 1.35E-02 | 2.11E-01 | 4.17E-03 | 5.69E-03 | 2.11E-02 | 4.53E-03 | 2.11E-02 | 2.11E-02 | 3.63E-03 | 1.65E-03 | 4.37E-04 | 4.14E-04 | 1.42E-04 | 2.22E-05 | 0.368637004 |
| U2-11 | 6.12E-03 | 7.17E-02 | 2.66E-03 | 1.79E-02 | 2.79E-01 | 5.52E-03 | 7.53E-03 | 2.79E-02 | 6.00E-03 | 2.79E-02 | 2.79E-02 | 4.81E-03 | 2.19E-03 | 5.78E-04 | 5.49E-04 | 1.88E-04 | 2.94E-05 | 0.488166363 |
| U2-12 | 7.37E-03 | 8.63E-02 | 3.20E-03 | 2.16E-02 | 3.36E-01 | 6.65E-03 | 9.07E-03 | 3.36E-02 | 7.23E-03 | 3.36E-02 | 3.36E-02 | 5.79E-03 | 2.64E-03 | 6.96E-04 | 6.61E-04 | 2.26E-04 | 3.54E-05 | 0.587654499 |
| U2-13 | 1.16E-02 | 1.36E-01 | 5.04E-03 | 3.40E-02 | 5.28E-01 | 1.05E-02 | 1.43E-02 | 5.28E-02 | 1.14E-02 | 5.28E-02 | 5.28E-02 | 9.10E-03 | 4.15E-03 | 1.10E-03 | 1.04E-03 | 3.56E-04 | 5.57E-05 | 0.924597704 |
| U2-14 | 8.15E-03 | 9.55E-02 | 3.54E-03 | 2.39E-02 | 3.72E-01 | 7.36E-03 | 1.00E-02 | 3.72E-02 | 8.00E-03 | 3.72E-02 | 3.72E-02 | 6.41E-03 | 2.92E-03 | 7.71E-04 | 7.31E-04 | 2.50E-04 | 3.92E-05 | 0.650577352 |
| U2-15 | 6.15E-03 | 7.20E-02 | 2.67E-03 | 1.80E-02 | 2.80E-01 | 5.55E-03 | 7.56E-03 | 2.80E-02 | 6.03E-03 | 2.80E-02 | 2.80E-02 | 4.83E-03 | 2.20E-03 | 5.81E-04 | 5.51E-04 | 1.89E-04 | 2.95E-05 | 0.490276592 |


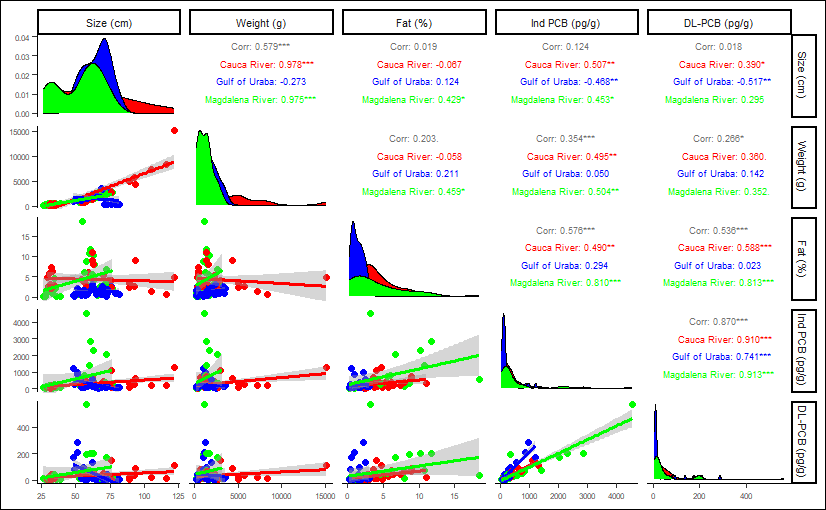


**FIGURE S1 a: Relationship between** $\sum\boldsymbol{}$**PCB_6_ and** $\sum\boldsymbol{}$**dl-PCB regarding the samples source.**


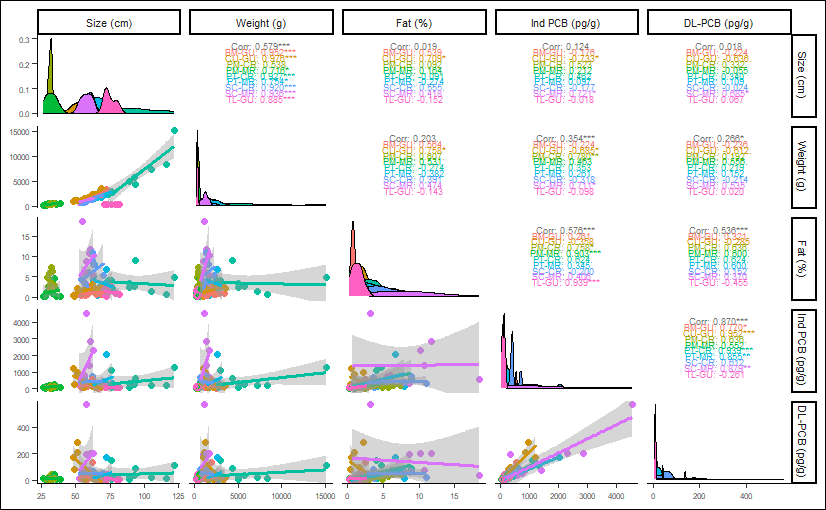


**FIGURE S1 b: Relationship between** $\sum\boldsymbol{}$**PCB_6_ and** $\sum\boldsymbol{}$**dl-PCB regarding the fish species.**
